# Formation of Bifunctional Octasilsesquioxanes via Silylative Coupling and Cross-Metathesis Reaction †

**DOI:** 10.3390/ma13183966

**Published:** 2020-09-08

**Authors:** Małgorzata Bołt, Patrycja Żak, Beata Dudziec, Anna Schulmann, Bogdan Marciniec

**Affiliations:** 1 Department of Organometallic Chemistry, Faculty of Chemistry, Adam Mickiewicz University in Poznan, Uniwersytetu Poznańskiego 8, 61-614 Poznan, Poland; malgorzata.bolt@amu.edu.pl (M.B.); anna.schulmann@amu.edu.pl (A.S.); 2 Centre for Advanced Technologies, Adam Mickiewicz University in Poznan, Uniwersytetu Poznańskiego 10, 61-614 Poznan, Poland; bogdan.marciniec@amu.edu.pl

**Keywords:** silsesquioxanes, bifunctional silsesquioxanes, cross-metathesis, silylative coupling, catalysis

## Abstract

Bifunctional silsesquioxanes create an attractive group of compounds with a wide range of potential applications, and recently they have gained much interest. They are known to be obtained mainly via hydrosilylation, but we disclose novel synthetic protocols based on different but complementary reactions, i.e., cross-metathesis (CM) and silylative coupling (SC). A series of cubic T_8_ type silsesquioxane derivatives with a broad scope of styryl substituents were synthesized in a one-pot procedure and characterized by spectroscopic and spectrometric methods. All of the new compounds can be obtained in a one-pot manner, which has an attractive impact on the synthetic procedure, as it is economic in terms of the isolation of intermediate products. Additionally, the methodology disclosed here enables the (*E*)-stereoselective introduction of styrenes derivative to the cubic T_8_ type core. The presented compounds can be interesting precursors for a further functionalization that may significantly increase the possibility of their application in the design and synthesis of new functional materials.

## 1. Introduction

Polyhedral oligosilsesquioxanes with the general formula (RSiO_3/2_)_n_ are currently one of the most attractive organosilicon compounds [1,2]. Among many advantages of these compounds, i.e., exclusive properties derived from a chemically and thermally robust organic-inorganic framework, and tailor-made three-dimensional structures, the ease of their modification by many synthetic methods is of the utmost importance. The simple functionalization of the inorganic core, as well as of the organic moieties attached to it, allows one to create various structures and to control the properties of the obtained materials. Among the highly valuable synthetic procedures for the functionalization of silsesquioxanes (SQs), we can list Heck coupling [3,4,5], Sonogashira coupling [6], hydrosilylation [7,8,9,10,11,12,13,14,15], cross-metathesis (CM) [16,17,18,19], silylative coupling (SC) [20], *O*-silylation [21,22] and Friedel–Crafts [23]. They are all dependent on the type of the functional group attached to the Si–O–Si (SiHC=CH_2_, SiH SiOH). The utilization of these multiple protocols provides a wide range of inorganic/organic hybrid systems that find great application potential in material chemistry, optics, electronics, and catalysis, as well as in medical science [2,24,25,26].

Among the many different types of silsesquioxanes, the cubic T_8_ group of molecules has still been the most common in research. Lately, special interest has shifted towards silsesquioxanes with mixes of functional groups connected to the inorganic core. The presence of different organic/organometallic moieties anchored onto the Si–O–Si core opens possibilities for the formation of a novel generation of SQs-based systems with a simultaneous control of their properties, resulting from the type and number of functional groups. In 1996, Sellinger and Laine performed a hydrosilylation reaction on both (HSiO_3/2_)_8_ and (HMe_2_SiOSiO_3/2_)_8_ with four equivalents of methacrylate, leaving four core hydrogen atoms unreacted [27]. Since then, some different attempts have been made to find an effective path for multifunctional SQ’s synthesis. However, the reports on this are still limited in the literature. Nevertheless, hydrosilylation remains one of the most useful methods for obtaining bifunctional silsesquioxanes, and it has been the subject of multiple research papers [7,8,10,12]. Heck coupling [28,29,30,31] and condensation reactions [32,33,34,35,36] were also applied in this type of synthesis. There are also single notes on the utilization of thiol-ene [37], hydration [38,39] and click type reactions [40,41,42] for the synthesis of multifunctionalized silsesquioxanes. As stated, bifunctional SQ’s derivatives, due to the presence of different functionalities in the same core, possess a high application potential, e.g., as hydrophobic materials [43,44,45,46], mold material for nanoimprint lithography [47], anion sensors [31,48], Janus star polymers [49,50] or platforms for anchoring and delivering biologically active compounds in biomedicine [51]. Bifunctional silsesquioxanes with a T_8_R_4_R’_4_ formula are of particular scientific interest because it was demonstrated that they can be considered to be Janus-type molecules [52]. However, all of these procedures lead to the formation of a mixture of products with functional groups statistically distributed on the SQ cage, and maintaining control of this process remains barely possible. The isolation of a respective product may also be complicated and not always possible. Among these synthetic protocols elaborated to obtain bifunctional SQs, the reports on the application of silylative coupling [53] to yield these compounds are still very scanty, and there is no information on the utilization of cross-metathesis and silylative coupling. These two universal and efficient functionalization methods have been developed earlier in our group and can be successfully applied in the synthesis of molecular and macromolecular silsesquioxane-based compounds [17,18,20,54,55]. Their mechanisms are slightly different, but both of them to generally lead to the same main products.

**Figure 1:**
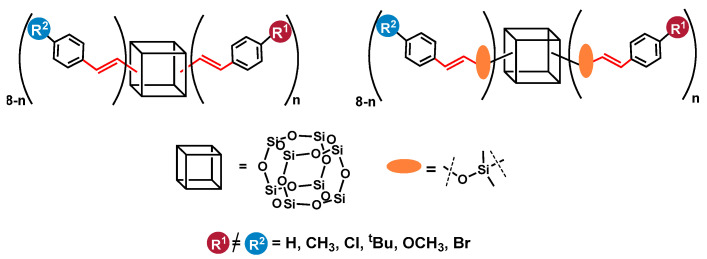
Silsesquioxanes with mixed functional groups obtained in this work.

Herein, we report on the efficient and selective bifunctionalization of octa(vinyl)silsesquioxanes (OVS–CH_2_=CH–)_8_Si_8_O_12_) and octakis(dimethylvinylsilyloxy)silsesquioxane (Q_8_M_8_–(CH_2_=CH–SiMe_2_–)_8_Si_8_O_12_) via cross-metathesis and silylative coupling processes with styrene derivatives. We decided to show the aspects of the synthetic procedures using respective Ru-based catalytic systems for the formation of the desired products. Additionally, the details of the processes affecting the distribution of the introduced functional groups in the obtained compounds were verified. Finally, the aim of this report was the efficient synthesis of novel silsesquioxanes with mixed functionalities in the course of a one-pot procedure, which was also a unique synthetic approach in the case of cross-metathesis and an innovation for silylative coupling and cubic T_8_ type SQs (Figure 1). 

We intended to obtain a spectrum of SQ derivatives with various styryl moieties, especially with unreactive and reactive R_1_, R_2_ groups, enabling their further modifications.

## 2. Materials and Methods

### 2.1. Materials

The chemicals were obtained from the following sources: styrene, 4-chlorostyrene, 4-methoxystyrene, 4-bromostyrene, 4-methylstyrene, 4-tert-butylstyrene, dichloromethane, chloroform-d, copper(I) chloride, anhydrous magnesium sulphate, calcium hydride, silica gel 60 Å, Celite^®^ from Aldrich (Sigma-Aldrich, St. Louis, MO, USA), toluene, acetone, n-hexane from Chempur, octavinylsilsesquioxane (OVS) and octakis(dimethylvinylsilyloxy)silsesquioxane ((ViSiMe_2_O)_8_Si_8_O_12_) from Hybrid Plastics (Hattiesburg, MS, USA), first-generation Grubbs’ catalyst from Apeiron Synthesis. [RuHCl(CO)(SIDip)(PCy_3_)] was prepared according to the literature procedure [56]. All solvents were dried prior to use over CaH_2_ and stored under argon. CH_2_Cl_2_ was additionally passed through a column with alumina, and then it was degassed by repeated freeze-pump-thaw cycles. Unless mentioned otherwise, all operations were performed by using standard Schlenk techniques.

### 2.2. Nuclear Magnetic Resonance Spectroscopy (NMR)

^1^H, ^13^C NMR and ^29^Si NMR spectra were recorded on Bruker Ultra Shield 600 (Faellanden, Switzerland) operating at 400, 100 and 79 MHz, respectively. CDCl_3_ was used as a solvent in all measurements. The chemical shifts are reported in ppm, referenced to the residual solvent signal (CDCl_3_).

### 2.3. Matrix-Assisted Ultraviolet Laser Desorption/Ionization Time-of-Flight Mass Spectroscopy (MALDI-TOF-MS)

MALDI-TOF mass spectra were recorded on a UltrafleXtreme mass spectrometer (Bruker Daltonics, Bremen, Germany), equipped with a SmartBeam II laser (355 nm) in the 500–4000 *m*/*z* range. 2,5-Dihydroxybenzoic acid (DHB, Bruker Daltonics, Bremen, Germany) served as the matrix and was prepared in TA30 solvent (30:70 *v*/*v* acetonitrile: 0.1% TFA in water) at a concentration of 20 mg/mL. The studied samples were dissolved in dichloromethane (2 mg/mL) and then mixed in a ratio 1:1 *v*/*v* with a matrix solution. The matrix/sample mixtures (1 μL) were spotted onto the MALDI target and dried in air. The mass spectra were measured in reflection mode. The data were analyzed using the software provided with the Ultraflex instrument-FlexAnalysis (version 3.4). The mass calibration (cubic calibration based on five to seven points) was performed using external standards (Peptide Calibration Standard).

### 2.4. FT-IR Spectroscopy

Fourier Transform-Infrared (FT-IR) spectra were recorded on a Nicolet iS5 (Thermo Scientific, Waltham, MA, USA) spectrophotometer equipped with a diamond ATR unit. In all cases, 16 scans at a resolution of 2 cm^−1^ were collected to record the spectra in a range of 4000–650 cm^−1^.

### 2.5. Synthesis of Bifunctional Silsesquioxanes via Cross-Metathesis

A 5-mL glass reactor equipped with a reflux condenser and connected to an argon/vaccum line was charged with octavinylsilsesquioxane (0.1 g, 1.6 × 10^−4^ mol), CH_2_Cl_2_ (3 mL) and styrene or 4-substituted styrene (n × 1.6 × 10^−4^ mol, the amounts depend on the required silsesquioxane: olefin molar ratio). The mixture was warmed up to 45 °C in an oil bath, and a first-generation Grubbs’ catalyst (n × 1.6 × 10^−6^ mol) was added under argon. The reaction mixture was heated under reflux until a full conversion of the substrates was detected by Thin Layer Chromatography (TLC) (typically 6–9 h depending on the styrene used). Next, the solvent was evaporated under vacuum, and cold methanol (2 mL) was added to the remains to form a white precipitate. The precipitate was filtered off, dissolved in hexane and purified by column chromatography (Silica Gel, *n*-hexane/DCM = 9:1) to remove traces of ruthenium complexes. The evaporation of the solvent gave an analytically pure sample (white powder). The isolated product was then analyzed and used for the next functionalization with a different type of styrene. The reaction and isolation conditions remained the same.

### 2.6. Synthesis of Bifunctional Silsesquioxanes via Cross-Metathesis in a One-Pot Manner

A 5-mL glass reactor equipped with a reflux condenser and connected to an argon/vacuum line was charged with octavinylsilsesquioxane (0.1 g, 1.6 × 10^−4^ mol), CH_2_Cl_2_ (3 mL) and the first type of styrene or 4-substituted styrene (n × 1.6 × 10^−4^ mol, the amounts depend on the required silsesquioxane: olefin molar ratio). The mixture was warmed up to 45 °C in an oil bath, and a first-generation Grubbs’ catalyst (n × 1.6 × 10^−6^ mol) was added under argon. The reaction mixture was heated under reflux until a full conversion of the substrates was detected by TLC (typically 6–9 h depending on the styrene used). After this time, the second type of styrene ((8 − n) × 1.6 × 10^−4^ mol) and the second portion of the catalyst ((8 − n) × 1.6 × 10^−6^ mol) was added. The reaction mixture was left for 15 h. Next, the solvent was evaporated under vacuum, and cold methanol (2 mL) was added to the remains to form a white precipitate. The precipitate was filtered off, dissolved in hexane and purified by column chromatography (Silica Gel, *n*-hexane/DCM = 9:1, Fluka Chemie AG, Buchs, Switzerland) to remove traces of ruthenium complexes. The evaporation of the solvent gave an analytically pure sample (white powder).

### 2.7. Synthesis of Bifunctional Silsesquioxanes via Silylative Coupling

A 5-mL glass reactor equipped with a reflux condenser and connected to an argon/vacuum line was charged with octakis(dimethylvinylsilyloxy)silsesquioxane (0.1 g, 1.6 × 10^−4^ mol), CH_2_Cl_2_ (3 mL) and styrene or 4-substituted styrene (n × 1.6 × 10^−4^ mol, the amounts depend on the required silsesquioxane: olefin molar ratio). The mixture was warmed up to 45 °C in an oil bath, and [RuHCl (CO)(SIDip)(PCy_3_)] (n × 1.6 × 10^−6^ mol) was added under argon. After 5 min, CuCl (5 × n × 1.6 × 10^−6^ mol) was added to the reactor as a cocatalyst. The reaction mixture was heated under reflux until a full conversion of the substrates was detected by TLC (typically 6–9 h depending on the styrene used, Sigma-Aldrich, St. Louis, MO, USA). Next, the solvent was evaporated under vacuum, and cold methanol (2 mL) was added to the remains to form a white precipitate. The precipitate was filtered off, dissolved in hexane and purified by column chromatography (Silica Gel, *n*-hexane/DCM = 9:1) to remove traces of ruthenium complexes. The evaporation of the solvent gave an analytically pure sample (white powder). The isolated product was then analyzed and used for the next functionalization with a different type of styrene. The reaction and isolation conditions remained the same.

### 2.8. Synthesis of Bifunctional Silsesquioxanes via Silylative Coupling in a One-Pot Manner

A 5-mL glass reactor equipped with a reflux condenser and connected to an argon/vacuum line was charged with octakis(dimethylvinylsilyloxy)silsesquioxane (0.1 g, 1.6 × 10^−4^ mol), CH_2_Cl_2_ (3 mL) and the first type of styrene or 4-substituted styrene (n × 1.6 × 10^−4^ mol, the amounts depend on the required silsesquioxane: olefin molar ratio). The mixture was warmed up to 45 °C in an oil bath, and [RuHCl(CO)(SIDip)(PCy_3_)] (n × 1.6 × 10^−6^ mol) was added under argon. After 5 min, CuCl (5 × n × 1.6 × 10^−6^ mol) was added to the reactor as a cocatalyst. The reaction mixture was heated under reflux until a full conversion of the substrates was detected by TLC (typically 6–9 h depending on the styrene used). After this time, the second type of styrene ((8 − n) × 1.6 × 10^−4^ mol), catalyst ((8 − n) × 1.6 × 10^−6^ mol) and CuCl (5 × (8 − n) × 1.6 × 10^−6^ mol) were added. The reaction mixture was left for 15 h. Next, the solvent was evaporated under vacuum, and cold methanol (2 mL) was added to the remains to form a white precipitate. The precipitate was filtered off, dissolved in hexane and purified by column chromatography (Silica Gel, *n*-hexane/DCM = 9:1) to remove traces of ruthenium complexes. The evaporation of the solvent gave an analytically pure sample (white powder).

## 3. Results and Discussion

### 3.1. Functionalization of Octa(vinyl)silsesquioxanes (OVS) via Cross-Metathesis Reaction

**Scheme 1:**
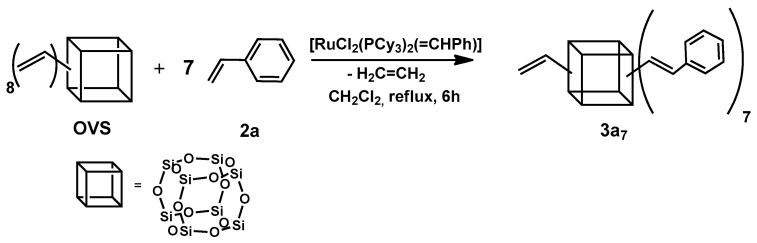
The first step of the sequential cross-metathesis on octa(vinyl)silsesquioxanes.

In the first step, we examined the possibility of obtaining a non-fully functionalized silsesquioxane core with one free vinyl group. Treatment of the 1 equiv. octavinylsilsesqioxane (OVS) with 7 equiv. of styrene (2a) in CH_2_Cl_2_ at 45 °C in the presence of the first-generation Grubbs’ catalyst gave rise to the slow evolution of ethylene and formation of heptasubstituted silsesquioxane (3a_7_) (Scheme 1).

The reaction progress was monitored by TLC, due to the large mass of the product eliminating the possibility of using GC or GC-MS. For all of the tested styrenes, a nearly complete conversion was observed up to 9 h. In the ^1^H NMR spectrum registered after this time, the only signals observed derived from a product 3a_7_ corresponding to the presence of seven ethenyl moieties and one vinyl group in the molecule. The protons from ethenyl and the free vinyl group gave only one multiplet in the range between 6.10 and 6.38 ppm, which made it impossible to define the geometry of the newly formed double bonds (Scheme 1). However, in the case of octastyrylfunctionalized silsesquioxanes, the cross-metathesis was stereoselective towards the *E*-isomers [17].

**Scheme 2:**
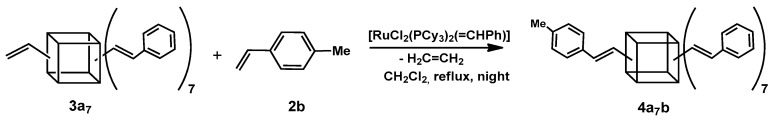
The second step of the sequential cross-metathesis on octa(vinyl)silsesquioxanes.

To confirm the product’s structure, we conducted a reaction between 3a_7_ and one equivalent of 4-methylstyrene (2b). The analysis of the ^1^H NMR spectrum revealed the presence of two separate doublets with ca. *J_H-H_* = 19 Hz. This result indicated the formation of a product with an *E* geometry of newly formed double bonds, which stood in agreement with our earlier research [17,20]. It also confirmed the functionalization of the silsesquioxane core with two different styrenes in the 7:1 molar ratio (Scheme 2).

**Figure 2:**
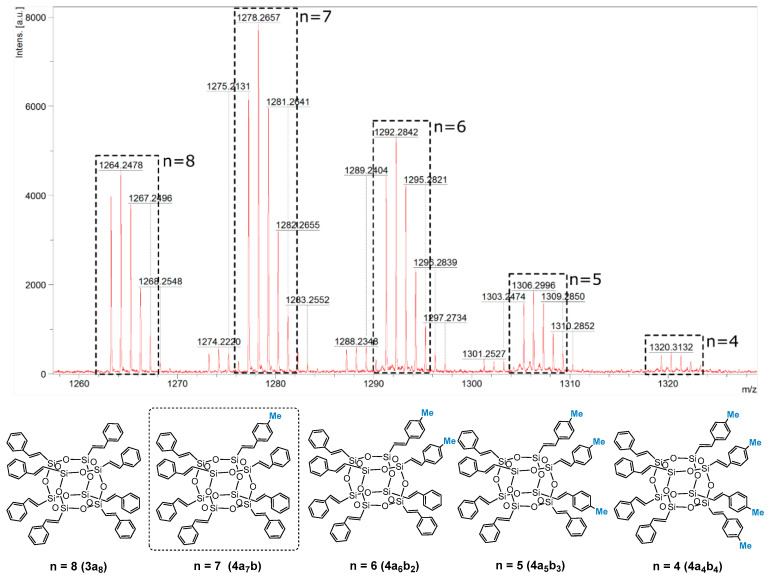
MALDI-TOF and structures with respective styrene substitutions present in the reaction mixture (Me–C_6_H_4_–CH=CH–)_8−n_(C_6_H_5_–CH=CH–)_n_Si_8_O_12_ (where n = 8, 7, 6, 5, 4) with a predominance of the designated 4a_7_b.

The MALDI-TOF analysis indicated the formation of a mixture of statistical substitution (Me–C_6_H_4_–CH=CH–)_8−n_(C_6_H_5_–CH=CH–)_n_Si_8_O_12_ (where n = 8, 7, 6, 5, 4) and the presence of respectively functionalized compounds at a ratio of 23:40:26:9:2, with a predominance of the desired compound 4a_7_b (Me–C_6_H_4_–CH=CH–)_8−n_(C_6_H_5_–CH=CH–)_n_Si_8_O_12_ (where n = 7) (which can occur with different constitutional isomers) [52] (Figure 2). This result is also evidence of the statistical substitution in the case of 3a_7_, where a complete substitution of vinyl groups could be observed (3a_8_), which was also confirmed by MALDI-TOF.

**Scheme 3:**
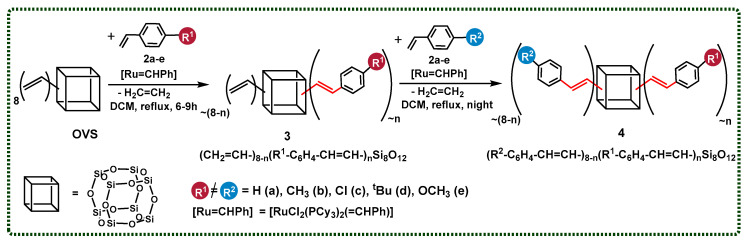

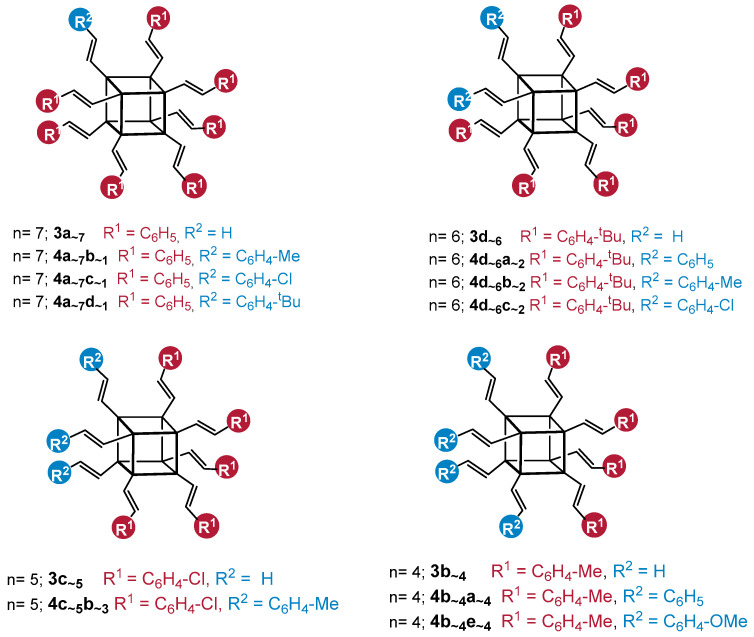
Two-step functionalization of octa(vinyl)silsesquioxane via cross-metathesis, resulting in the formation of 3 (CH_2_=CH–)_8−n_(R^1^C_6_H_5_–CH=CH–)_n_Si_8_O_12_ and 4 ((R^2^–C_6_H_4_–CH=CH–)_8−n_(R^1^–C_6_H_4_–CH=CH–)_n_Si_8_O_12_). Reaction conditions: DCM, 1st generation Grubbs catalyst, [Ru] = 1.00 mol%, reflux, argon. The molecules designed to be obtained by the reaction’s stoichiometry are presented.

After the first positive results, we decided to apply a cross-metathesis reaction to the functionalization of the SQ core with different types of styrene derivatives. We also used different ratios of OVS and two types of styrenes to discover whether the stoichiometry changes were the only factor that might affect the control of the reaction products. In all cases, the nuclear magnetic resonance analysis confirmed the exclusive formation of the desired product with an *E*-geometry of the C=C bond. It is worth mentioning that the reaction can be conducted in two different ways: with the isolation of the first substitution product 3 (CH_2_=CH–)_8−n_(R^1^C_6_H_5_–CH=CH–)_n_Si_8_O_12_, or in a one-pot manner that leads directly towards obtaining the product 4 R^2^–C_6_H_4_–CH=CH–)_8−n_(R^1^–C_6_H_4_–CH=CH–)_n_Si_8_O_12_, without isolation. In this way, the second type of styrene derivative and the next portion of the catalyst was added after 6–9 h, and the process was maintained for the next 15 h (Scheme 3).

In all cases, the conversion of the initial OVS was estimated at >99%, which was proven by ^1^H NMR analysis. The statistical substitution of two types of styrenes is observed in each reaction mixture sample, which is also consistent with the literature [7,8,12]. The respective MALDI-TOF analyses that were performed for each reaction sample revealed the presence of a signal assigned to the desired product (of the depicted stoichiometry—respectively abbreviated), accompanied by signals identified as the product of higher and lower values of the desired substitution. Herein, to clarify the results’ demonstration, the products of the desired value of substitution (designated by the applied reagents’ stoichiometry) were shown (Scheme 3). For clarity, one constitutional isomer is presented for each structure. 

All of the products were fully characterized by NMR, which confirmed the formation of targeted silsesquioxanes. ^1^H, ^13^C and ^29^Si NMR spectra of the isolated compounds are available in ESI. The analysis of the ^29^Si NMR spectra reveals resonance lines that shifted towards a higher field when compared with the silsesquioxanes described before [57,58]. The reason for this is the presence of the C=C double bond at the closest vicinity to the cage silicon atoms and its shielding effect for these atoms. The same was observed previously in the case of monovinylfunctionalized cubic silsesquioxanes [17,20,57,58]. We observed the same phenomena in the case of functionalized octaspherosilicates, described in the following section (for details, see ESI).

### 3.2. Functionalization of Octakis(dimethylvinylsilyloxy)silsesquioxane (Q_8_M_8_)-Silylative Coupling

**Scheme 4:**
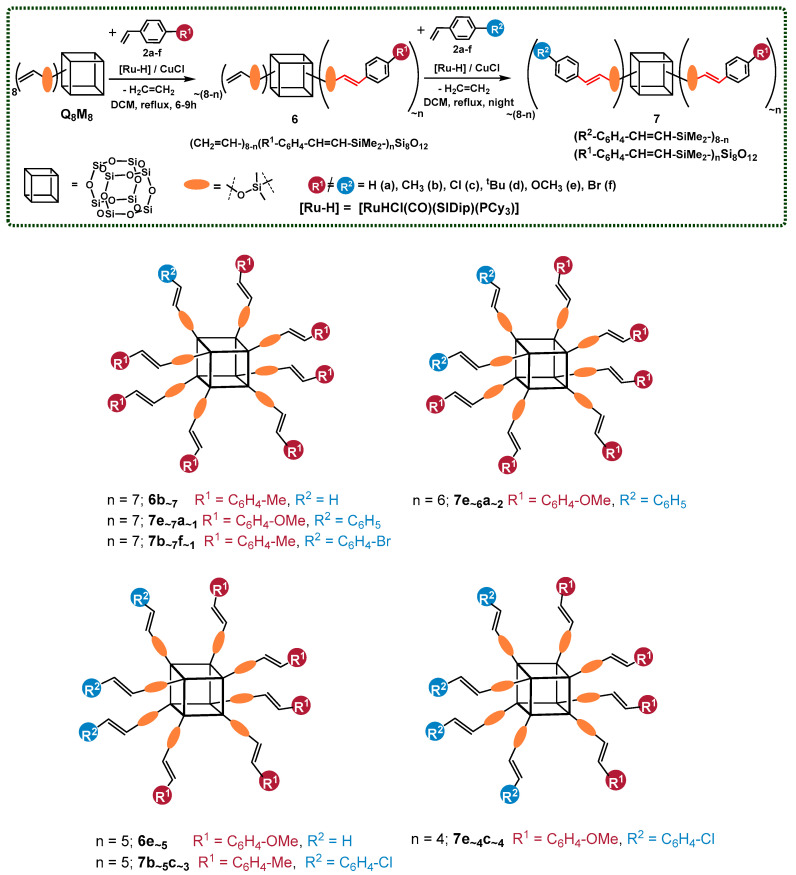
Two-step functionalization of octakis(dimethylvinylsilyloxy)silsesquioxane via silylative coupling, resulting in the formation of 6 (CH_2_=CH–SiMe_2_–)_8−n_(R^1^–C_6_H_4_–CH=CH–SiMe_2_–)_n_Si_8_O_12_ and 7 (R^2^–C_6_H_4_–CH=CH–SiMe_2_–)_8−n_(R^1^–C_6_H_4_–CH=CH–SiMe_2_–)_n_Si_8_O_12_). Reaction conditions: dichloromethane (DCM), [RuHCl(CO)(SIDip)(PCy_3_)]/CuCl, [Ru]:[Cu] = 1:5, [Ru] = 1.00 mol%, reflux, argon. The molecules designed to be obtained by the reaction’s stoichiometry are presented.

One of the limitations of the cross-metathesis of vinylsilanes is that it cannot be applied to molecules with two methyl groups connected to the silicon atom. Thus, for the effective functionalization of octakis(dimethylvinylsilyloxy)silsesquioxane (ocatvinylspherosilicate) ((CH_2_=CH–SiMe_2_–)_8_Si_8_O_12_), silylative coupling was applied as a functionalization method. The reactions were performed in the presence of an NHC ruthenium-hydride catalyst [RuHCl(CO)(SIDip)(PCy_3_)] and CuCl as a cocatalyst. Various ratios of styrene derivatives (2a–f) were applied to obtain a broad scope of bifunctional silsesquioxanes. As in the case of cross-metathesis, the desired products of the silylative coupling reaction (7 (R^2^–C_6_H_4_–CH=CH–SiMe_2_–)_8−n_(R^1^–C_6_H_4_–CH=CH–SiMe_2_–)_n_Si_8_O_12_)) can be synthesized in a one-pot manner, in two-step consecutive processes, without decreasing the effectiveness and isolation yields (Scheme 4).

In addition, to clarify the results’ demonstration, the products of the desired value of substitution (designated by the applied reagents’ stoichiometry) were shown here (Scheme 4) (in one constitutional isomer for each structure).

**Figure 3:**
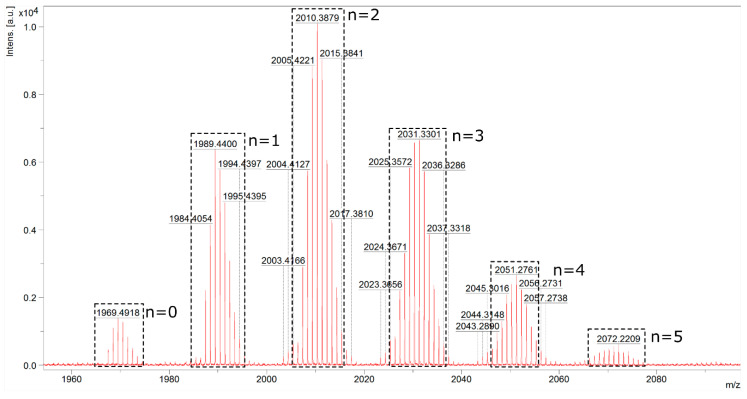
MALDI-TOF of the reaction mixture (Me–C_6_H_4_–CH=CH–SiMe_2_–)_8−n_(Cl–C_6_H_5_–CH=CH–SiMe_2_–)_n_Si_8_O_12_ (where n = 0–5) of the designated 7b_5_c_3_.

Analogically, in each example, a >99% conversion of Q_8_M_8_ was proven by ^1^H NMR. The formation of the unsymmetrical products 7 with an exclusive (*E*)-stereochemistry around newly formed Si–HC=CH–Ar double bonds was obtained (confirmed on the basis of ^1^H NMR and *J_HH_* calculated at ca. 19 Hz). A competitive polymerization of the styrene monomers was never observed. Furthermore, in this case, the statistical substitution of the tested substrates (2a–f) was observed, which was confirmed via MALDI-TOF analysis. An exemplary spectrum of the reaction mixture (Me–C_6_H_4_–CH=CH–SiMe_2_–)_8−n_(Cl–C_6_H_5_–CH=CH–SiMe_2_–)_n_Si_8_O_12_, designated for n = 3 (7b_5_c_3_), is presented in Figure 3.

**Figure 4:**
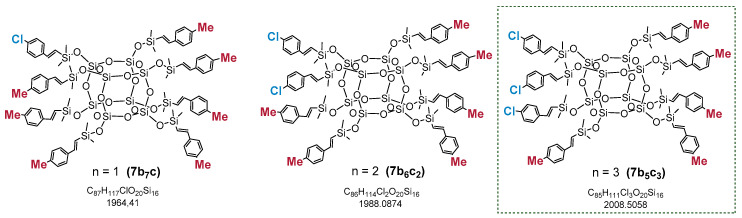
Possible structures derived from statistical substitution and the most abundant compounds in the reaction mixture of (Me–C_6_H_4_–CH=CH–SiMe_2_–)_8−n_(Cl–C_6_H_5_–CH=CH–SiMe_2_–)_n_Si_8_O_12_ (where n = 1, 2, 3) 7b_5_c_3_, analyzed by MALDI-TOF.

The analysis confirmed the statistical distribution of two types of styrene moieties (2b and 2c) attached to the Si–O–Si core in the tested sample. The masses observed in the spectra came from sodiated silsesquioxanes molecules. The three most abundant compounds are presented in Figure 4 with their molecular weights. 

The calculated masses of the sodiated molecules of the respective molecules are: C_87_H_117_ClO_20_Si_16_Na (n = 1, 7b_7_c_1_)—1987.40, C_86_H_114_Cl_2_O_20_Si_16_Na (n = 2, 7b_6_c_2_)—2007.35 and C_85_H_111_Cl_3_O_20_Si_16_Na (n = 3, 7b_5_c_3_)—2027.29. The MALDI-TOF spectrum presented in Figure 3 shows the statistical distribution of bifunctional SQs, with the most intensive signal corresponding to a 2:6 substitution of the styrenes moieties (2b and 2c) used in the reaction. The products (7) were fully characterized by ^1^H, ^13^C and ^29^Si NMR to confirm their structures, and the spectra are available in (Electronic Supporting Information) ESI (see Supplementary Materials).

The statistical distribution of the functional groups around the SQ’s core was observed in every sample that was tested by MALDI-TOF analysis, but generally with the presence of the product with the desired styryl moieties’ ratio in the majority, designated in the reaction stoichiometry. This confirms the fact that the reaction stoichiometry affects the ratio of the functional groups attached to the inorganic core. The presence of all substitution products and their respective ratio in each sample was presented in ESI. The observation of the presence of products with the statistical distribution of the functional moieties is not surprising, due to previous scientific reports that prove that the control of precise vertices’ functionalization may usually be problematic [7,8,12,28].

## 4. Conclusions

To conclude, we described the synthesis and characterization of twenty different bifunctional silsesquioxanes containing various 4-substituted styryl groups. The analysis of ^1^H NMR spectra proved the formation of C=C bonds with an *E* geometry, and MALDI-TOF showed the statistical distribution of compounds with two functional groups in 1:7, 2:6, 3:5 and 4:4 ratios, but with the highest abundance of the desired form being controlled by the reaction conditions and reagents’ stoichiometry. A further modification of the obtained compounds can lead to multibranched molecules and find application in the synthesis of dendrimers, star-shaped polymers or hydrophobic agents. Despite the statistical substitution and arrangement of the functional groups in the Si–O–Si, the resulting structures, especially in the case of partially substituted bromostyryl- and vinyl- derivatives, seem to be very interesting starting materials for further modifications. 

## Supplementary Materials

The following are available online at https://www.mdpi.com/1996-1944/13/18/3966/s1, Figures S1–S64: ^1^H, ^13^C, ^29^Si NMR and MALDI-TOF spectra of obtained compounds.
