# Supplementary material for: Formation of Bifunctional Octasilsesquioxanes via Silylative Coupling and Cross-Metathesis Reaction
Source: Materials (Basel). 2020 Sep 8;13(18):3966. doi: 10.3390/ma13183966 (PMC7557879; doi:10.3390/ma13183966)
Supplement: Supplementary file 1 [file materials-13-03966-s001.pdf]

materials

Supplementary Materials

Formation of Bifunctional Octasilsesquioxanes via Silylative Coupling and Cross-Metathesis Reaction

Contents:

1. Analytical data of products of cross metathesis. S-2
2. Analytical data of products of silylative coupling. S-5
3. NMR spectra of isolated products. S-9
   3.1. NMR spectra of cross metathesis products. S-9
   3.2. NMR spectra of silylative coupling products. S-28
4. MALDI-TOF spectra of selected compounds S-39

1. Analytical Data of Cross Metathesis Products

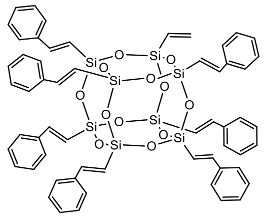

3a$_{\sim7}$

White solid

^{1}H NMR (CDCl3, δ, ppm): 6.10-6.38 (m, 10H, =*C*H-Si and =*C*H_2), 7.30-7.42 (m, 28H, -C_6H_5), 7.50 (d, 14H, J_{HH} = 7.5 Hz, =*C*H and -C_6H_5); ^{13}C NMR (CDCl3, δ, ppm): 117.48, 117.54, 127.10, 128.73, 129.07, 137.51, 149.33, 149.36; ^{29}Si NMR ($CDCl_3$, δ, ppm): -78.78, -78.82, -80.54; IR (ATR, cm^{-1}): 3058.32, 3022.83 (C-H phenyl), 2960.50 (C-H), 1603.92 (C=C), 1574.19 (C=C), 1494.20 (C=C), 1447.41 (C=C), 1406.82 (C=C), 1290.13 (Si-C), 1220.39, 1197.69, 1076.21 (Si-O), 989.38, 848.28, 816.10, 727.64, 686.43, 551.72, 463.53; MALDI-TOF MS (m/z, $[M+Na]^+$): found: 1036.13 (4), 1111.16 (45), 1188.19 (43), 1264.22 (38); calculated respectively for: $C_{46}H_{44}O_{12}Si_8Na$ (3a$_5$): 1036.52, $C_{52}H_{48}O_{12}Si_8Na$ (3a$_6$): 1111.61, $C_{58}H_{52}O_{12}Si_8Na$ (3a$_7$): 1188.71, $C_{64}H_{56}O_{12}Si_8Na$ (3a$_8$): 1264.81

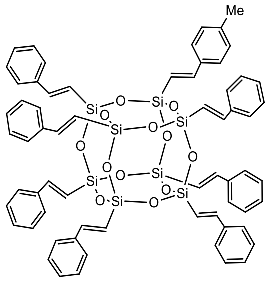

4a$_{\sim7}$b

White solid

^{1}H NMR (CDCl3, δ, ppm): 2.36 (s, 3H, -*C*H_3), 6.26 (d, 1H, J_{HH} = 19.3 Hz, =*C*H-Si), 6.32 (d, 7H, J_{HH} = 19.2 Hz, =*C*H-Si), 7.16 (d, 2H, J_{HH} = 7.9 Hz, -C_6H_4-), 7.38-7.45 (m, 32H, -C_6H_5 and -C_6H_4-), 7.50 (d, 13H, J_{HH} = 7.4 Hz, -C_6H_5 and =*C*H); **^{13}C NMR** (CDCl3, δ, ppm): 21.47 (-CH_3), 117.54, 127.05, 127.10, 128.73, 137.51, 149.34, 149.36; **^{29}Si NMR** ($CDCl_3$, δ, ppm): -78.01, -78.28, -78.30;
IR (ATR, cm^{-1}): 3057.56, 3022.74 (C-H phenyl), 3000.95 (C-H), 1603.73 (C=C), 1574.04 (C=C), 1494.17 (C=C), 1447.33 (C=C), 1334.81, 1290.14, 1220.42 (Si-C), 1197.42, 1075.61 (Si-O), 988.57, 848.37, 816.03, 727.74, 686.36, 552.85, 462.94; **MALDI-TOF MS (m/z, $[M+Na]^+$):** found: 1264.25 (23), 1278.27 (39), 1292.28 (26), 1306.30 (9) 1320.31 (2); calculated respectively for: $C_{64}H_{56}O_{12}Si_8Na$ (3a$_8$): 1264.79, $C_{65}H_{58}O_{12}Si_8Na$ (4a$_7$b): 1278.83, $C_{66}H_{60}O_{12}Si_8Na$ (4a$_6$b$_2$): 1292.85, $C_{67}H_{62}O_{12}Si_8Na$ (4a$_5$b$_3$): 1306.88, $C_{68}H_{64}O_{12}Si_8Na$ (4a$_4$b$_4$): 1320.90.

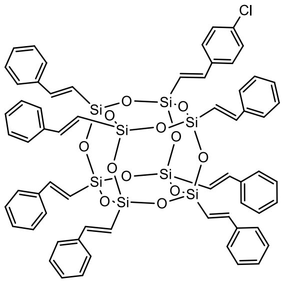

4a$_{\sim7}$c

White solid

^{1}H NMR ($CDCl_3$, δ, ppm): 6.34 (d, 8H, J_{HH} = 19.0 Hz, =*C*H-Si), 7.28-7.44 (m, 34H, -C_6H_5 and -C_6H_4-), 7.51 (s, 13H, =*C*H and -C_6H_5);
^{13}C NMR ($CDCl_3$, δ, ppm): 117.32, 117.39, 117.46, 117.54, 118.27, 118.35, 118.42, 127.07, 128.29, 128.75, 129.09, 147.93, 149.37, 149.41; **^{29}Si NMR** ($CDCl_3$, δ, ppm): -78.14, -78.27, -78.58;
IR (ATR, cm^{-1}): 3058.00, 3022.87 (C-H phenyl), 2961.29, 2925.96 (C-H), 1604.12 (C=C), 1574.21 (C=C), 1493.44 (C=C), 1447.37 (C=C), 1334.13, 1290.43, 1260.25 (Si-C), 1220.22, 1197.52, 1075.36 (Si-O), 988.88, 849.19, 815.55, 728.36, 686.53, 552.72, 467.04; **MALDI-TOF MS (m/z, $[M+Na]^+$):** found: 1264.22 (33), 1299.19 (57), 1333.15 (10); calculated respectively for: $C_{64}H_{56}O_{12}Si_8Na$ (4a$_8$): 1264.81, $C_{64}H_{55}ClO_{12}Si_8Na$ (4a$_7$c): 1299.25, $C_{64}H_{54}Cl_2O_{12}Si_8Na$ (4a$_6$c$_2$): 1333.70.

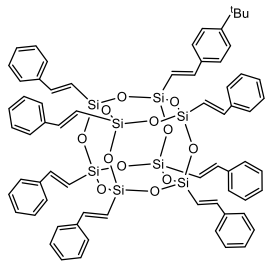

4a~7d

White solid

^{1}H NMR (CDCl$_3$, δ, ppm): 1.32 (s, 9H, -C(CH_3)$_3$), overlapping doublets: 6.26 and 6.32 (d, J_{HH}= 19.4 Hz and d, J_{HH}= 19.2 Hz, 8H, =C*H*-Si), 7.30-7.45 (m, 35H, -C$_6$*H*$_5$ and -C$_6$*H*$_4$-), 7.49 (d, 12H, J_{HH} = 7.4 Hz, -C$_6$*H*$_5$ and =C*H*); **^{13}C NMR** (CDCl$_3$, δ, ppm): 31.41 (C(*C*H$_3$)$_3$), 34.85 (*C*(CH$_3$)$_3$), 117.60, 125.65, 126.84, 127.10, 128.73, 129.07, 137.53, 149.34; **^{29}Si NMR** (CDCl$_3$, δ, ppm): -78.19, -78.32, -78.63; **IR** (ATR, cm^{-1}): 3057.54, 3023.07 (C-H phenyl), 2961.30, 2866.51 (C-H), 1719.62, 1604.34 (C=C), 1574.27 (C=C), 1494.22 (C=C), 1447.37 (C=C), 1410.22, 1290.22, 1259.67 (Si-C), 1220.61, 1197.85, 1076.09 (Si-O), 989.37, 848.28, 816.20, 728.63, 686.73, 549.86, 465.53; **MALDI-TOF MS (m/z, [M+Na]$^+$):** found: 1263.22 (10), 1319.29 (32), 1376.36 (34), 1433.43 (17), 1489.49 (5), 1544.55 (1); calculated respectively for: $C_{64}H_{56}O_{12}Si_8Na$ (3a$_8$): 1264.81, $C_{68}H_{64}O_{12}Si_8Na$ (4a$_7$d): 1320.91, $C_{72}H_{72}O_{12}Si_8Na$ (4a$_6$d$_2$): 1377.02, $C_{76}H_{80}O_{12}Si_8Na$ (4a$_5$d$_3$): 1433.13, $C_{80}H_{88}O_{12}Si_8Na$ (4a$_4$d$_4$): 1489.23, $C_{84}H_{96}O_{12}Si_8Na$ (4a$_3$d$_5$): 1545.34.

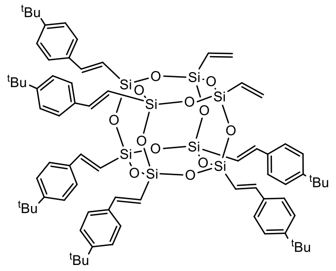

3d~6

White solid

^{1}H NMR (CDCl$_3$, δ, ppm): 1.32 (s, 54H, C(CH_3)$_3$), 5.96-6.30 (m, 10H, =C*H* and =C*H*-Si), 7.28-7.56 (m, 32H, -C$_6$*H*$_4$- and =C*H*); **^{13}C NMR** (CDCl$_3$, δ, ppm): 31.41 (C(*C*H$_3$)$_3$), 34.84 (*C*(CH$_3$)$_3$), 116.78, 125.62, 126.14, 126.83, 134.96, 148.97; **^{29}Si NMR** (CDCl$_3$, δ, ppm): -78.30, -78.34, -80.06; **IR** (ATR, cm^{-1}): 2960.64 (C-H), 2903.14 (C-H), 2866.83 (C-H), 1700.28, 1608.36 (C=C), 1561.53 (C=C), 1512.53, 1460.20 (C=C), 1409.51 (C=C), 1363.57, 1260.85 (Si-C), 1206.68, 1086.60 (Si-O), 989.22, 840.83, 795.92, 840.83, 795.92, 724.06, 594.89, 529.10, 475.16; **MALDI-TOF MS (m/z, [M+Na]$^+$):** found: 1316.47 (4), 1448.58 (48), 1580.68 (26), 1712.80 (22); calculated respectively for: $C_{66}H_{84}O_{12}Si_8Na$ (3d$_5$): 1317.85, $C_{76}H_{96}O_{12}Si_8Na$ (3d$_6$): 1449.24, $C_{86}H_{108}O_{12}Si_8Na$ (3d$_7$): 1581.45, $C_{96}H_{120}O_{12}Si_8Na$ (3d$_8$): 1713.65.

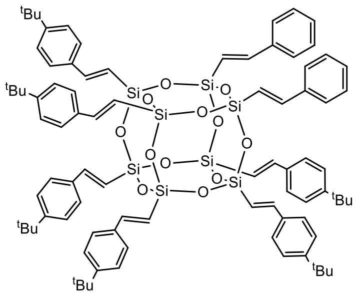

4d~6a~2

White solid

^{1}H NMR (CDCl$_3$, δ, ppm): 1.32 (s, 54H, C(CH_3)$_3$), overlapping doublets: 6.25 and 6.30 (d, J_{HH}= 19.3 Hz and d, J_{HH}= 20.5 Hz, 8H, =C*H*-Si), 7.31-7.39 (m, 26H, -C$_6$*H*$_4$- and -C$_6$*H*$_5$), 7.43 (d, 10H, J_{HH} = 8.2 Hz, -C$_6$*H*$_4$- and =C*H*), 7.48-7.52 (m, 6H, -C$_6$*H*$_4$- and =C*H*);
^{13}C NMR (CDCl$_3$, δ, ppm): 31.41 (C(*C*H$_3$)$_3$), 34.84 (*C*(CH$_3$)$_3$), 116.70, 117.75, 125.63, 126.66, 126.84, 127.11, 127.77, 128.70, 128.83, 134.93, 137.48, 137.59, 149.03, 149.21, 152.19; **^{29}Si NMR** (CDCl$_3$, δ, ppm): -78.17, -78.37; **IR** (ATR, cm^{-1}): 3024.14 (C-H phenyl), 2961.09 (C-H), 2866.51 (C-H), 1606.72 (C=C), 1512.39 (C=C), 1447.64 (C=C), 1410.11 (C=C), 1363.49, 1291.14, 1268.30 (Si-C), 1199.17, 1083.33 (Si-O), 989.45, 843.91, 796.31, 731.39, 688.48, 547.93, 473.79; **MALDI-TOF MS (m/z, [M+Na]$^+$):** found: 1487.54 (10), 1543.60 (32), 1600.68 (37), 1657.74 (18), 1713.81 (3); calculated respectively for: $C_{80}H_{88}O_{12}Si_8Na$ (4d$_4$a$_4$): 1487.4328, $C_{84}H_{96}O_{12}Si_8Na$ (4d$_5$a$_3$): 1545.34, $C_{88}H_{104}O_{12}Si_8Na$ (4d$_6$a$_2$): 1601.44, $C_{92}H_{112}O_{12}Si_8Na$ (4d$_7$a): 1657.55, $C_{96}H_{120}O_{12}Si_8Na$ (3d$_8$): 1713.66.

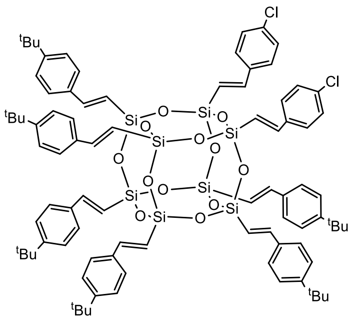

4d~6c~2

White solid

^{1}H NMR (CDCl$_3$, δ, ppm): 1.32 (s, 54H, -C(CH_3)$_3$), overlapping doublets: 6.24 and 6.26 (d, J_{HH}= 19.2 Hz and d, J_{HH}= 19.2 Hz, 6H,), 7.02- 7.07 (m, 2H, =C*H*-Si), 7.31-7.44 (m, 40H, -C$_6$*H*$_4$- and =C*H*);
^{13}C NMR (CDCl$_3$, δ, ppm): 31.40 (C(*C*H$_3$)$_3$), 34.85 (*C*(CH$_3$)$_3$), 125.66, 125.82, 126.29, 126.44, 126.74, 126.82, 127.71, 127.84, 128.13, 128.28, 128.94, 129.06, 129.28, 134.83, 135.65, 147.83, 149.10; **^{29}Si NMR** (CDCl$_3$, δ, ppm): -78.12, -78.60; **IR** (ATR, cm^{-1}): 2961.19 (C-H phenyl), 2902.55 (C-H), 2866.43 (C-H), 1607.07 (C=C), 1562.95 (C=C), 1489.64 (C=C), 1402.76 (C=C), 1363.40, 1293.09, 1268.45 (Si-C), 1198.87, 1086.79 (Si-O), 1011.43, 988.79, 841.95, 789.41, 724.08, 592.17, 529.17, 479.34; **MALDI-TOF MS (m/z, [M+Na]$^+$):** found: 1648.46 (18), 1669.56 (42), 1691.66 (40); calculated respectively for: $C_{84}H_{93}Cl_3NaO_{12}Si_8$ (4d$_5$c$_3$) 1648.66 $C_{88}H_{102}Cl_2O_{12}Si_8Na$ (4d$_6$c$_2$): 1670.33, $C_{92}H_{111}ClO_{12}Si_8Na$ (4d$_7$c$_1$) 1689.58

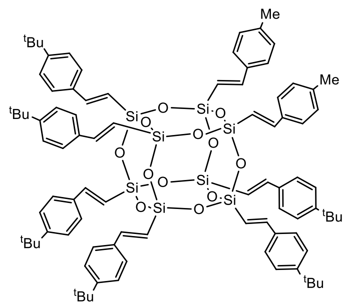

4d~6b~2

White solid

^{1}H NMR (CDCl$_3$, δ, ppm): 1.32 (s, 54H, -C(CH_3)$_3$), 2.35 (s, 6H, -CH_3), overlapping doublets: 6.24 and 6.25 (d, J_{HH}= 19.1 Hz and d,
J_{HH}= 19.2 Hz, 8H, =C*H*-Si), 7.15 (d, 4H, J_{HH}= 7.8 Hz, -C$_6$*H*$_4$-),
7.33-7.47 (m, 36H, -C$_6$*H*$_4$- and =C*H*); **^{13}C NMR** (CDCl$_3$, δ, ppm): 21.46 (*C*H$_3$), 31.41 (C(*C*H$_3$)$_3$), 34.83 (*C*(CH$_3$)$_3$), 116.43, 116.77, 125.62, 126.83, 127.05, 129.40, 134.96, 148.97, 149.11, 152.15; **^{29}Si NMR** (CDCl$_3$, δ, ppm): -78.16, -78.20; **IR** (ATR, cm^{-1}): 3083.30 (C-H phenyl), 2960.96 (C-H), 2866.58 (C-H), 1720.29, 1607.31 (C=C), 1561.74 (C=C), 1511.15 (C=C), 1459.29 (C=C), 1409.84 (C=C), 1363.43, 1290.53 (Si-C), 1268.39, 1200.14, 1183.22, 1077.17 (Si-O), 988.56, 838.39, 796.20, 724.08, 598.56, 523.75, 465.79; **MALDI-TOF MS (m/z, [M+Na]$^+$):** found: 1503.54 (8), 1544.58 (27), 1586.63 (39), 1628.68 (21), 1713.77 (4); calculated respectively for: $C_{81}H_{90}O_{12}Si_8Na$ (4d$_3$c$_5$): 1503.26, $C_{84}H_{96}O_{12}Si_8Na$ (4d$_4$c$_4$): 1545.34, $C_{87}H_{102}O_{12}Si_8Na$ (4d$_5$c$_3$) 1587.41, $C_{90}H_{108}O_{12}Si_8Na$ (4d$_6$c$_2$): 1629.49, $C_{96}H_{120}O_{12}Si_8Na$ (4d$_7$c): 1713.66

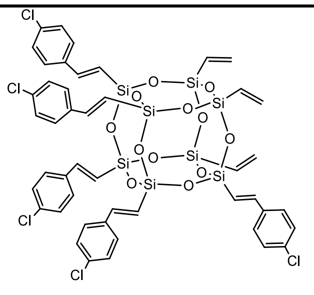

3c$_{\sim5}$

White solid

^{1}H NMR ($CDCl_3$, δ, ppm): 5.92-6.28 (m, 14H, =C*H*-Si and =C*H_2*), 7.19-7.25 (m, 3H, -C_6*H_4*-), 7.27-7.34 (m, 12H, -C_6*H_4*-), 7.37-7.42 (m, 10H, -C_6*H_4*- and =C*H*); **^{13}C NMR** ($CDCl_3$, δ, ppm): 117.99, 128.24, 129.02, 135.03, 135.82, 137.47, 148.10; **^{29}Si NMR** ($CDCl_3$, δ, ppm): -78.44, -78.49, -80.20; **IR** (ATR, cm^{-1}): 3061.73 (C-H phenyl), 2959.59 (C-H), 1604.54 (C=C), 1488.85 (C=C), 1402.87 (C=C), 1277.67 (Si-C), 1196.30, 1072.72 (Si-O), 1010.13, 851.95, 785.02, 577.67, 477.25; **MALDI-TOF MS (m/z, [M+Na]$^+$):** found: 1208.40 (64), 1319.60 (36); calculated respectively for: $C_{46}H_{39}Cl_5O_{12}Si_8Na$ (3c$_5$): 1208.74, $C_{52}H_{42}Cl_6O_{12}Si_8Na$ (3c$_6$): 1319.28.

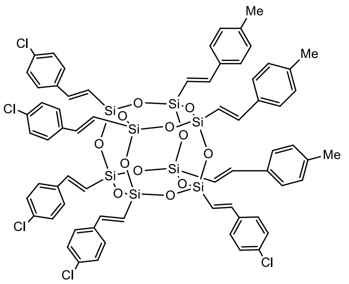

4c$_{\sim5}$**b**$_{\sim3}$

White solid

^{1}H NMR ($CDCl_3$, δ, ppm): 2.36 (s, 9H, -C*H_3*), overlapping doublets: 6.23 and 6.26 (d, J_{HH} = 19.1 Hz and d, J_{HH} = 19.2 Hz, 8H, =C*H*-Si), 7.16 (d, 8H, J_{HH} = 7.2 Hz, -C_6*H_4*-), 7.31 (d, 15H, J_{HH} = 7.0 Hz, -C_6*H_4*-), 7.35 – 7.41 (m, 17H, -C_6*H_4*- and =C*H*); **^{13}C NMR** ($CDCl_3$, δ, ppm): 21.47 (CH_3), 127.02, 128.26, 128.99, 129.48, 148.02; **^{29}Si NMR** ($CDCl_3$, δ, ppm): -78.19, -78.52; **IR** (ATR, cm^{-1}): 2999.66 (C-H phenyl), 2960.16 (C-H), 2921.15 (C-H), 1606.35 (C=C), 1566.13 (C=C), 1510.32 (C=C), 1489.11 (C=C), 1402.53, 1279.95 (Si-C), 1260.19, 1222.82, 1197.23, 1079.40 (Si-O), 986.94, 854.87, 781.85, 672.13, 589.66, 525.08, 481.06; **MALDI-TOF MS (m/z, [M+Na]$^+$):** found: 1417.27 (10), 1437.22 (23), 1459.01 (36), 1479.80 (21), 1499.90 (9); calculated respectively for: $C_{70}H_{66}Cl_2NaO_{12}Si_8$ (4c$_2$b$_6$): 1417.85, $C_{69}H_{63}Cl_3NaO_{12}Si_8$ (4c$_3$b$_5$): 1438.26, $C_{68}H_{60}Cl_4O_{12}Si_8Na$ (4c$_4$b$_4$): 1458.69, $C_{67}H_{57}Cl_5O_{12}Si_8Na$ (4c$_5$b$_3$): 1479.11, $C_{66}H_{54}Cl_6O_{12}Si_8Na$ (4c$_6$b$_2$): 1499.53.

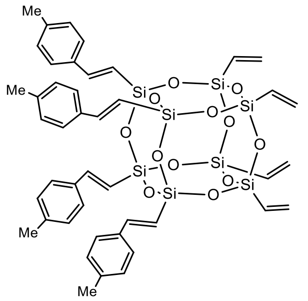

3b$_{\sim4}$

White solid

^{1}H NMR ($CDCl_3$, δ, ppm): 2.35 (s, 12H, -C*H_3*), 5.92-6.26 (m, 16H, =C*H_2* and =C*H*-Si), 7.15 (d, 8H, J_{HH} = 7.5 Hz, -C_6*H_4*-), 7.19-7.25 (m, 2H, -C_6*H_4*-), 7.27-7.32 (m, 2H, -C_6*H_4*-), 7.37 (d, 8H, J_{HH} = 7.9 Hz,
-C_6*H_4*- and =C*H*); **^{13}C NMR** ($CDCl_3$, δ, ppm): 21.46 (CH_3), 116.21, 127.01, 129.41, 134.85, 137.14, 139.07, 149.13; **^{29}Si NMR** ($CDCl_3$, δ, ppm): -78.44, -78.49, -80.20; **IR** (ATR, cm^{-1}): 3024.71 (C-H phenyl), 2959.69 (C-H), 1604.38 (C=C), 1511.10 (C=C), 1407.17 (C=C), 1197.87, 1073.01 (Si-O), 987.58 (C-H phenyl), 831.79, 779.55, 573.22, 527.78, 491.20, 460.79; **MALDI-TOF MS (m/z, [M+Na]$^+$):** found: 925.10 (11), 1015.16 (33), 1105.21 (26), 1195.25 (22), 1286.30 (8); calculated respectively for: $C_{37}H_{42}O_{12}Si_8Na$ (3b$_3$): 926.41, $C_{44}H_{48}O_{12}Si_8Na$ (3b$_4$): 1016.53, $C_{51}H_{54}O_{12}Si_8Na$ (3b$_5$): 1106.62, $C_{58}H_{60}O_{12}Si_8Na$ (3b$_6$): 1196.77, $C_{65}H_{66}O_{12}Si_8Na$ (3b$_7$): 1286.89.

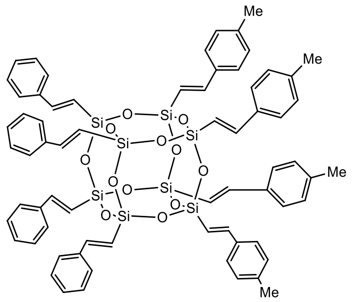

4b$_{\sim4}$**a**$_{\sim4}$

White solid

^{1}H NMR ($CDCl_3$, δ, ppm): 2.36 (s, 12H, -C*H_3*), 6.29 (t, 8H, J_{HH} = 19.6 Hz, =C*H*-Si), 7.16 (d, 8H, J_{HH} = 7.7 Hz, -C_6*H_4*- and -C_6*H_5*), 7.30-7.43 (m, 29H, -C_6*H_4*- and -C_6*H_5*), 7.50 (d, 7H, =C*H* and -C_6*H_5*); **^{13}C NMR** ($CDCl_3$, δ, ppm): 21.46 (CH_3), 116.27 (=CH), 117.65 (=CH), 127.04, 127.09, 128.71, 129.03, 129.42, 137.54, 149.26; **^{29}Si NMR** ($CDCl_3$, δ, ppm): -78.03, -78.05, -78.30; **IR** (ATR, cm^{-1}): 3022.30 (C-H phenyl), 3000.05 (C-H phenyl), 2921.14 (C-H), 2853.24 (C-H), 1604.95 (C=C), 1574.04 (C=C), 1510.06 (C=C), 1494.38 (C=C), 1447.41, 1289.76 (Si-C), 1197.42, 1068.72 (Si-O), 987.84 (C-H phenyl), 851.71, 781.83, 729.07, 687.31, 523.56, 462.48; **MALDI-TOF MS (m/z, [M+Na]$^+$):** found: 1292.26 (8), 1306.27 (20), 1320.29 (30), 1334.31 (25), 1348.32 (13), 1363.34 (4); calculated respectively for: $C_{66}H_{60}O_{12}Si_8Na$ (4b$_2$a$_6$): 1291.2137, $C_{67}H_{62}O_{12}Si_8Na$ (4b$_3$a$_5$): 1305.23, $C_{68}H_{64}O_{12}Si_8Na$ (4b$_4$a$_4$): 1320.92, $C_{69}H_{66}O_{12}Si_8Na$ (4b$_5$a$_3$): 1333.26, $C_{70}H_{68}O_{12}Si_8Na$ (4b$_6$a$_2$): 1347.28, $C_{71}H_{70}O_{12}Si_8Na$ (4b$_7$a): 1361.29.

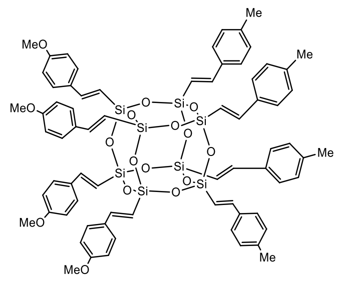

4b$_{\sim4}$**e**$_{\sim4}$

White solid

^{1}H NMR ($CDCl_3$, δ, ppm): 2.24 (s, 12H,-C*H_3*), 3.71 (s, 12H, -OC*H_3*), 6.03 (d, 4H, J_{HH} = 19.1 Hz, =C*H*-Si), 6.13 (d, 4H, J_{HH} = 19.2 Hz,
=C*H*-Si), 6.76 (d, 8H, J_{HH} = 8.6 Hz, -C_6*H_4*-), 7.04 (d, 8H, J_{HH} = 7.9 Hz,
-C_6*H_4*-), 7.14-7.24 (m, 12H, -C_6*H_4*- and =C*H*), 7.28-7.34 (m, 12H,
-C_6*H_4*- and =C*H*); **^{13}C NMR** ($CDCl_3$, δ, ppm): 21.46 (CH_3), 55.45 (OCH_3), 114.07 (=CH), 114.92 (=CH), 116.46 (=CH), 127.03, 128.45, 129.40, 134.94, 138.98, 148.63, 149.08, 160.35; **^{29}Si NMR** ($CDCl_3$, δ, ppm): -77.97, -78.16; **IR** (ATR, cm^{-1}): 2999.56 (C-H phenyl), 2954.07 (C-H), 2930.67 (C-H), 2836.30 (C-H), 1603.99 (C=C), 1570.29 (C=C), 1508.53 (C=C), 1462.87 (C=C), 1419.05 (C=C), 1333.21, 1296.66, 1252.15 (Si-C), 1198.38, 1077.76 (Si-O), 985.54, 832.80, 781.23, 602.44, 524.49, 488.08; **MALDI-TOF MS (m/z, [M+Na]$^+$):** found: 1409.32 (7), 1424.32 (21), 1440.32 (35), 1456.31 (34), 1472.29 (2); calculated respectively for: $C_{72}H_{72}O_{14}Si_8Na$ (4b$_2$d$_6$):_ 1407.2974, $C_{72}H_{72}O_{15}Si_8Na$ (4b$_3$d$_5$): 1423.29, $C_{72}H_{72}O_{16}Si_8Na$ (4b$_4$d$_4$): 1441.01, $C_{72}H_{72}O_{17}Si_8Na$ (4b$_5$d$_3$): 1455.28, $C_{72}H_{72}O_{18}Si_8Na$ (4b$_6$d$_2$): 1471.28.

2. Analytical Data of Silylative Coupling Products

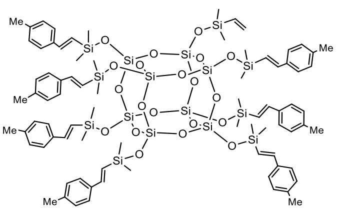

6b$_{-7}$

White solid

^{1}H NMR ($CDCl_3$, δ, ppm): 0.24 (s, 48H, Si(CH_3)$_2$), 2.28 (s, 15H, -CH_3), overlapping doublets: 6.30 and 6.31 (d, J_{HH} = 19.3 Hz, and d, J_{HH} = 19.3, 9H, =CH_2 and =CH-Si), 6.92 (d, 8H, J_{HH} = 19.3 Hz, =CH and =CH_2), 7.01-7.09 (m, 18H, -C_6H_4-), 7.27-7.31 (m, 10H, -C_6H_4-); ^{13}C NMR ($CDCl_3$, δ, ppm): 0.40 (Si(CH_3)$_2$), 21.35 (CH_3), 125.73, 126.72, 129.26, 135.50, 138.08, 144.98; ^{29}Si NMR ($CDCl_3$, δ, ppm): 1.68, -108.80; IR (ATR, cm^{-1}): 3024.19 (C-H phenyl), 2956.72 (C-H), 2919.97 (C-H), 1606.07 (C=C), 1566.67 (C=C), 1509.94 (C=C), 1408.62 (C=C), 1328.81, 1251.80 (Si-C), 1177.25, 1066.56 (Si-O), 987.23, 827.46, 794.61, 644.28, 549.57, 514.03; MALDI-TOF MS (m/z, [M+Na]$^+$): found: 1879.44 (64), 1969.48 (36); calculated respectively for: $C_{81}H_{114}O_{20}Si_{16}Na$ (6b$_7$): 1880.13, $C_{88}H_{120}O_{20}Si_{16}Na$ (6b$_8$): 1970.25.

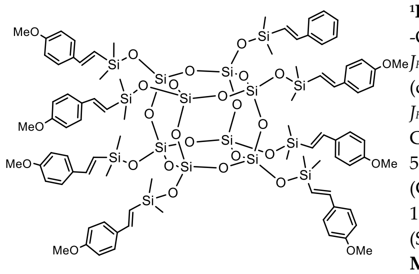

7e$_{-7}$**a**$_{-1}$

White solid

^{1}H NMR ($CDCl_3$, δ, ppm): 0.24 (s, 48H, Si(CH_3)$_2$), 3.73 (s, 21H, -OCH_3), 6.20 (d, 7H, J_{HH} = 19.2 Hz, =CH-Si), 6.37 (d, 1H, J_{HH} = 19.2 Hz, =CH-Si), 6.76 (d, 14H, J_{HH} = 8.6 Hz, -C_6H_4-), overlapping doublets: 6.90 and 6.95 (d, J_{HH} = 19.3 Hz and d, J_{HH} = 21.6 Hz, 8H, =CH), 7.19 – 7.25 (m, 4H, -C_6H_4-), 7.31 (d, 11H, J_{HH} = 8.6 Hz, -C_6H_4- and -C_6H_5), 7.38 (d, 4H, J_{HH} = 7.2 Hz, -C_6H_4- and -C_6H_5); **^{13}C NMR** ($CDCl_3$, δ, ppm): 0.46 (Si(CH_3)$_2$), 55.34 (OCH_3), 113.94, 124.23, 126.80, 128.07, 128.56, 131.14, 138.15, 144.51, 159.82; **^{29}Si NMR** ($CDCl_3$, δ, ppm): 1.69, 1.61, -108.80**; IR** (ATR, cm^{-1}): 2956.31 (C-H phenyl), 2836.45 (C-H), 1604.53 (C=C), 1571.19 (C=C), 1508.99 (C=C), 1464.29 (C=C), 1441.19, 1417.76, 1332.54, 1296.24 (Si-C), 1250.54, 1170.44, 1065.50 (Si-O), 987.70, 829.38, 794.45, 644.90, 543.71; **MALDI-TOF MS (m/z, [M+Na]$^+$):** found: 1977.45 (3), 2007.46 (11), 2037.47 (28), 2067.49 (39), 2097.50 (19); calculated respectively for: $C_{84}H_{112}O_{24}Si_{16}Na$ (7e$_4$a$_4$): 1975.37, $C_{85}H_{114}O_{25}Si_{16}Na$ (7e$_5$a$_3$): 2005.38, $C_{86}H_{116}O_{26}Si_{16}Na$ (7e$_6$a$_2$): 2035.40, $C_{87}H_{118}O_{27}Si_{16}Na$ (7e$_7$a$_1$): 2065.41, $C_{88}H_{120}O_{28}Si_{16}Na$ (6e$_8$): 2095.41.

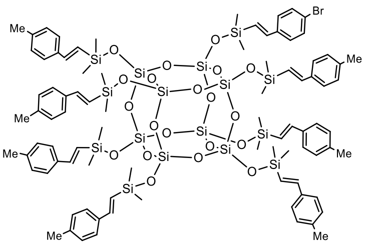

7b$_{-7}$**f**$_{-1}$

White solid

^{1}H NMR ($CDCl_3$, δ, ppm): 0.24 (s, 48H, Si(CH_3)$_2$), 2.29 (s, 12H, -OCH_3), 6.30 (d, J_{HH} = 19.2 Hz, 8H, =CH-Si), 6.92 (m, 9H, -C_6H_4-), 7.04 (d, 14H, J_{HH} = 7.7 Hz, -C_6H_4-), 7.19 (d, 5H, J_{HH} = 8.3 Hz, -C_6H_4-), 7.28 (s, 12H, =CH and -C_6H_4-); **^{13}C NMR** ($CDCl_3$, δ, ppm): 0.40 (Si(CH_3)$_2$), 21.35 (OCH_3), 125.72, 126.72, 128.27, 129.26, 131.63, 135.49, 138.08, 145.01; **^{29}Si NMR** ($CDCl_3$, δ, ppm): 1.70, 1.65, -108.79; **IR** (ATR, cm^{-1}): 3023.98 (C-H phenyl), 2956.98 (C-H), 2920.55 (C-H), 1719.84 (C=C), 1605.98 (C=C), 1566.15 (C=C), 1509.97, 1408.54, 1251.88 (Si-C), 1065.33 (Si-O), 987.05 (C-H phenyl), 827.23, 795.29, 644.95, 550.18, 514.21; **MALDI-TOF MS (m/z, [M+Na]$^+$):** found: 1969.49 (48), 2034.38 (40), 2099.28 (12); calculated respectively for: $C_{88}H_{120}O_{20}Si_{16}Na$ (7b$_8$): 1970.25, $C_{87}H_{117}BrO_{20}Si_{16}Na$ (7b$_7$f$_1$): 2035.12, $C_{86}H_{114}Br_2O_{20}Si_{16}Na$ (7b$_6$f$_2$): 2099.98.

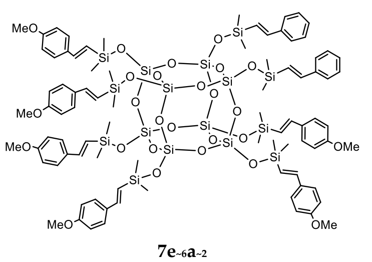

7e$_{-6}$**a**$_{-2}$

White solid

^{1}H NMR ($CDCl_3$, δ, ppm): 0.24 (s, 48H, Si(CH_3)$_2$), 3.74 (s, 18H, -OCH_3), 6.20 (dd, J_{HH} = 19.3, 2.6 Hz, 6H, =CH-Si), 6.33 (d, J_{HH} = 19.2 Hz, 2H, =CH-Si), 6.77 (d, 14H, J_{HH} = 8.4 Hz, -C_6H_4-), overlapping doublets: 6.87 and 6.91 (d, J_{HH} = 19.0 Hz and d, J_{HH} = 19.2 Hz, 8H, =CH), 7.20 (d, 3H, J_{HH} = 8.2 Hz, -C_6H_4- and -C_6H_5), 7.31 (d, 17H, J_{HH} = 6.8 Hz, -C_6H_4- and -C_6H_5); **^{13}C NMR** ($CDCl_3$, δ, ppm): 0.46 (Si(CH_3)$_2$), 55.35 (OCH_3), 113.95, 124.18, 128.06, 128.28, 131.09, 131.13, 131.65, 137.06, 143.71, 143.76, 144.56, 159.84; **^{29}Si NMR** ($CDCl_3$, δ, ppm): 1.73, 1.68, -108.81; **IR** (ATR, cm^{-1}): 2955.98 (C-H phenyl), 2932.59 (C-H), 2836.24 (C-H), 1604.46 (C=C), 1570.95 (C=C), 1509.02, 1464.16, 1250.47 (Si-C), 1170.49, 1065.60 (Si-O), 988.06 (C-H phenyl), 830.50, 791.14, 555.30; **MALDI-TOF MS (m/z, [M+Na]$^+$):** found: 2003.58 (30), 2040.34 (49), 2063.16 (21); calculated respectively for: $C_{85}H_{114}O_{25}Si_{16}Na$ (7e$_5$a$_3$): 2005.39, $C_{86}H_{116}O_{26}Si_{16}Na$ (7e$_6$a$_2$): 2038.19, $C_{87}H_{118}O_{27}Si_{16}Na$ (7e$_7$a): 2065.41.

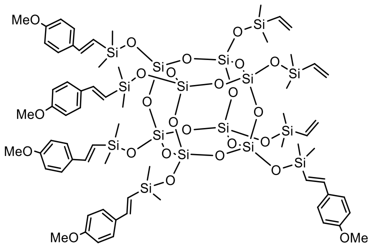

6e$_{-5}$

White solid

^{1}H NMR ($CDCl_3$, δ, ppm): 0.24 (s, 48H, Si(CH_3)$_2$), 3.74 (s, 15H, -OCH_3), 6.19 (dt, J_{HH} = 19.2, 3.5 Hz, 5H, =CH-Si), 6.31 (d, J_{HH} = 19.2, 2.6 Hz, 2H, =CH-Si), 6.77 (d, 9H, J_{HH} = 8.5 Hz, -C_6H_4-), overlapping doublets: 6.88 and 6.91 (dd, J_{HH} = 19.3, 1.8 Hz and dt, J_{HH} = 19.2, 2.1 Hz, 7H, =CH_2 and =CH-Si), 7.18 (d, 5H, J_{HH} = 8.3 Hz, -C_6H_4-), 7.27-7.33 (m, 11H, -C_6H_4-); **^{13}C NMR** ($CDCl_3$, δ, ppm): 0.15 (Si(CH_3)$_2$), 55.35 (OCH_3), 113.96, 124.14, 127.95, 128.05, 128.72, 143.71, 144.59, 159.88; **^{29}Si NMR** ($CDCl_3$, δ, ppm): 2.19, 0.43, -108.39; **IR** (ATR, cm^{-1}): 2956.51, 2902.35, 2835.73, 1604.19, 1570.79, 1508.66, 1488.40, 1464.29, 1417.51, 1330.35, 1296.21, 1250.45, 1169.94, 1065.01, 987.30, 829. 41, 789.49, 543.68; **MALDI-TOF MS (m/z, [M+Na]$^+$):** found: 1672.21 (28), 1780.23 (43), 1885.10 (21), 1990.59 (8); calculated respectively for: $C_{60}H_{96}O_{24}Si_{16}Na$ (6e$_4$): 1671.25, $C_{67}H_{102}O_{25}Si_{16}Na$ (6e$_5$): 1779.88, $C_{74}H_{108}O_{26}Si_{16}Na$ (6e$_6$): 1883.33, $C_{81}H_{114}O_{27}Si_{16}Na$ (6e$_7$): 1989.38.

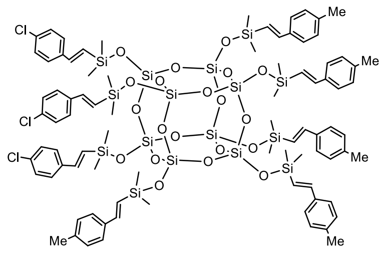<br>**7b$_{-5}$c$_{-3}$**	White solid<br><br>**^{1}H NMR** ($CDCl_3$, δ, ppm): 0.24 (s, 48H, Si(CH_3)$_2$), 2.29 (s, 15H, -CH_3), 6.27 – 6.34 (d, J_{HH} = 19.2 Hz, 8H, =CH-Si), 6.90 (t, 8H, J_{HH} = 18.4 Hz, -C_6H_4-), 7.05 (d, 14H, J_{HH} = 7.7 Hz, -C_6H_4-), 7.15-7.20 (m, 6H, -C_6H_4-), 7.25 (s, 6H, -C_6H_4-), 7.26-7.29 (m, 9H, =CH and -C_6H_4-); **^{13}C NMR** ($CDCl_3$, δ, ppm): 0.40 (Si(CH_3)$_2$), 21.36 (CH_3), 125.60, 126.71, 127.95, 128.72, 129.29, 133.93, 135.43, 138.19, 143.76, 145.05; **^{29}Si NMR** ($CDCl_3$, δ, ppm): 2.16, 0.41, -108.46; **IR** (ATR, cm^{-1}): 3023.39 (C-H phenyl), 2957.58 (C-H), 1606.20 (C=C), 1565.81 (C=C), 1509.99 (C=C), 1488.45 (C=C), 1401.37, 1252.05 (Si-C), 1064.82 (Si-O), 986.75 (C-H phenyl), 827.19, 794.65, 548.74, 514.49; **MALDI-TOF MS (m/z, [M+Na]$^+$):** found: 1969.49 (5), 1989.44 (23), 2010.39 (28), 2031.33 (32), 2051.28 (10), 2077.22 (2); calculated respectively for: $C_{88}H_{120}O_{20}Si_{16}Na$ (6b$_8$): 1970.24, $C_{87}H_{117}ClO_{20}Si_{16}Na$ (7b$_7$c): 1990.66, $C_{86}H_{114}Cl_2O_{20}Si_{16}Na$ (7b$_6$c$_2$): 2011.09, $C_{85}H_{111}Cl_3O_{20}Si_{16}Na$ (7b$_5$c$_3$): 2031.51, $C_{84}H_{108}Cl_4O_{20}Si_{16}Na$ (7b$_4$c$_4$): 2051.92, $C_{83}H_{105}Cl_5O_{20}Si_{16}Na$ (7b$_3$c$_5$): 2072.33.
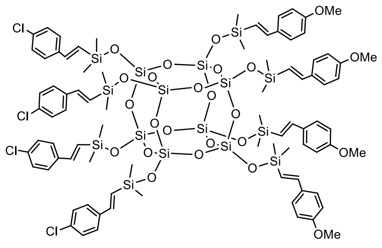<br>**7e$_{-4}$c$_{-4}$**	White solid<br><br>**^{1}H NMR** ($CDCl_3$, δ, ppm): 0.24 (s, 48H, Si(CH_3)$_2$), 3.74 (s, 12H, -OCH_3), 6.19 (dt, J_{HH} = 19.2, 3.5 Hz, 4H, =CH-Si), 6.31 (dt, J_{HH} = 19.2, 2.6 Hz, 4H, =CH-Si), 6.77 (d, J_{HH} = 8.5 Hz, 9H, -C_6H_4-), 6.85-6.93 (m, 8H, -C_6H_4-), 7.16-7.25 (m, 11H, -C_6H_4-), 7.27-7.35 (m, 12H, -C_6H_4- and =CH); **^{13}C NMR** ($CDCl_3$, δ, ppm): 0.45 (Si(CH_3)$_2$), 55.36 (OCH_3), 113.96, 124.11, 127.95, 128.05, 128.73, 131.07, 136.59, 143.76, 144.61, 159.89; **^{29}Si NMR** ($CDCl_3$, δ, ppm): 1.69, 1.64, -108.85; **IR** (ATR, cm^{-1}): 2957.75 (C-H phenyl), 2903.75, 2835.89, 1604.72, 1508.89, 1488.78, 1464.37, 1401.58, 1250.25 (Si-C), 1169.82, 1061.45 (Si-O), 986.12, 828.15, 789.31, 664.85, 543.83; **MALDI-TOF MS (m/z, [M+Na]$^+$):** found: 2006.36 (4), 2109.40 (96); calculated respectively for: $C_{85}H_{111}Cl_3O_{25}Si_{16}Na$ (7e$_5$c$_3$): 2107.26, $C_{84}H_{108}Cl_4O_{24}Si_{16}Na$ (7e$_4$c$_4$): 2111.22.

3. NMR Spectra of Isolated Products

3.1. NMR Spectra of Cross Metathesis Products

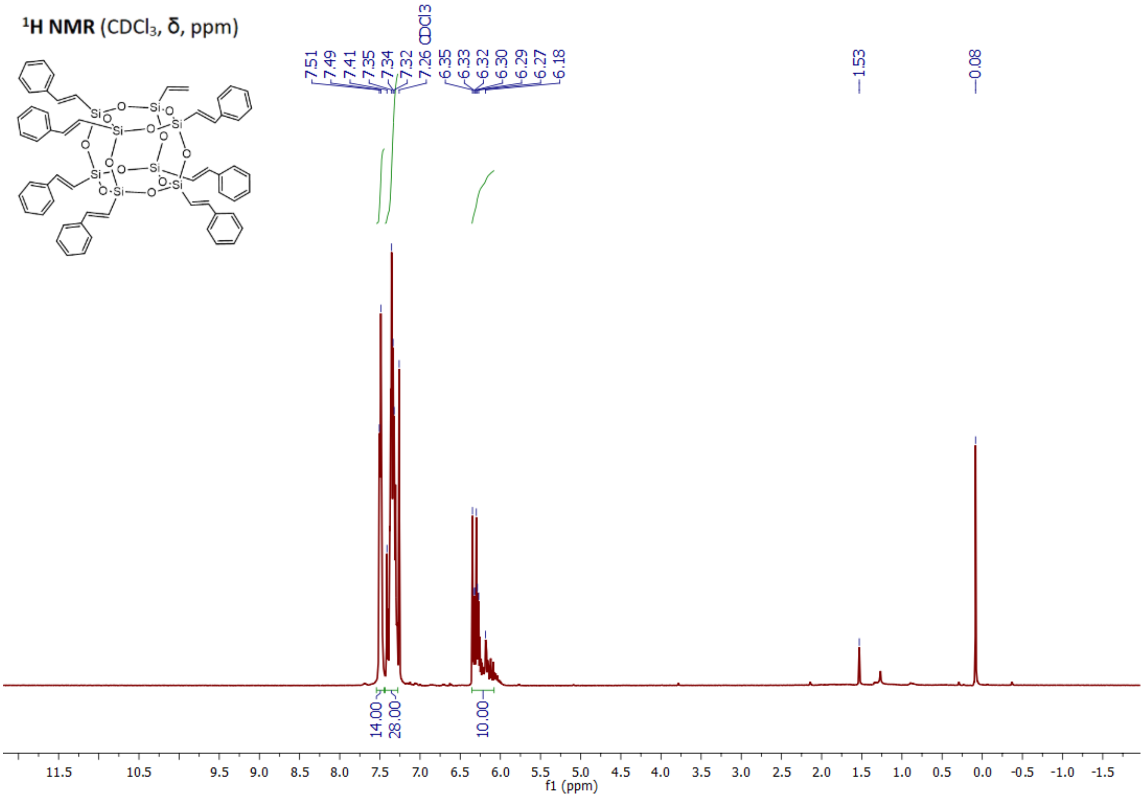

Figure S1. ^{1}H NMR spectrum of 3a$_7$.

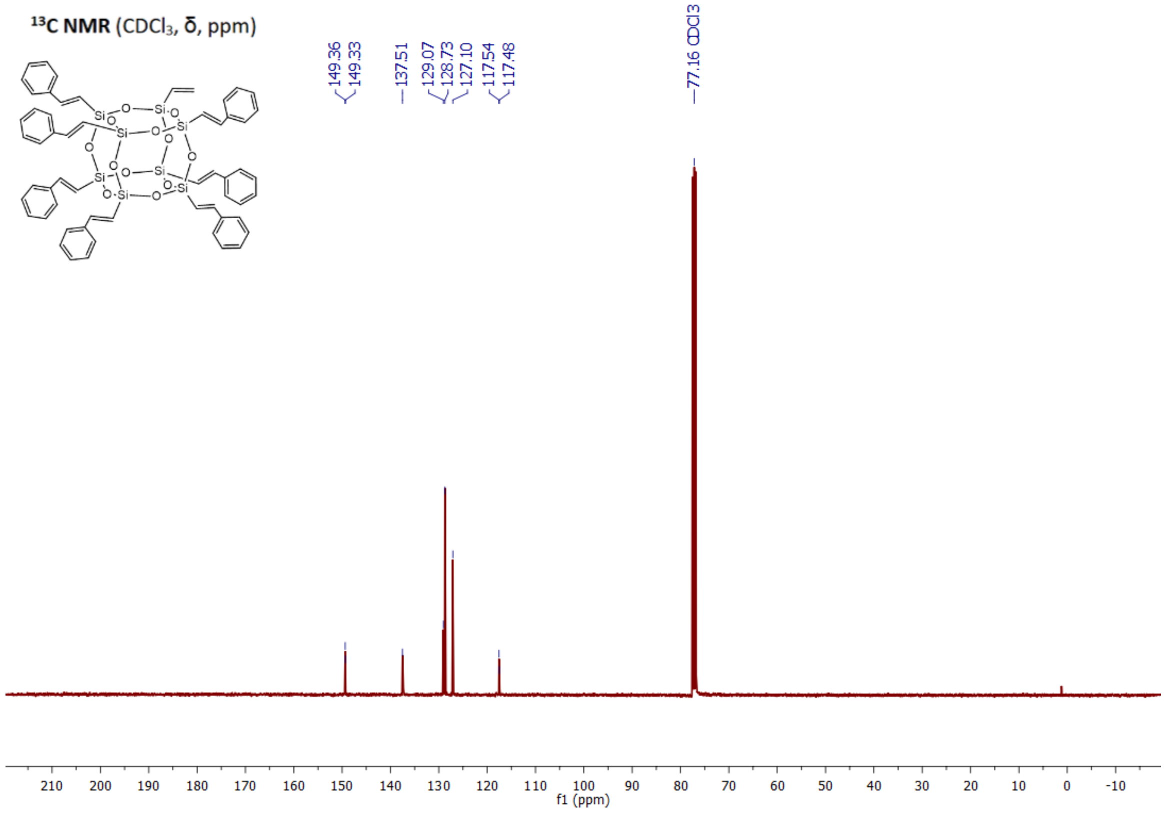

Figure S2. ^{13}C NMR spectrum of 3a7.

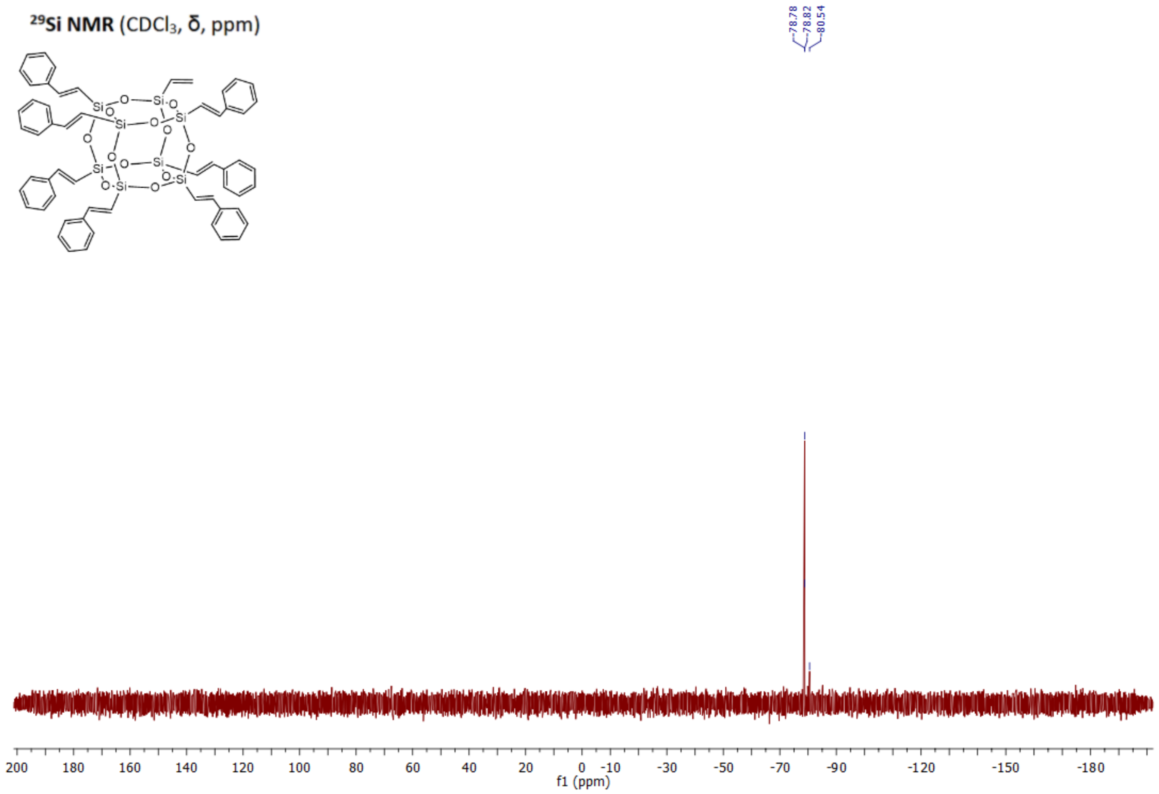

Figure S3. ^{29}Si NMR spectrum of 3a7.

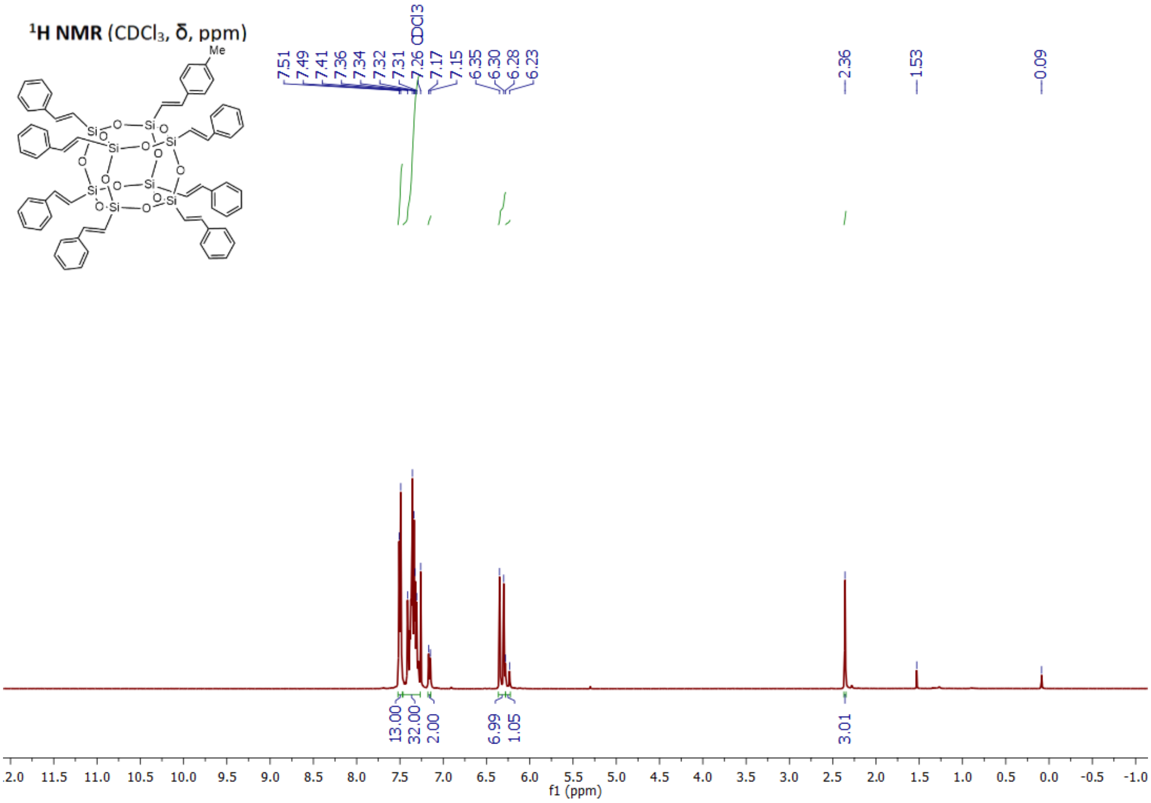

Figure S4. ^{1}H NMR spectrum of $4a_7b$.

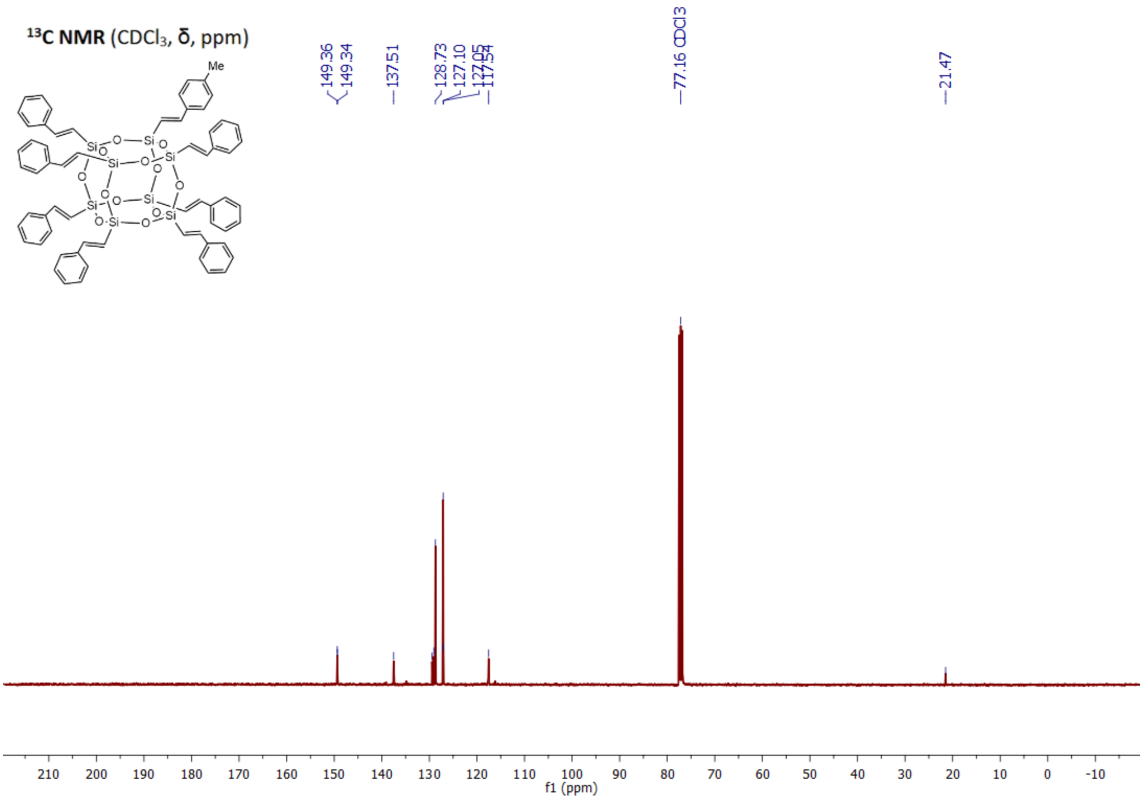

Figure S5. ^{13}C NMR spectrum of $4a_7b$.

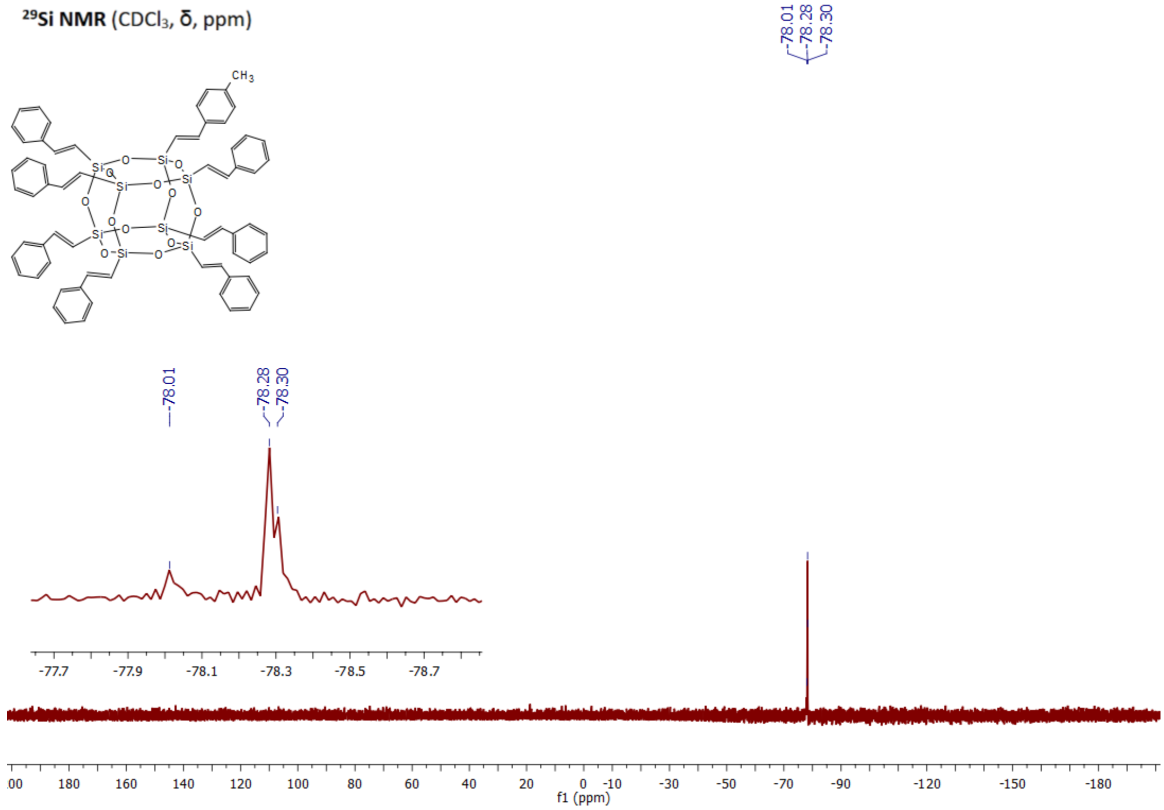

Figure S6. ^{29}Si NMR spectrum of 4a7b.

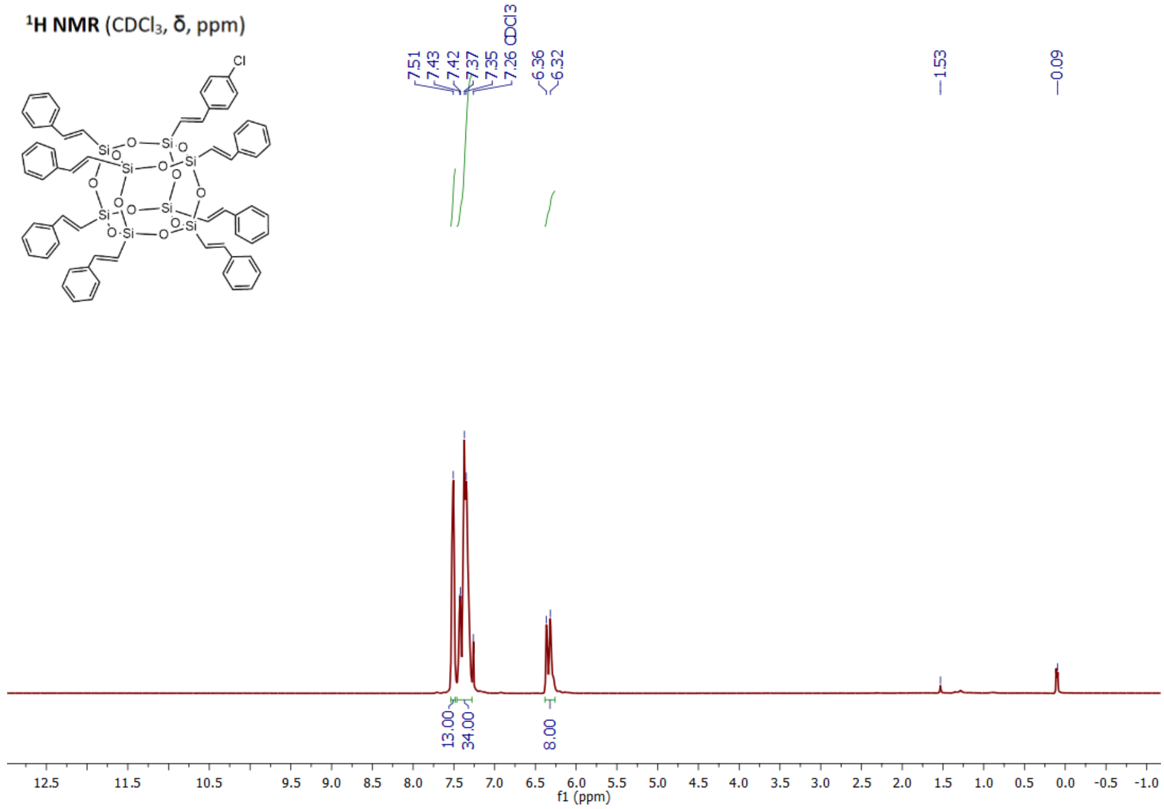

Figure S7. ^{1}H NMR spectrum of 4a7c.

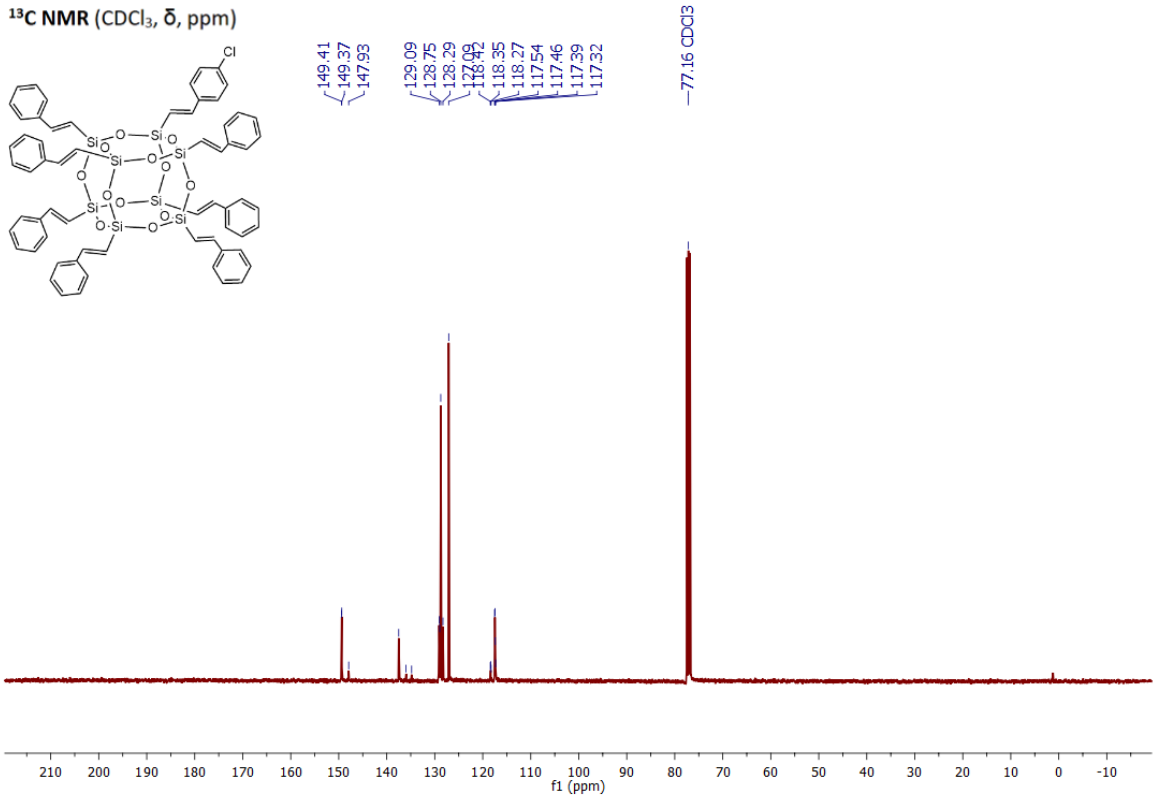

Figure S8. ^{13}C NMR spectrum of 4a7c.

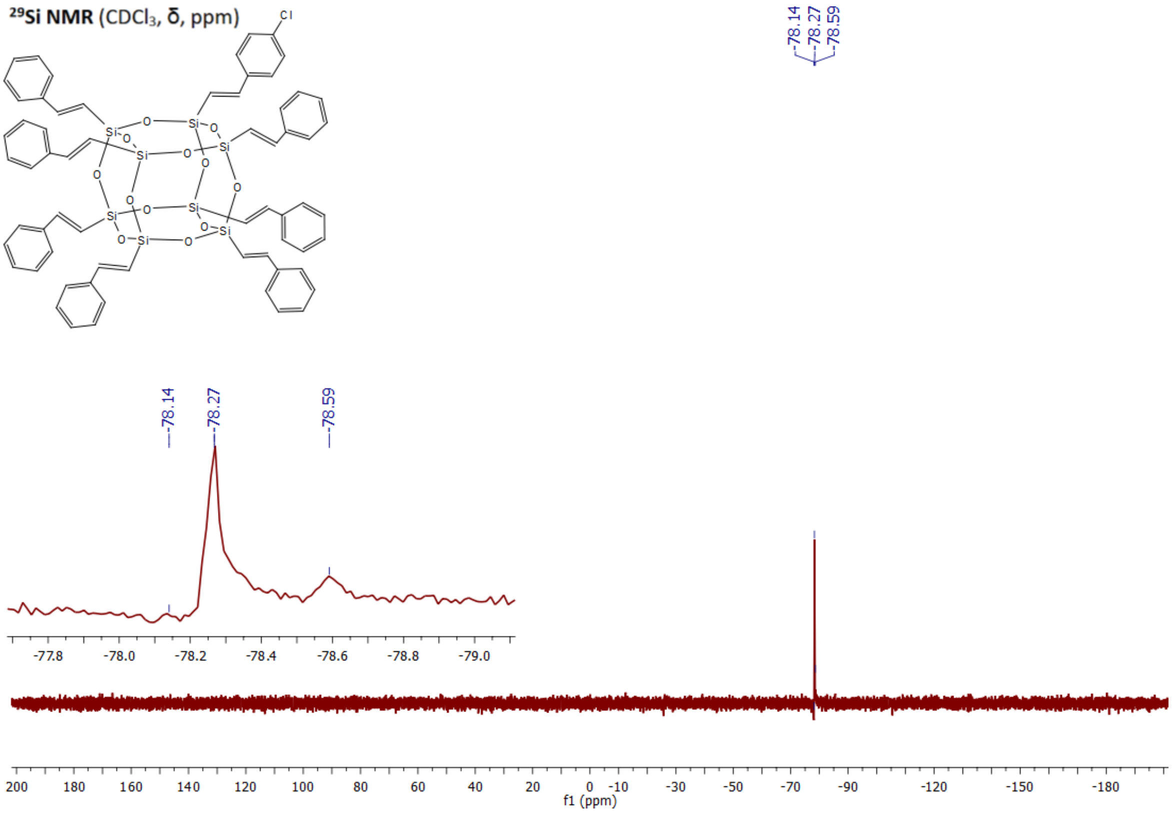

Figure S9. ^{29}Si NMR spectrum of 4a7c.

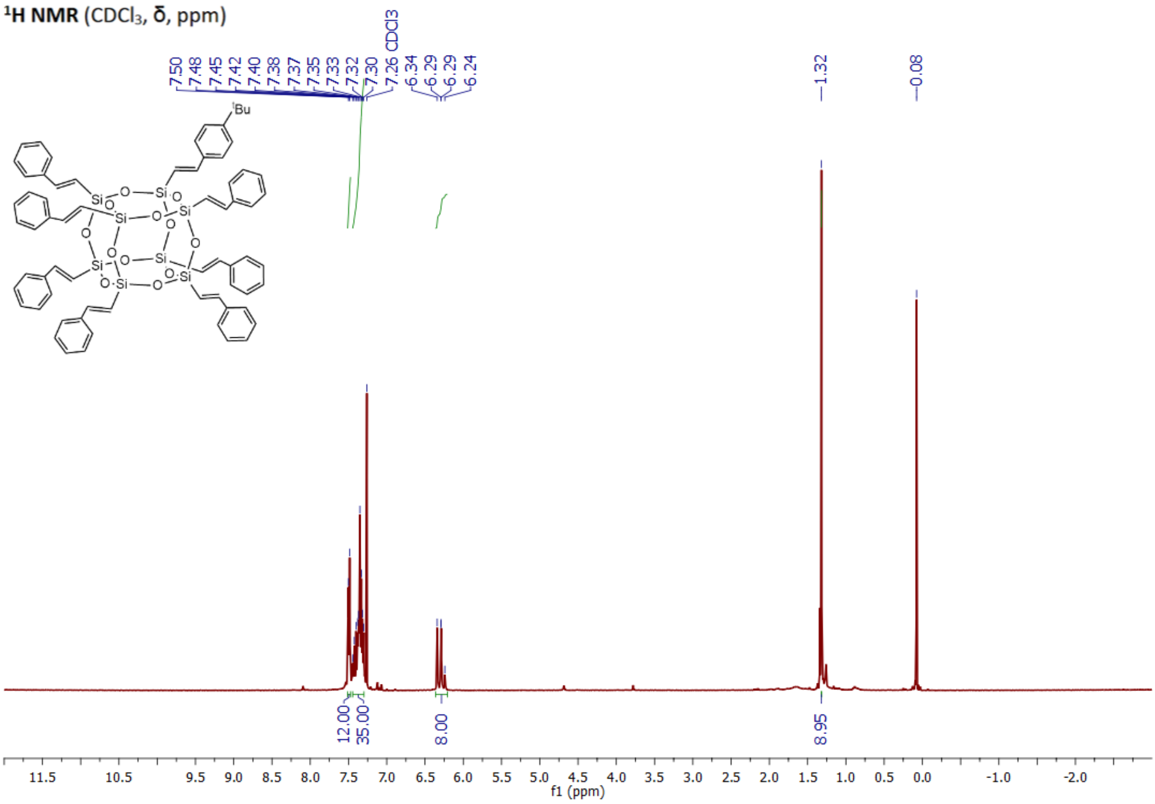

Figure S10. ^{1}H NMR spectrum of 4a$_7$d.

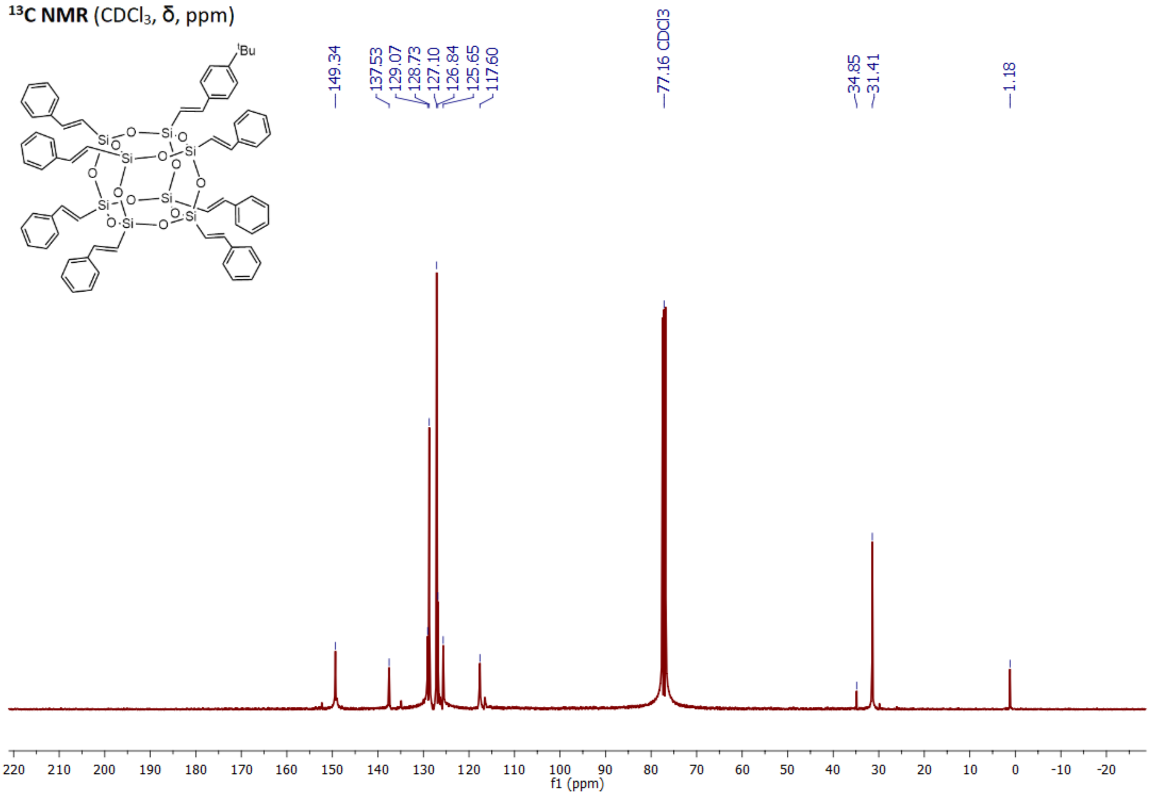

Figure S11. ^{13}C NMR spectrum of 4a$_7$d.

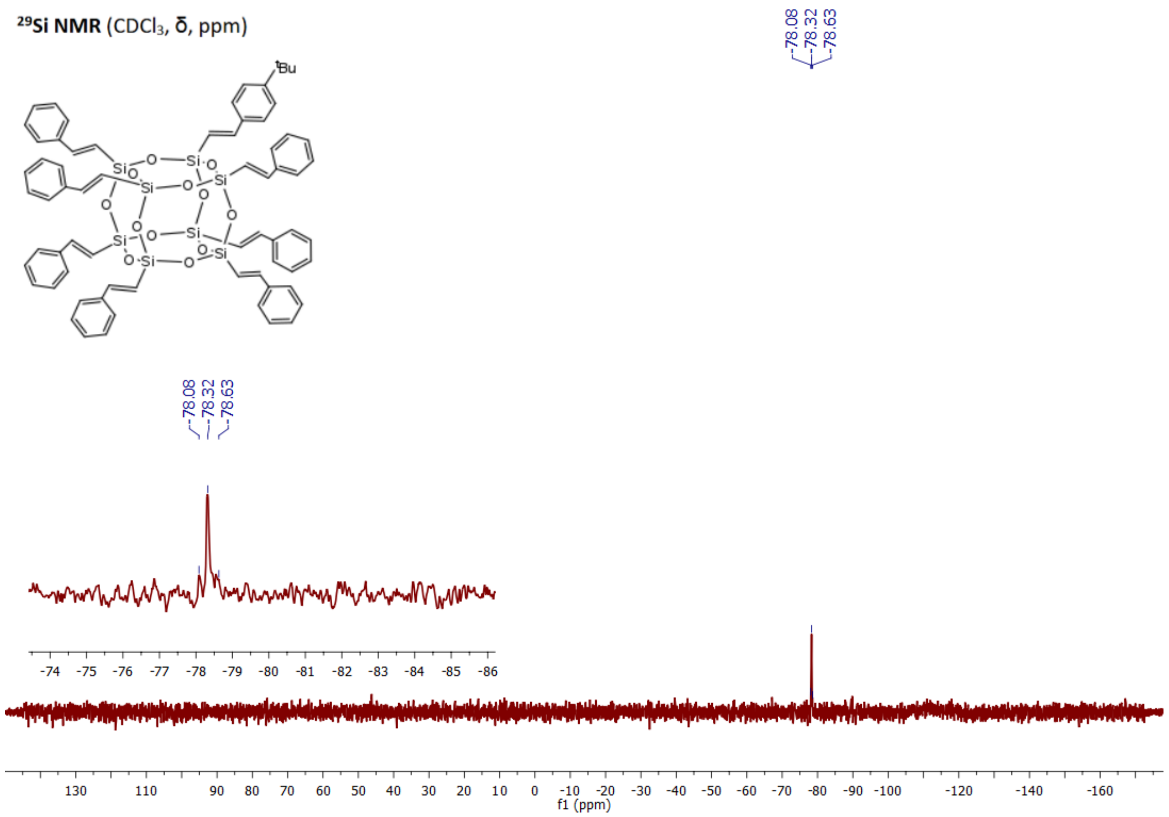

Figure S12. ^{29}Si NMR spectrum of 4a$_7$d.

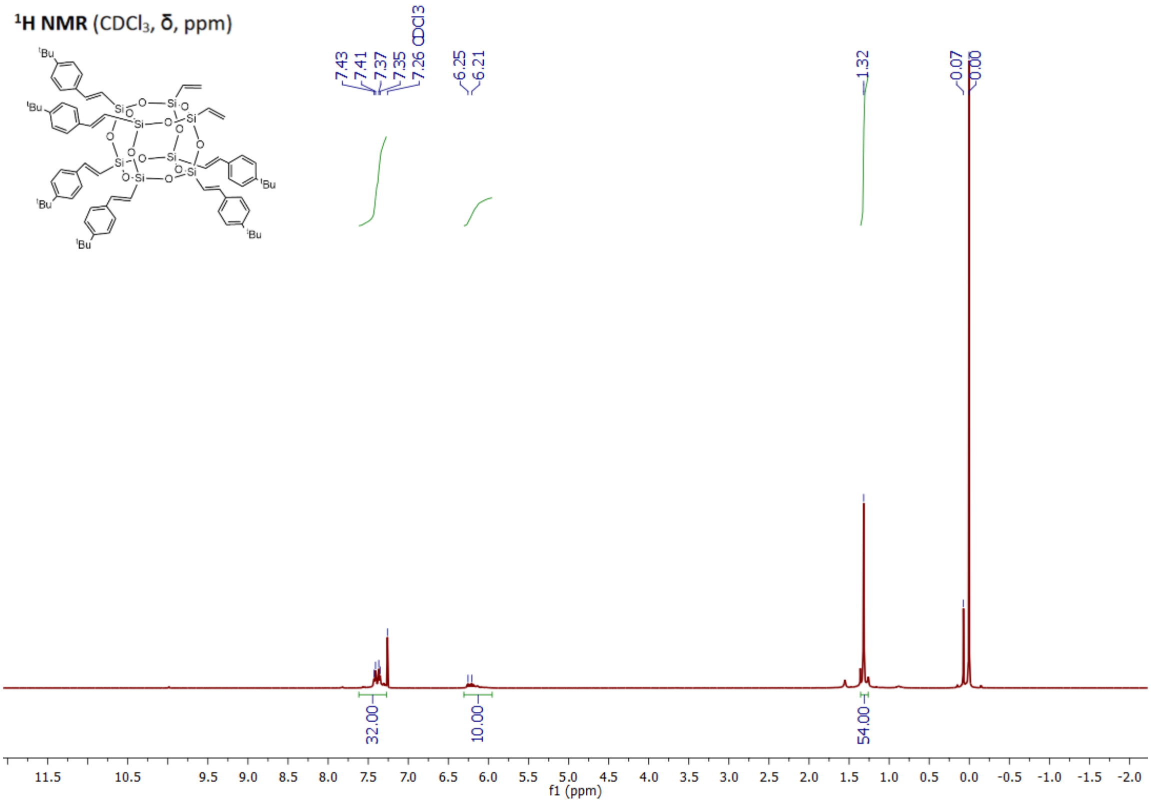

Figure S13. ^{1}H NMR spectrum of 3d$_6$.

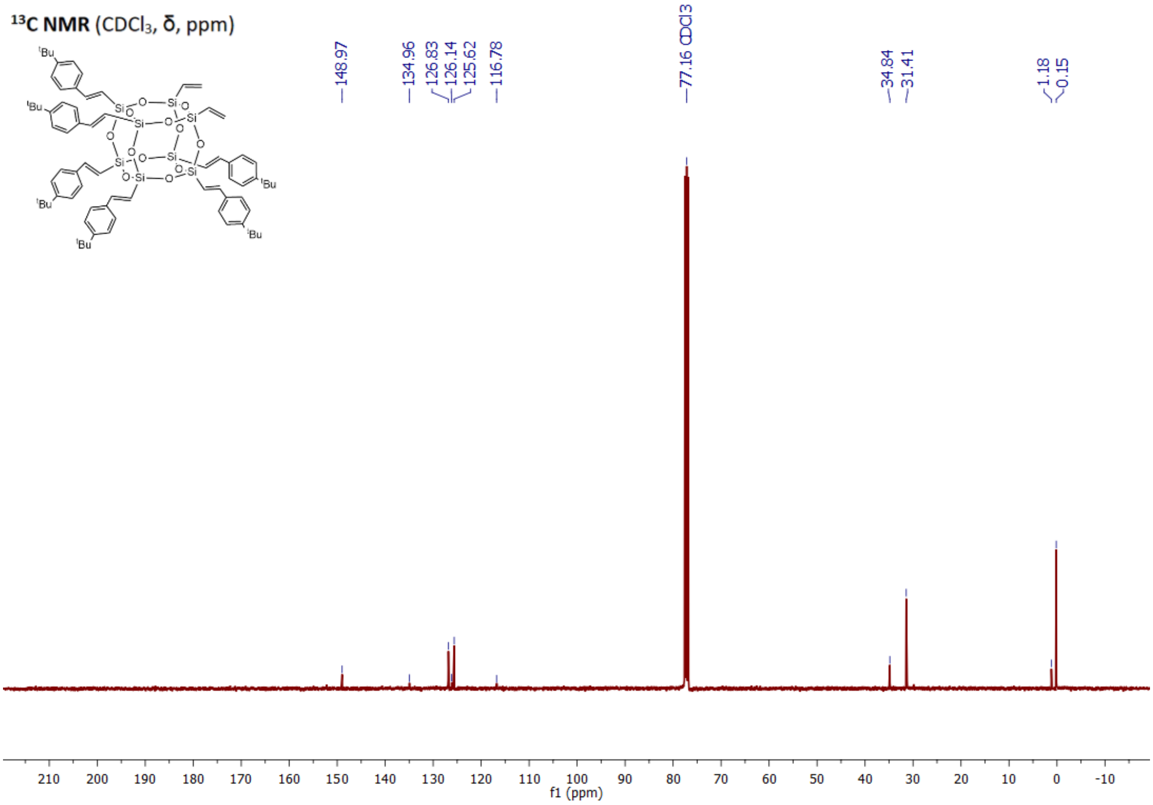

Figure S14. ^{13}C NMR spectrum of 3d6.

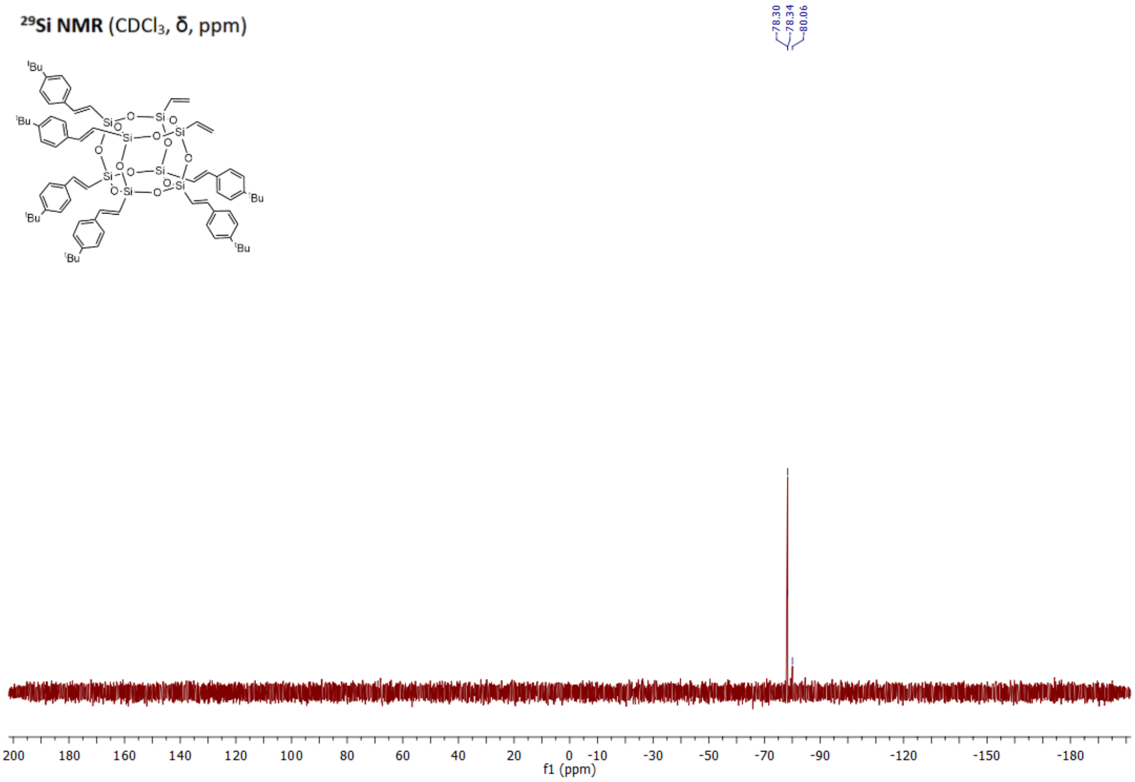

Figure S15. ^{29}Si NMR spectrum of 3d6.

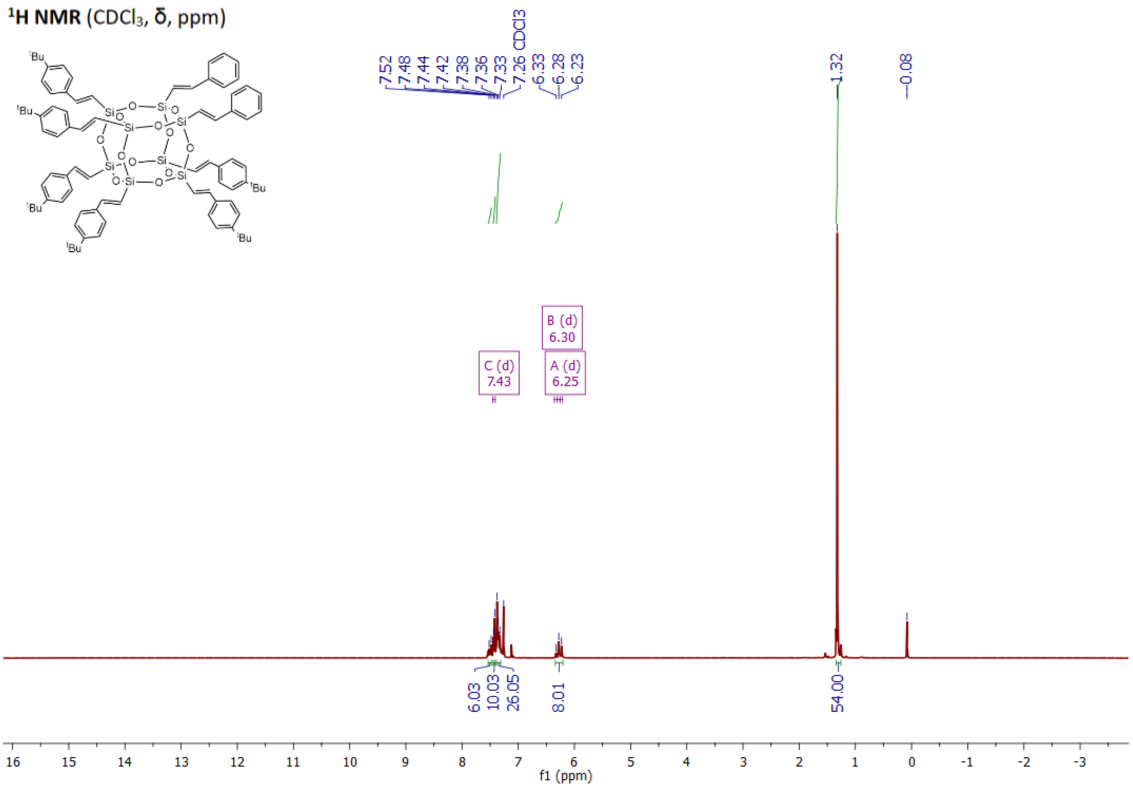

Figure S16. ^{1}H NMR spectrum of 4d6a2.

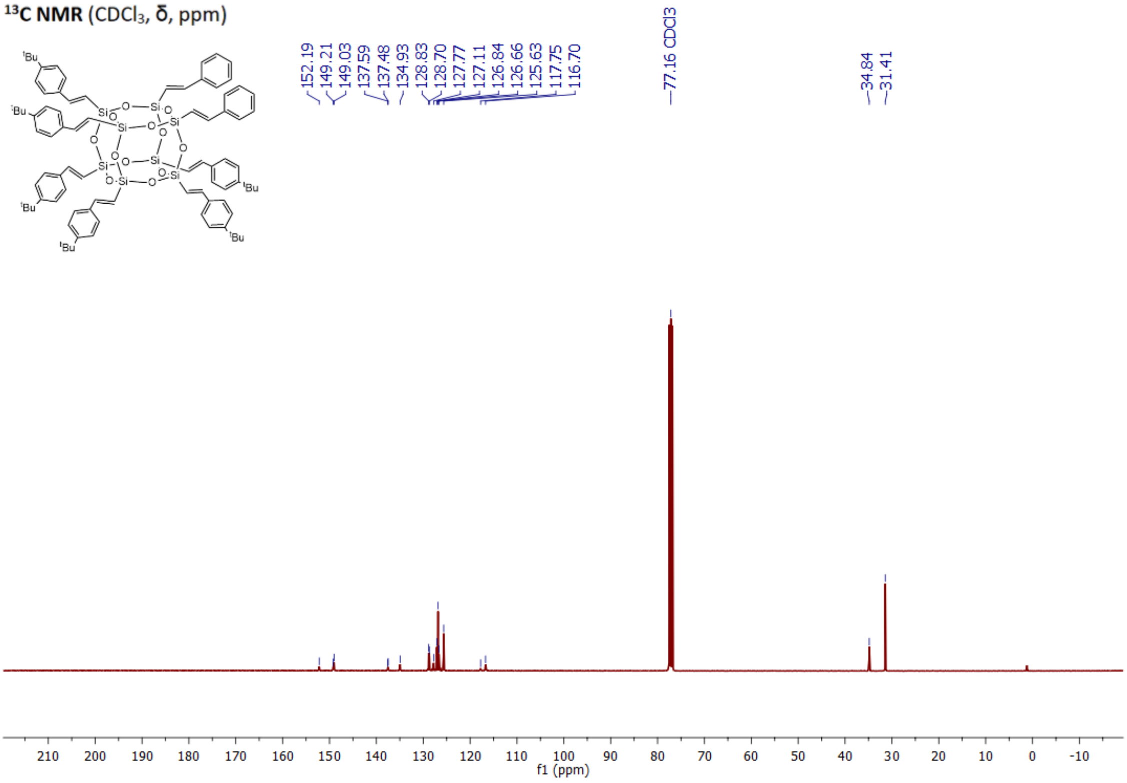

Figure S17. ^{13}C NMR spectrum of 4d6a2.

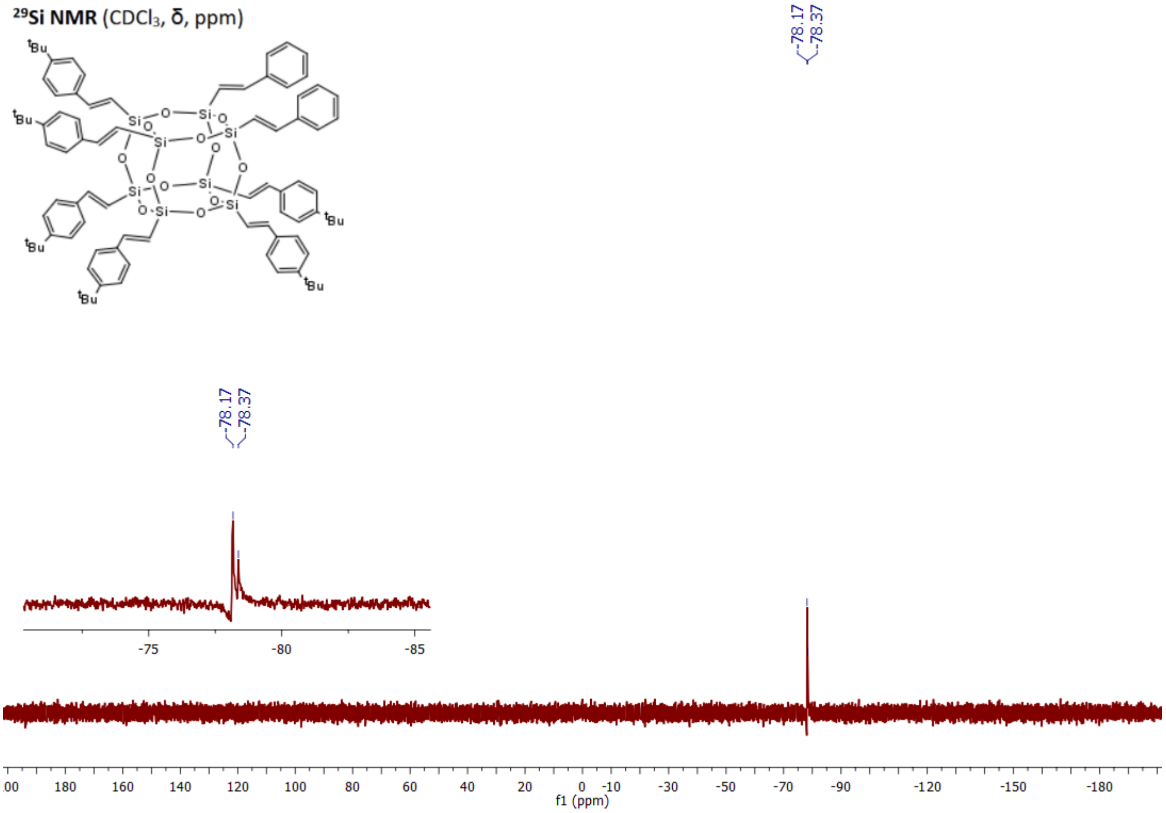

Figure S18. ^{29}Si NMR spectrum of 4d$_6$a$_2$.

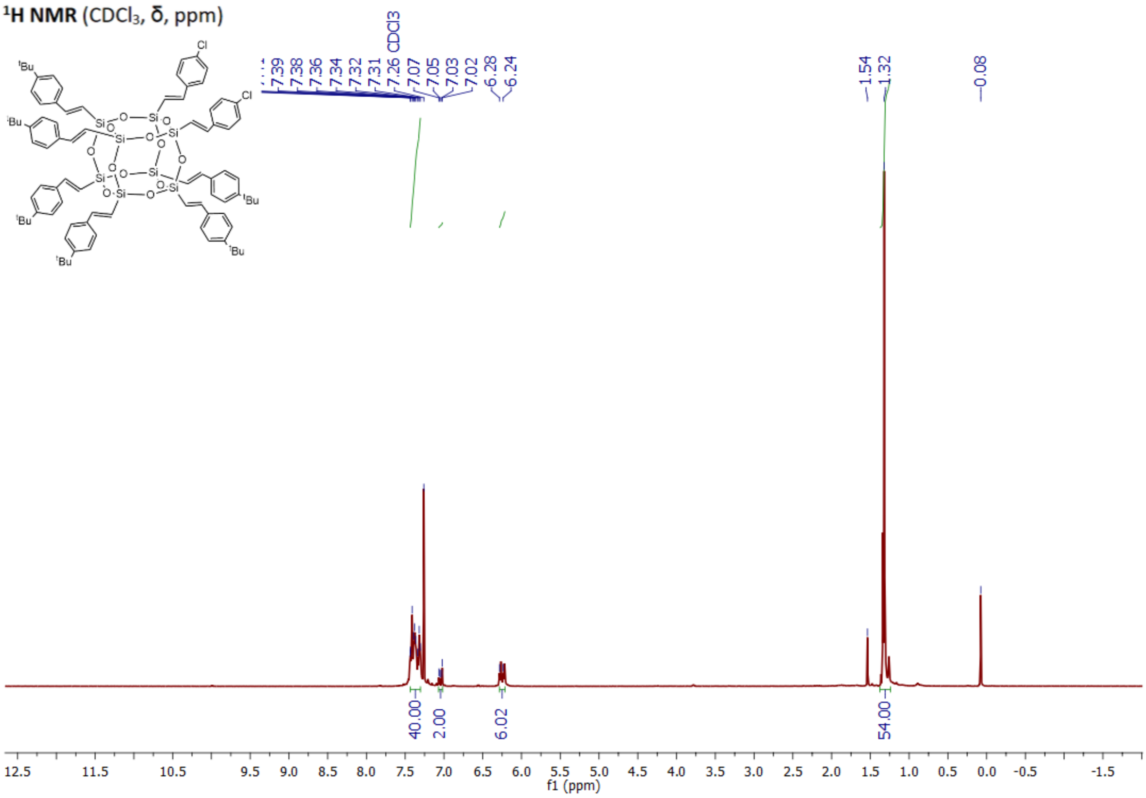

Figure S19. ^{1}H NMR spectrum of 4d$_6$c$_2$.

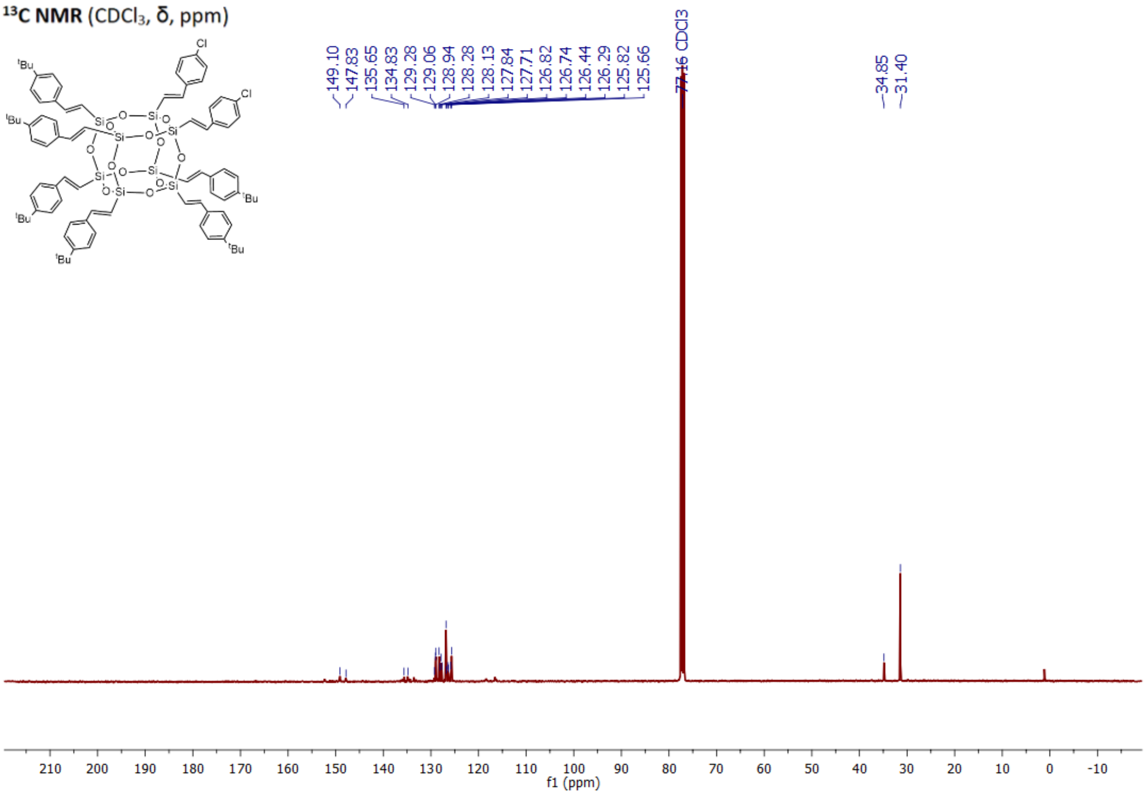

Figure S20. ^{13}C NMR spectrum of 4d6c2.

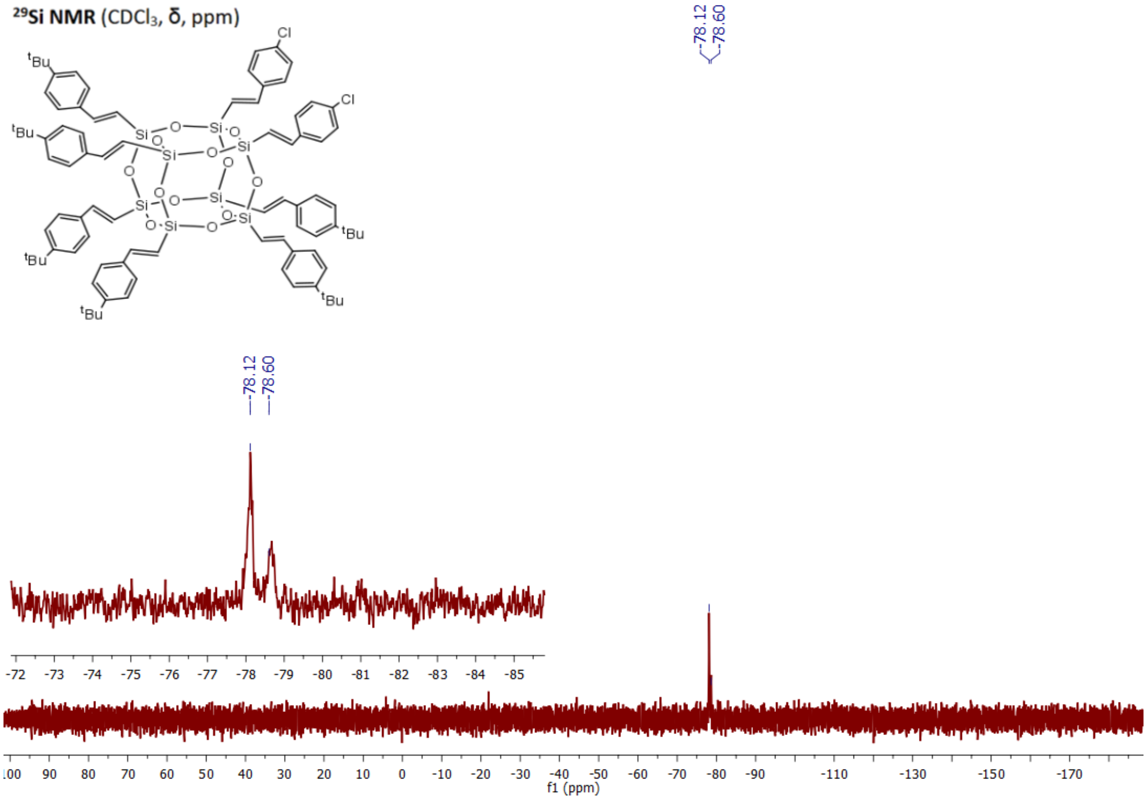

Figure S21. ^{29}Si NMR spectrum of 4d6c2.

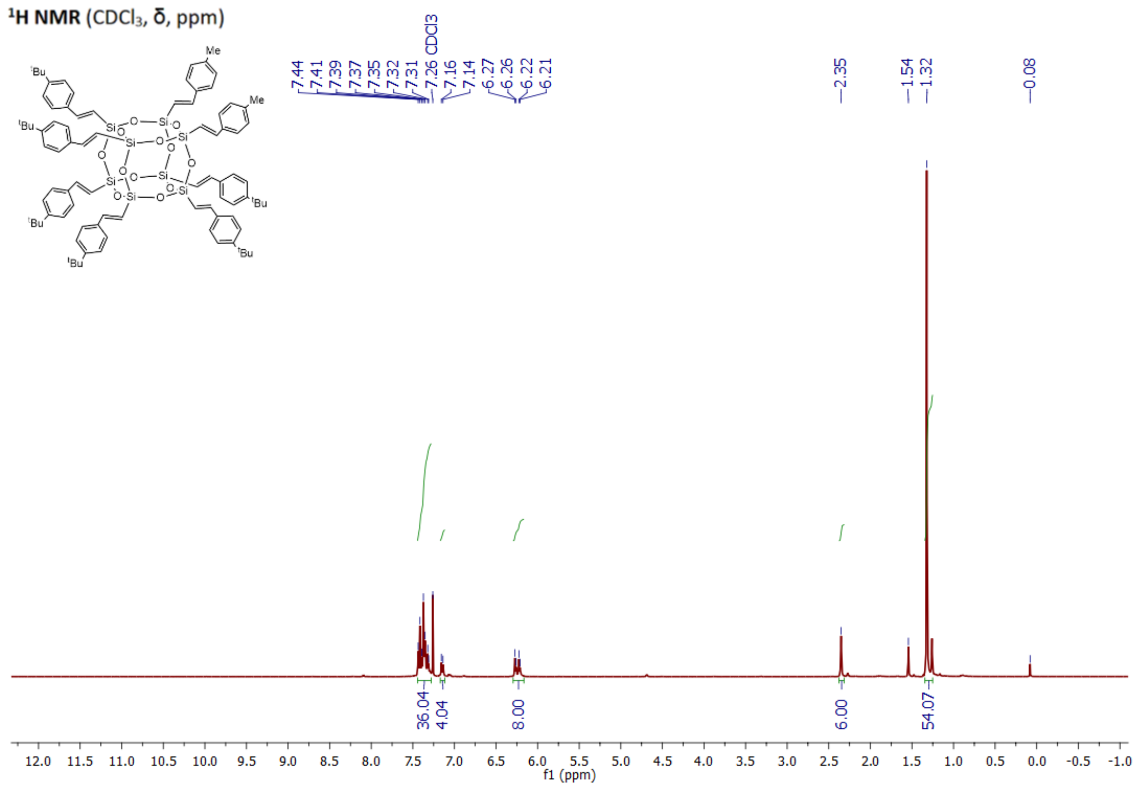

Figure S22. ^{1}H NMR spectrum of $4d_6b_2$.

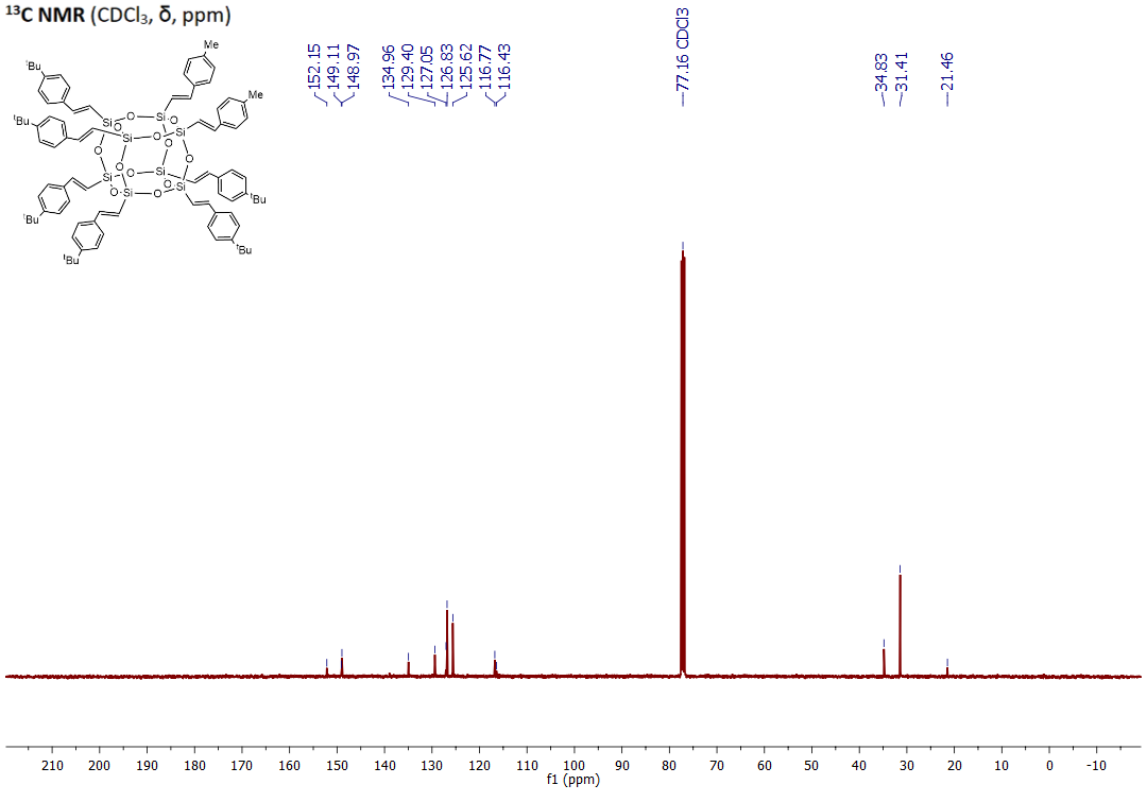

Figure S23. ^{13}C NMR spectrum of $4d_6b_2$.

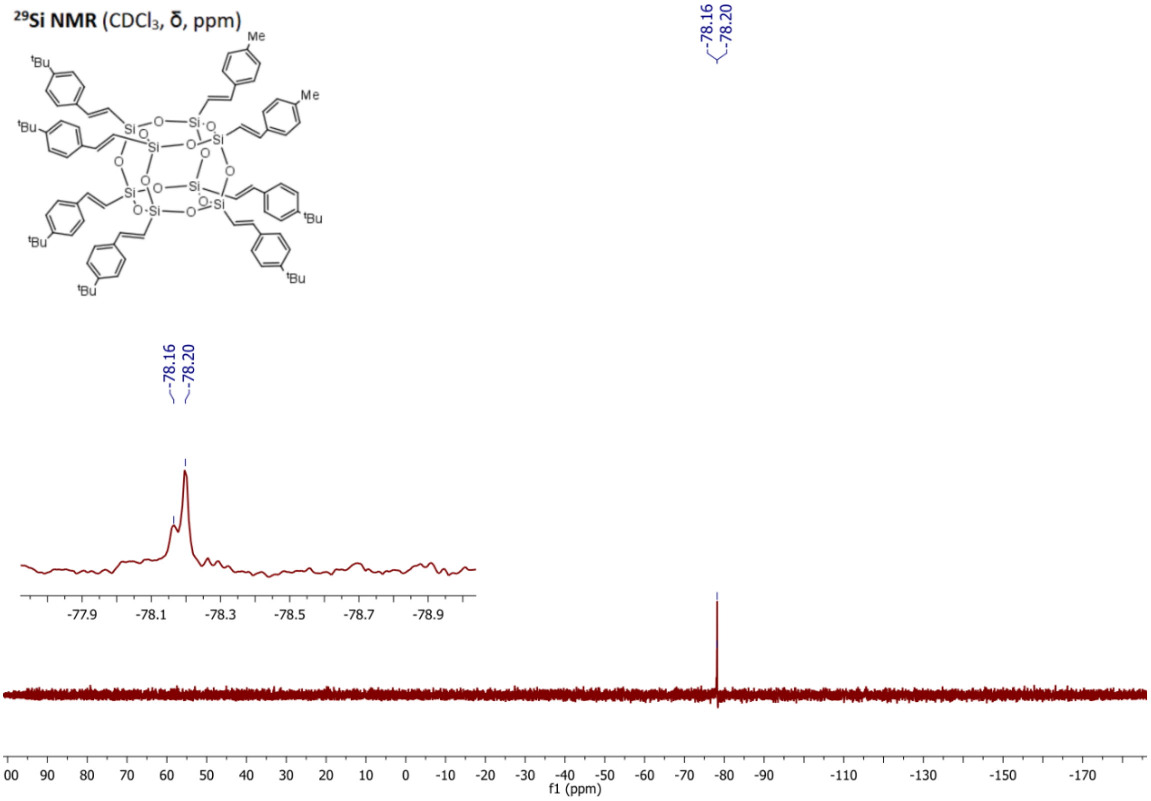

Figure S24. ^{29}Si NMR spectrum of $4d_6b_2$.

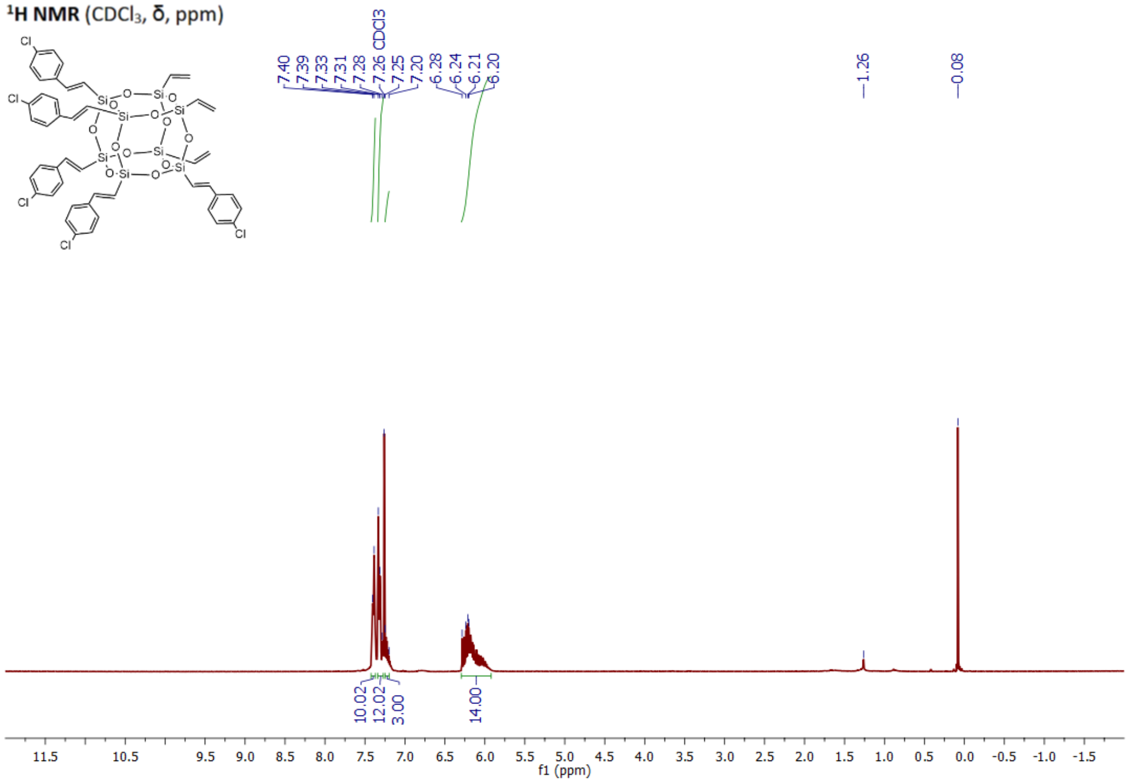

Figure S25. ^{1}H NMR spectrum of $3c_5$.

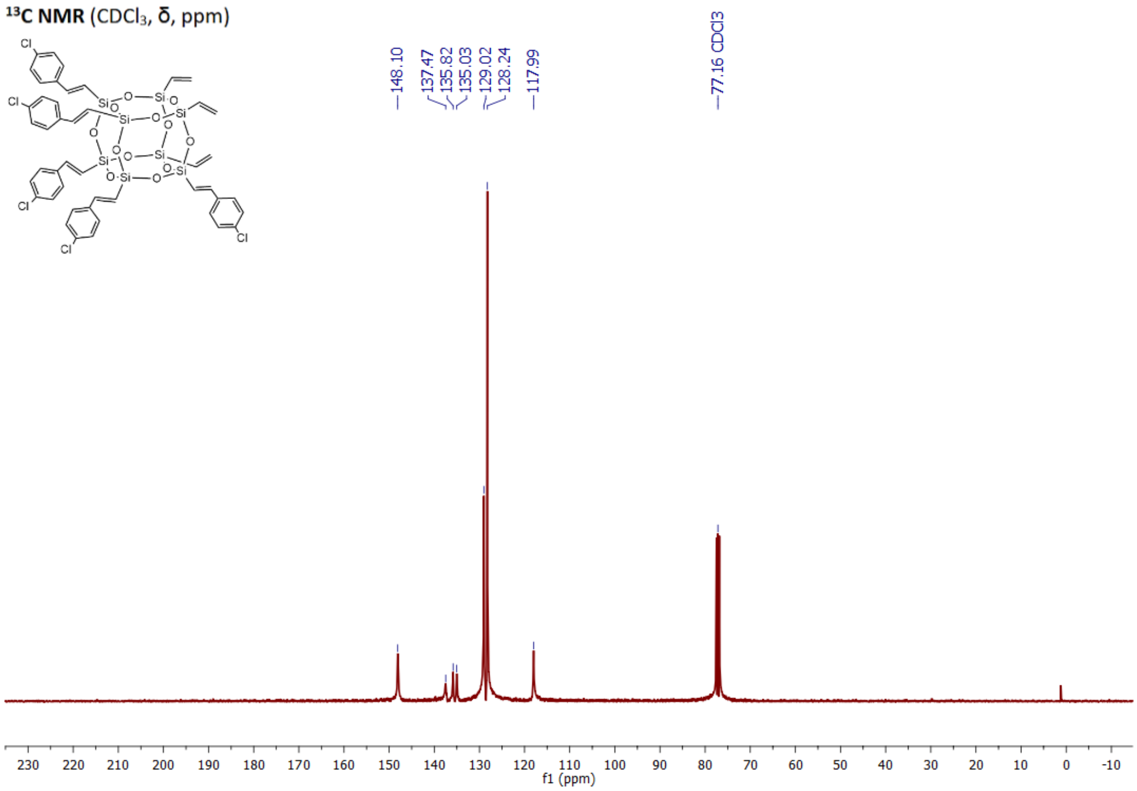

Figure S26. ^{13}C NMR spectrum of 3c5.

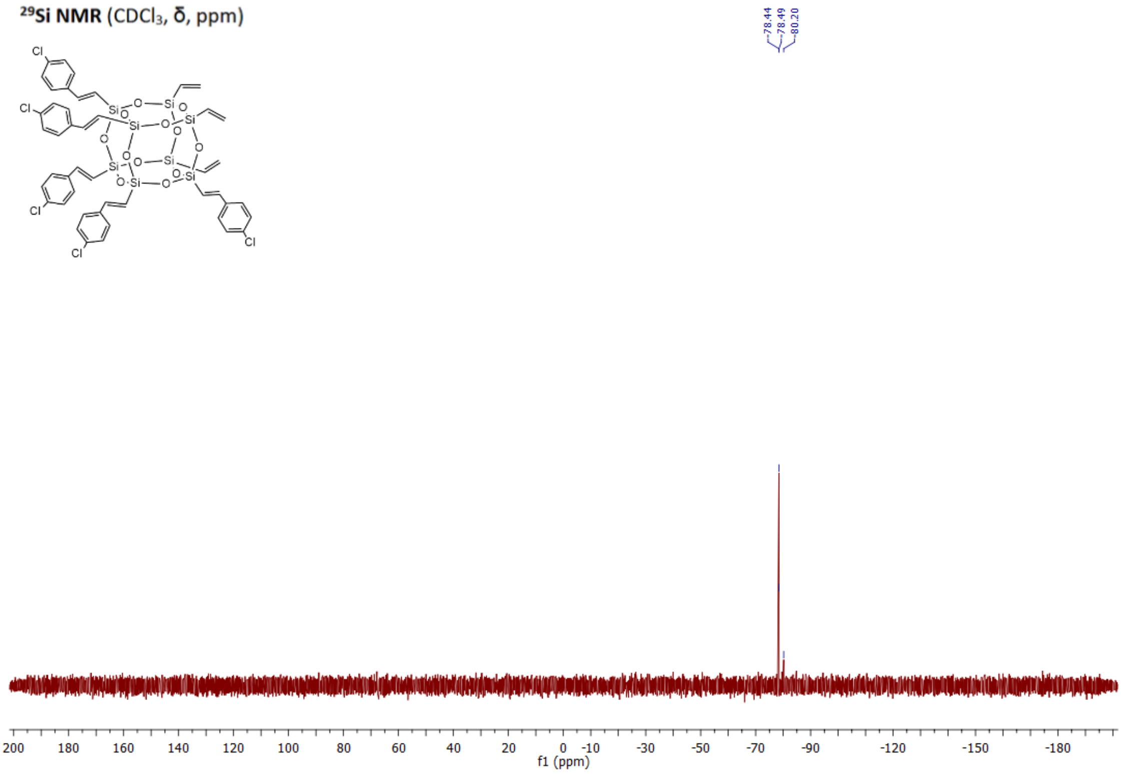

Figure S27. ^{29}Si NMR spectrum of 3c5.

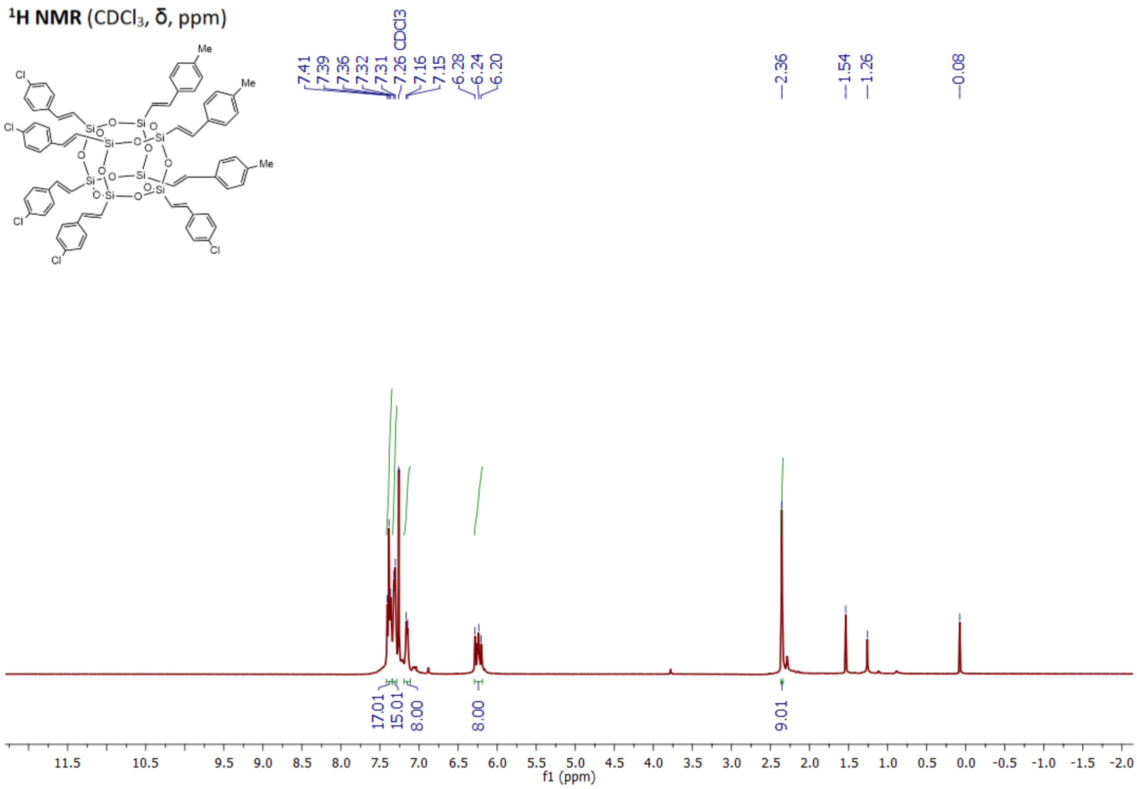

Figure S28. ^{1}H NMR spectrum of 4c5b3.

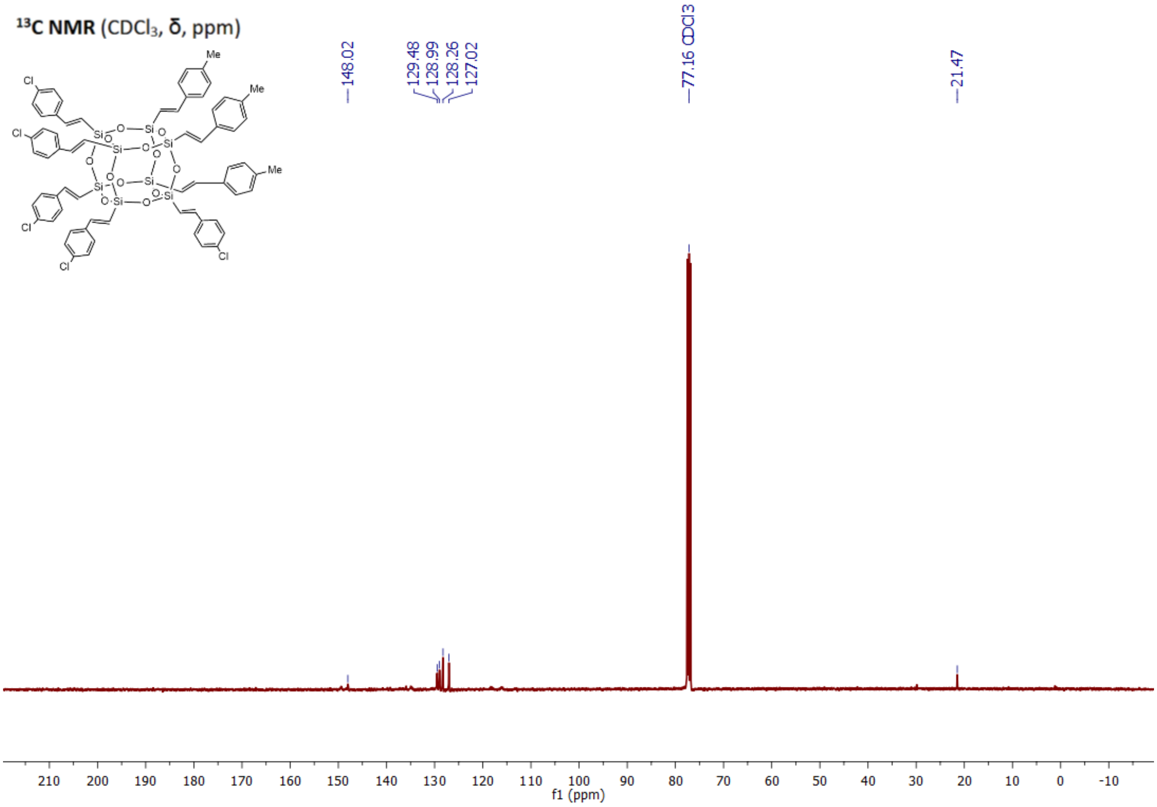

Figure S29. ^{13}C NMR spectrum of 4c5b3.

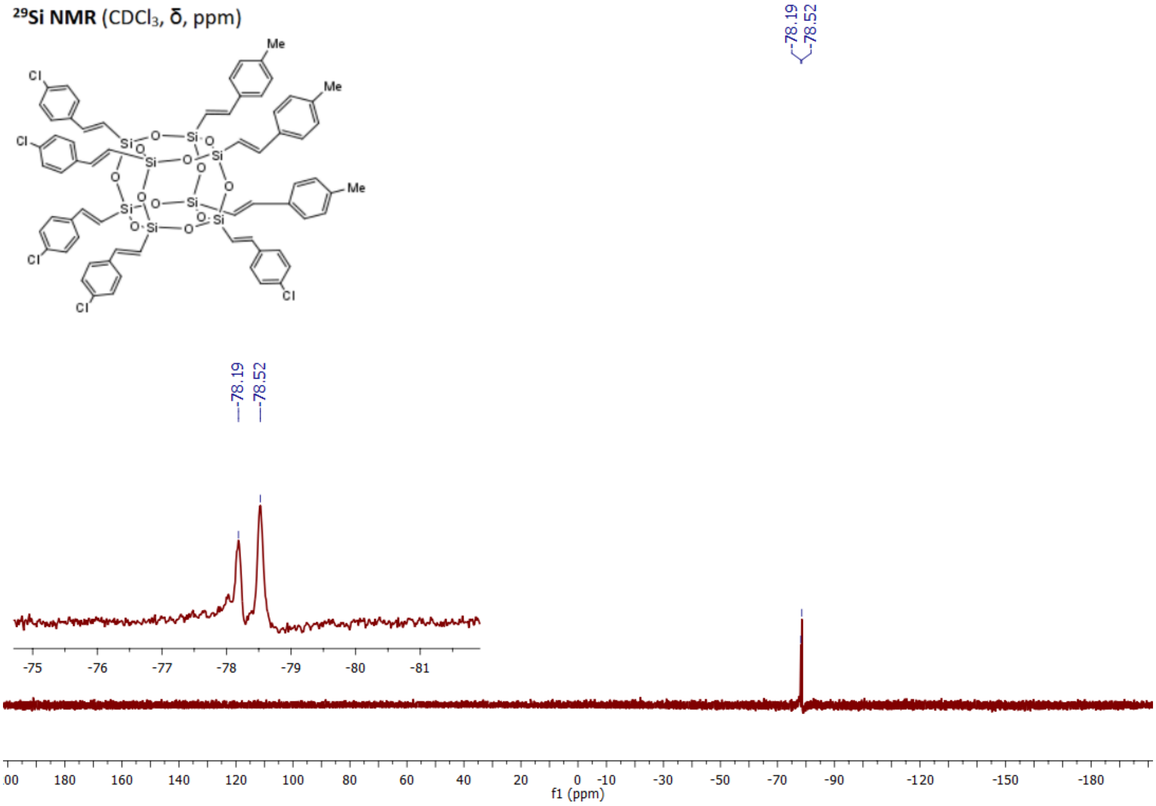

Figure S30. ^{29}Si NMR spectrum of 4c$_5$b$_3$.

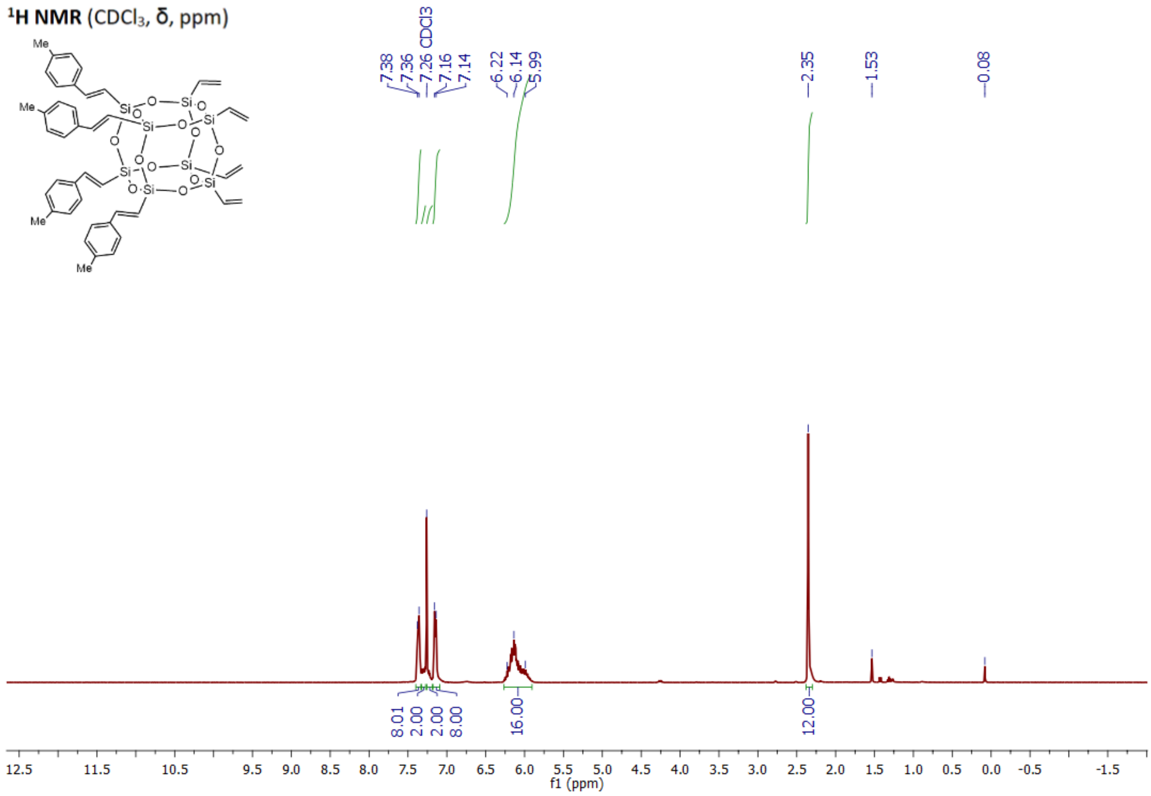

Figure S31. ^{1}H NMR spectrum of 3b$_4$.

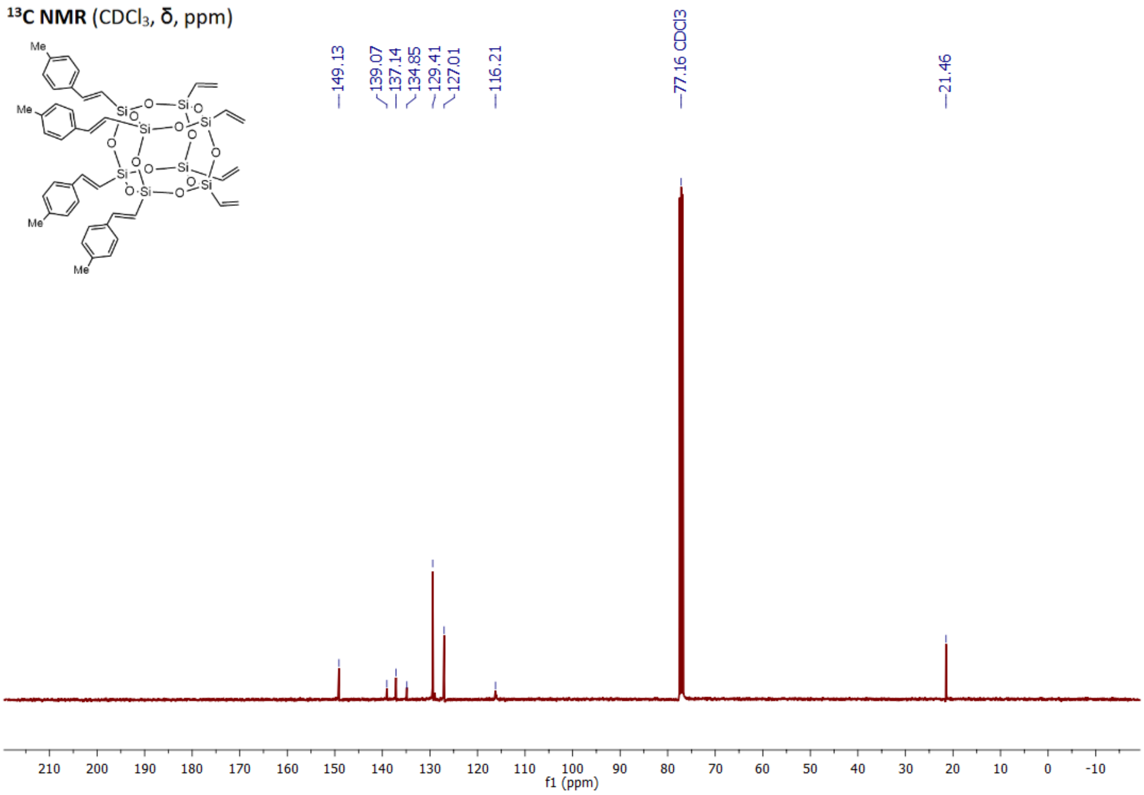

Figure 32. ^{13}C NMR spectrum of 3b4.

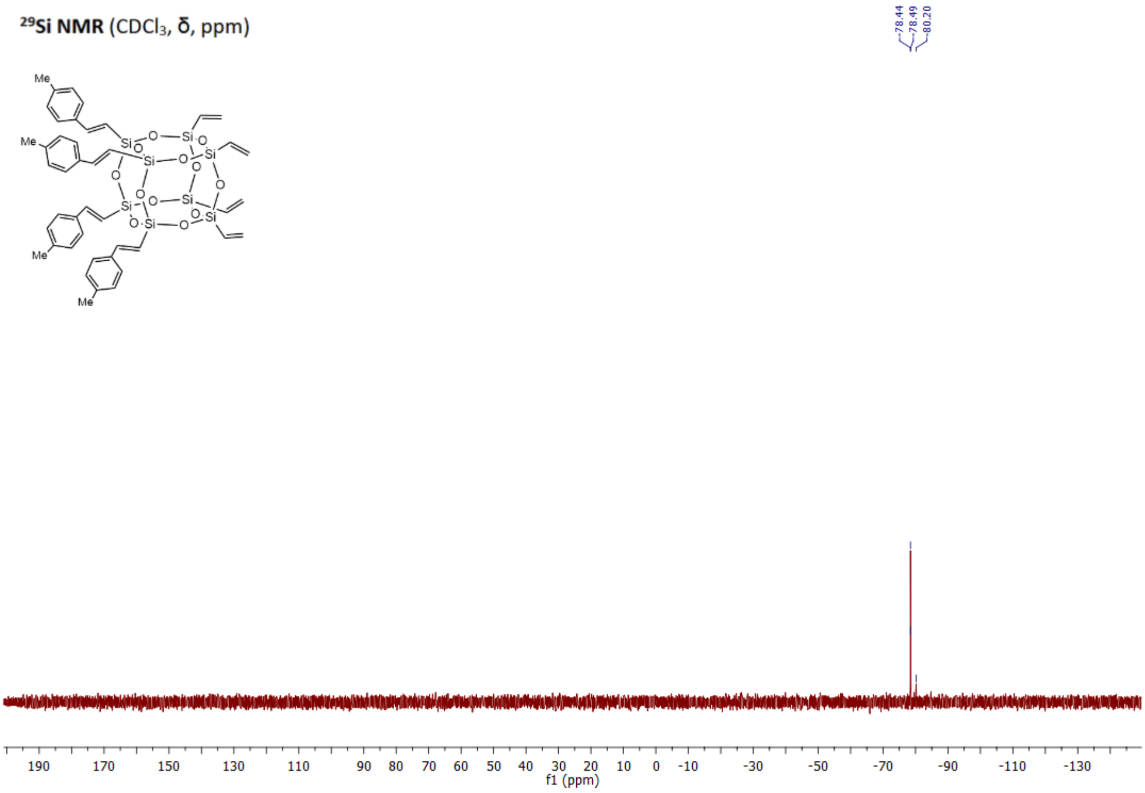

Figure S33. ^{29}Si NMR spectrum of 3b4.

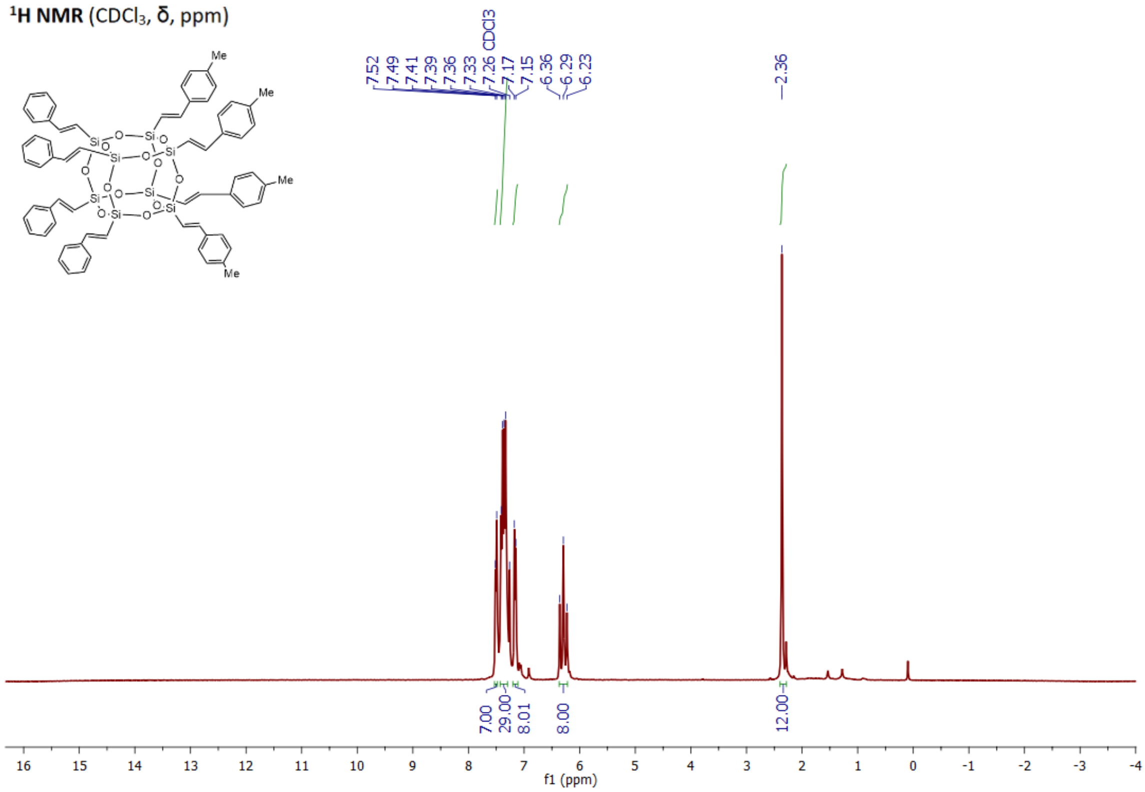

Figure S34. ¹H NMR spectrum of 4b4a4.

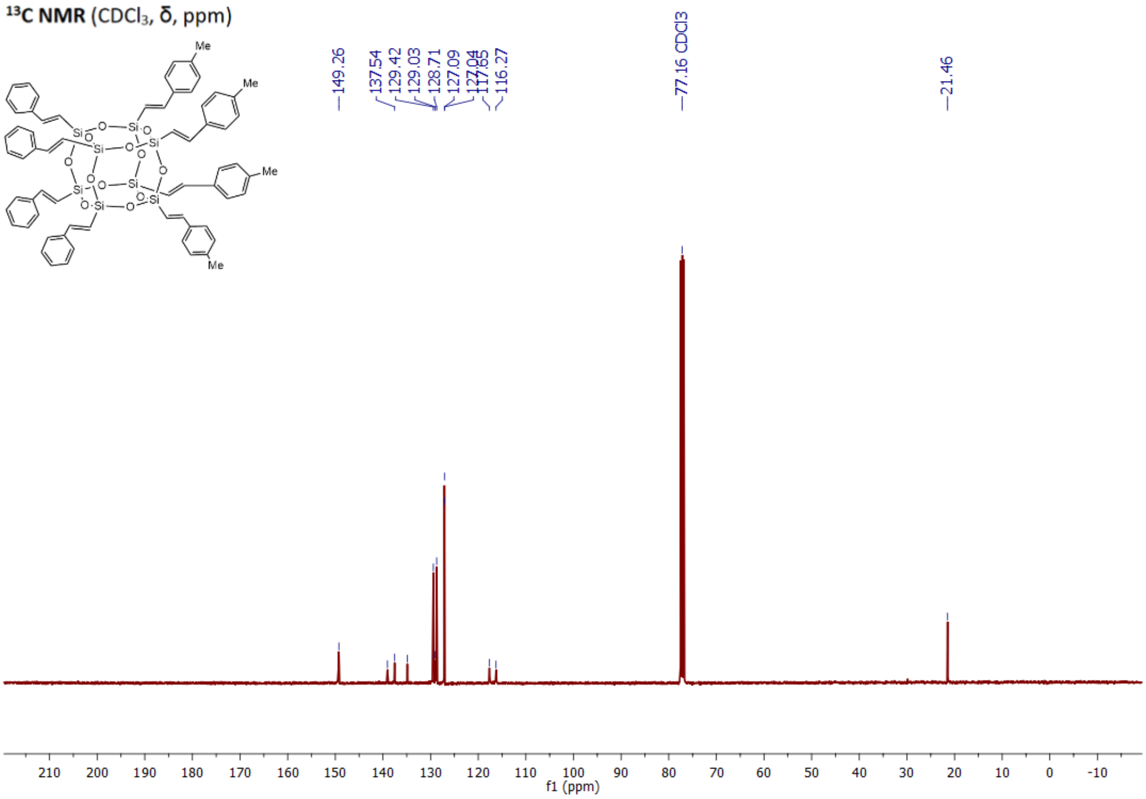

Figure S35. ¹³C NMR spectrum of 4b4a4.

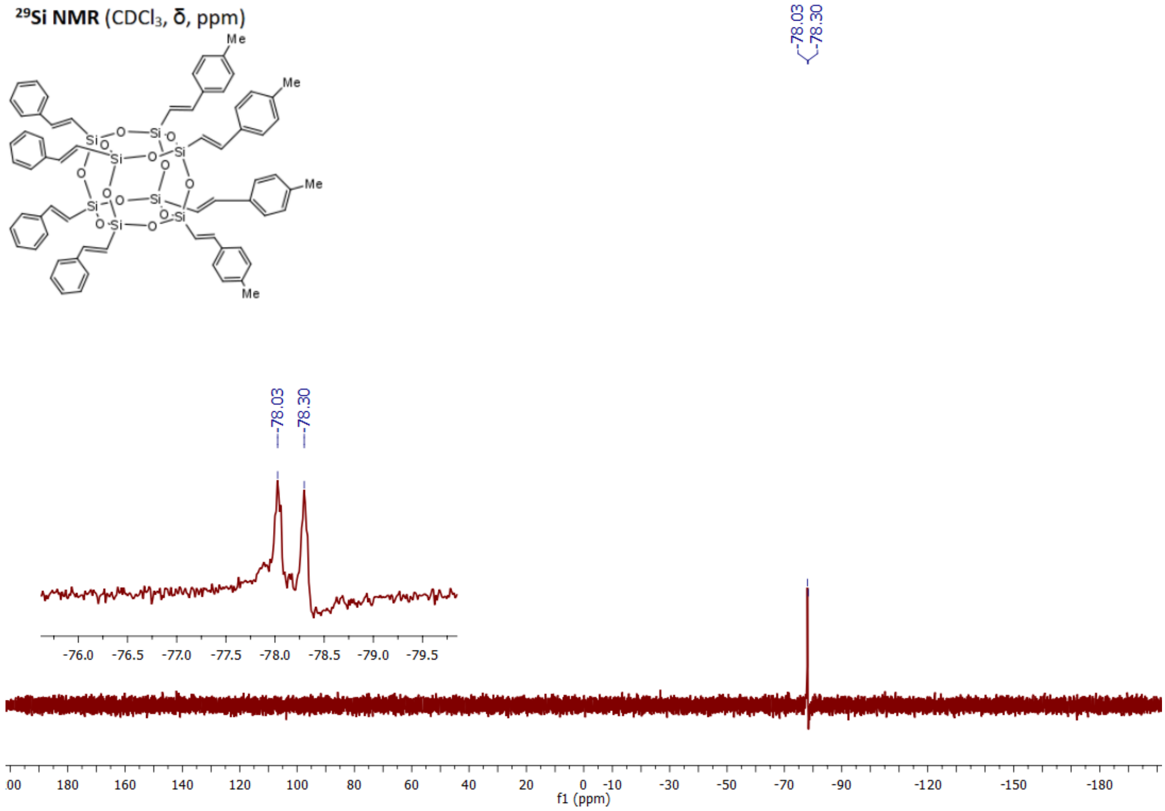

Figure S36. ^{29}Si NMR spectrum of 4b4a4.

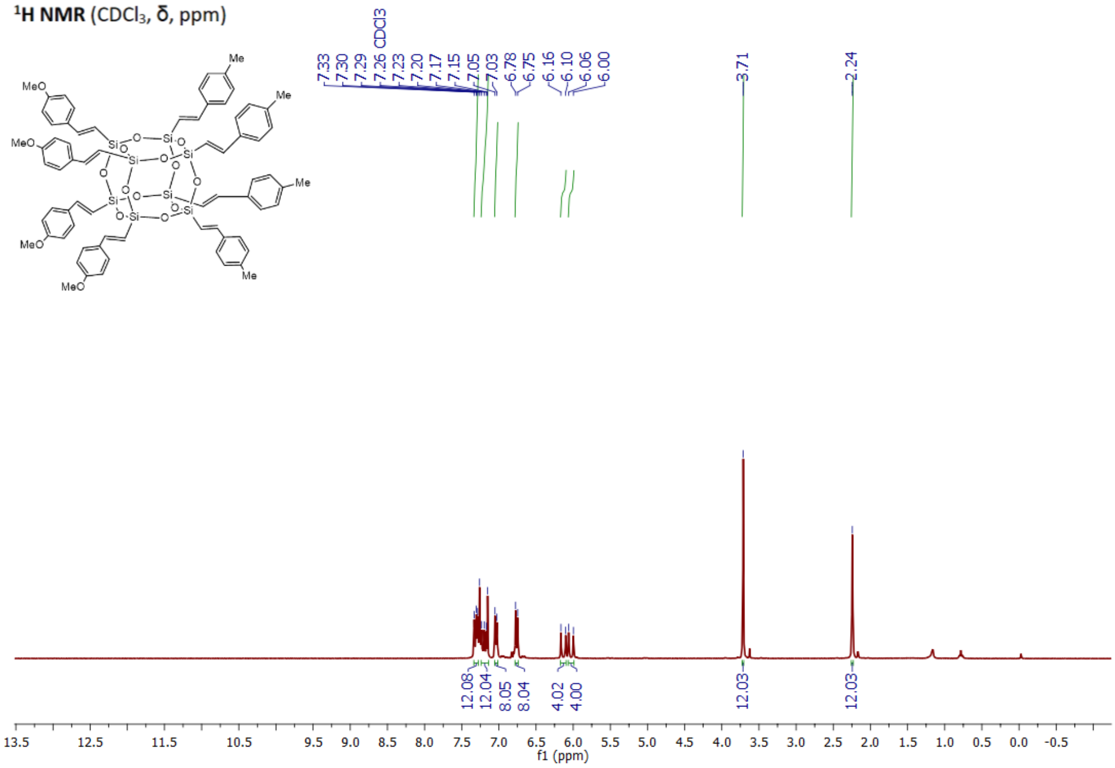

Figure S37. ^{1}H NMR spectrum of 4b4e4.

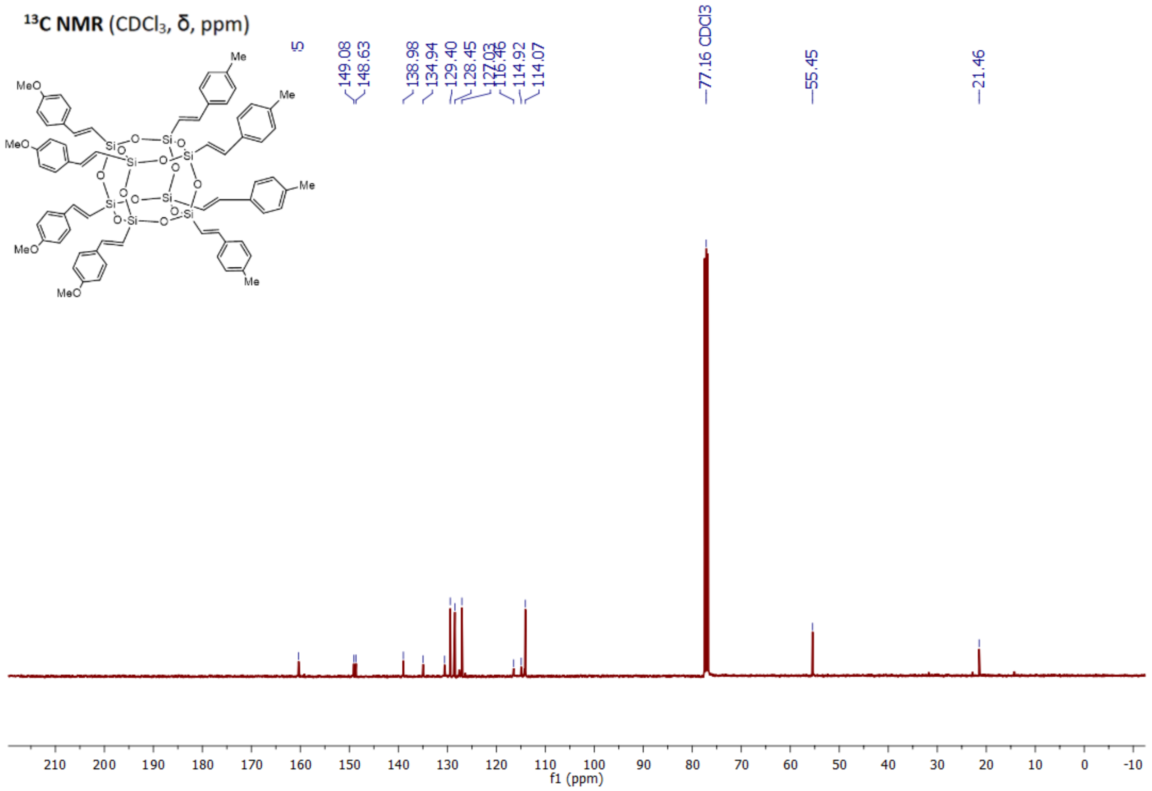

Figure S38. ^{13}C NMR spectrum of $4b_4e_4$.

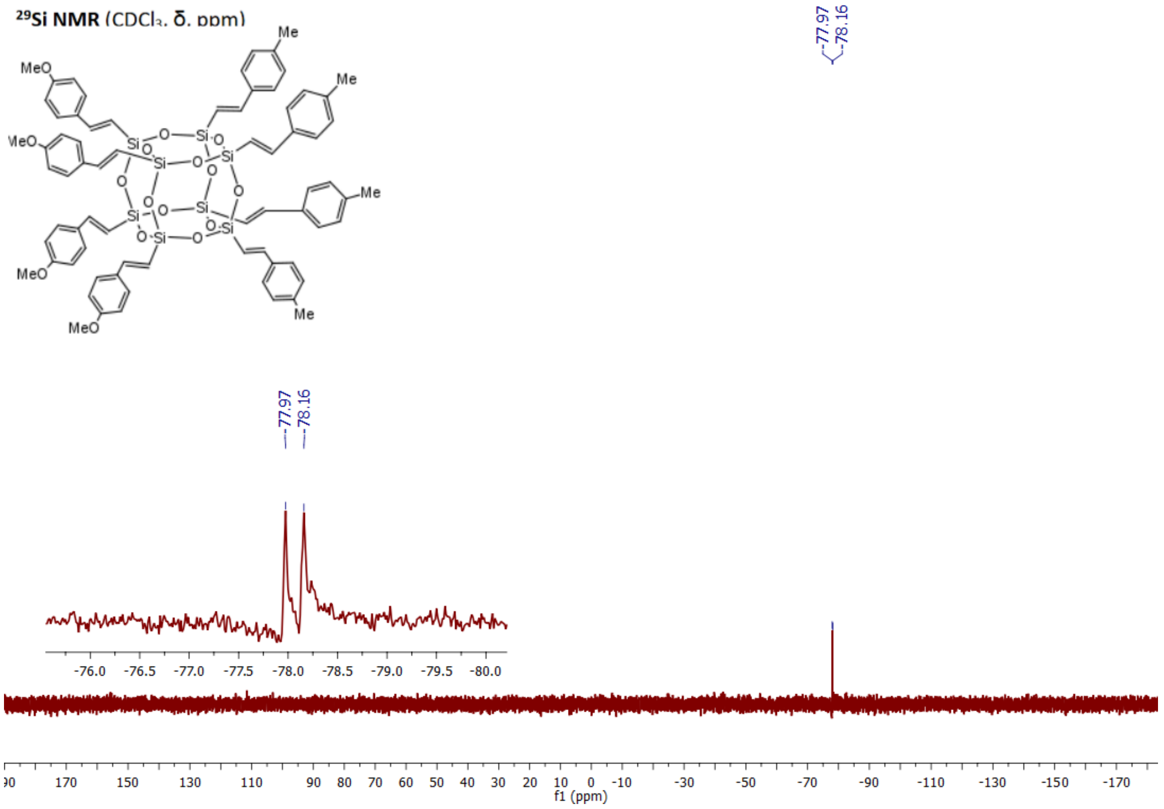

Figure S39. ^{29}Si NMR spectrum of $4b_4e_4$.

3.2. NMR Spectra of Silylative Coupling Products

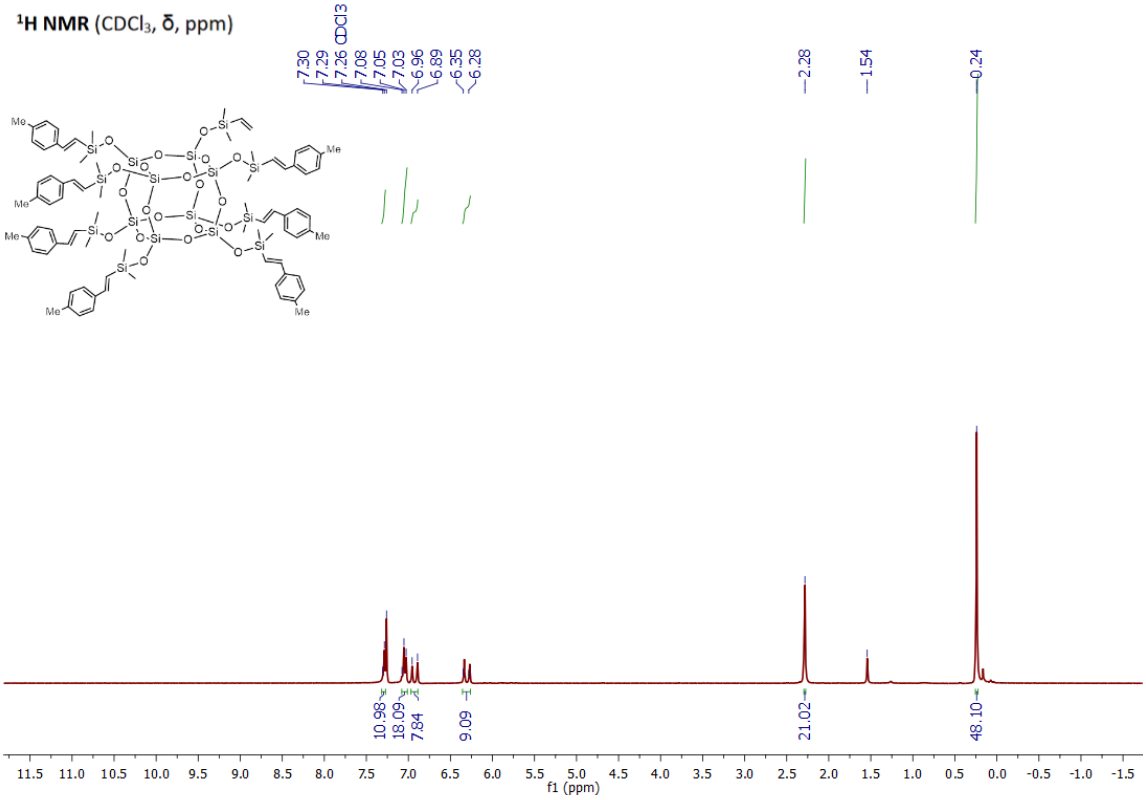

Figure S40. ¹H NMR spectrum of 6b7.

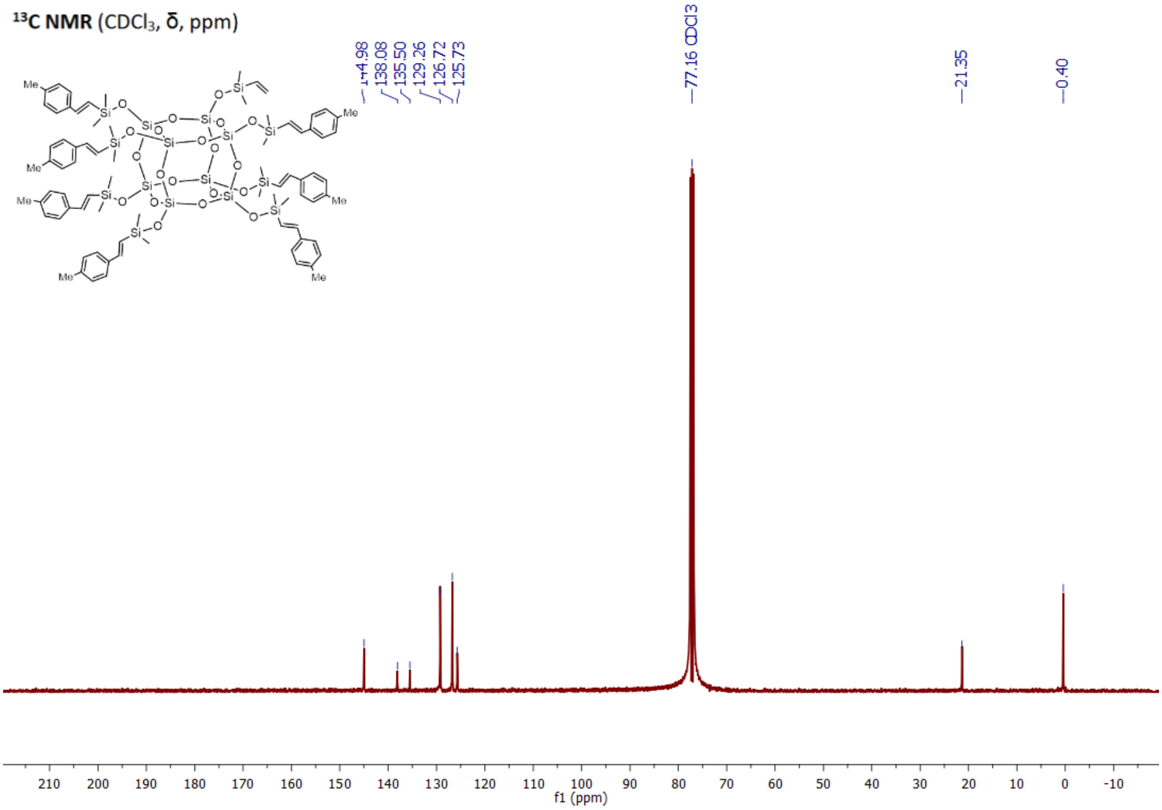

Figure S41. ¹³C NMR spectrum of 6b7.

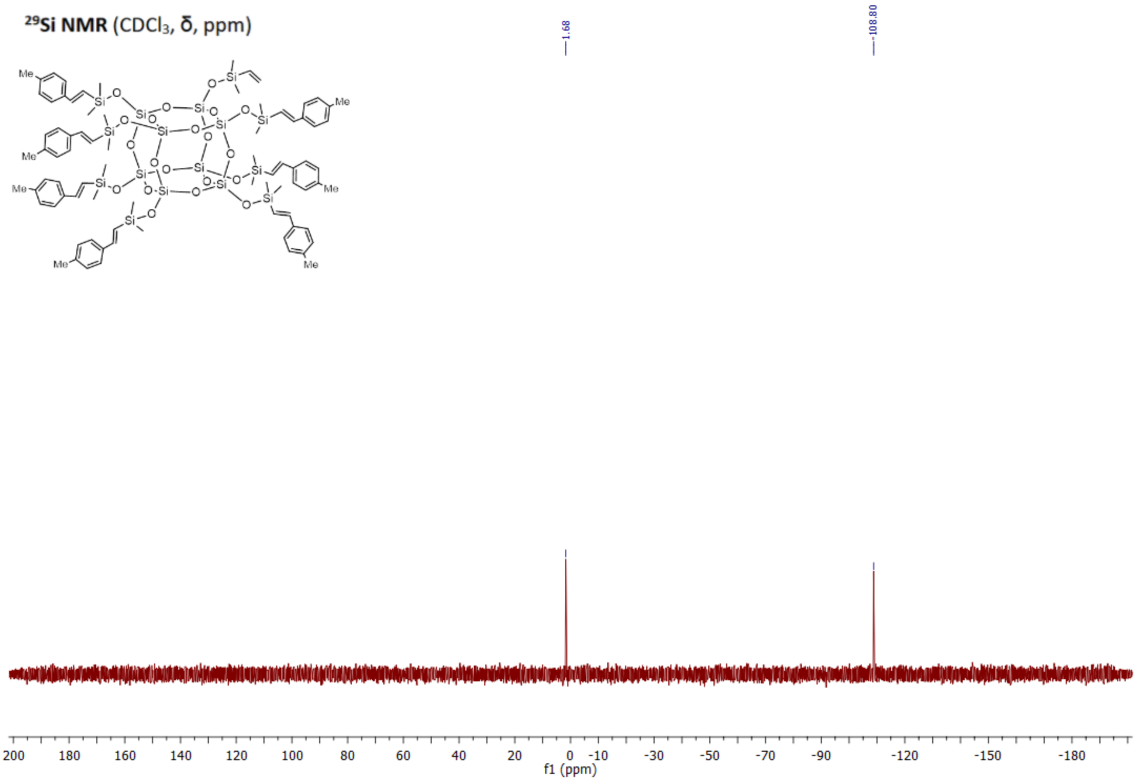

Figure S42. ^{29}Si NMR spectrum of 6b$_7$.

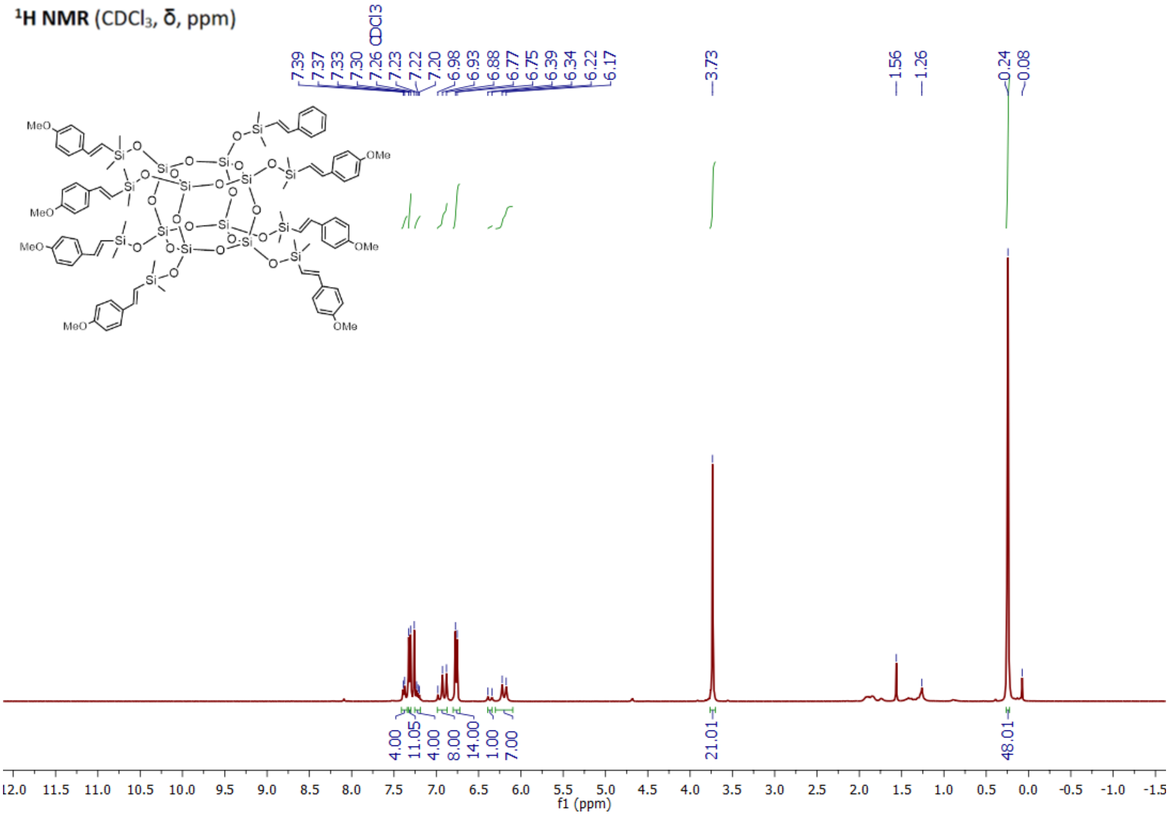

Figure S43. ^{1}H NMR spectrum of 7e$_7$a$_1$.

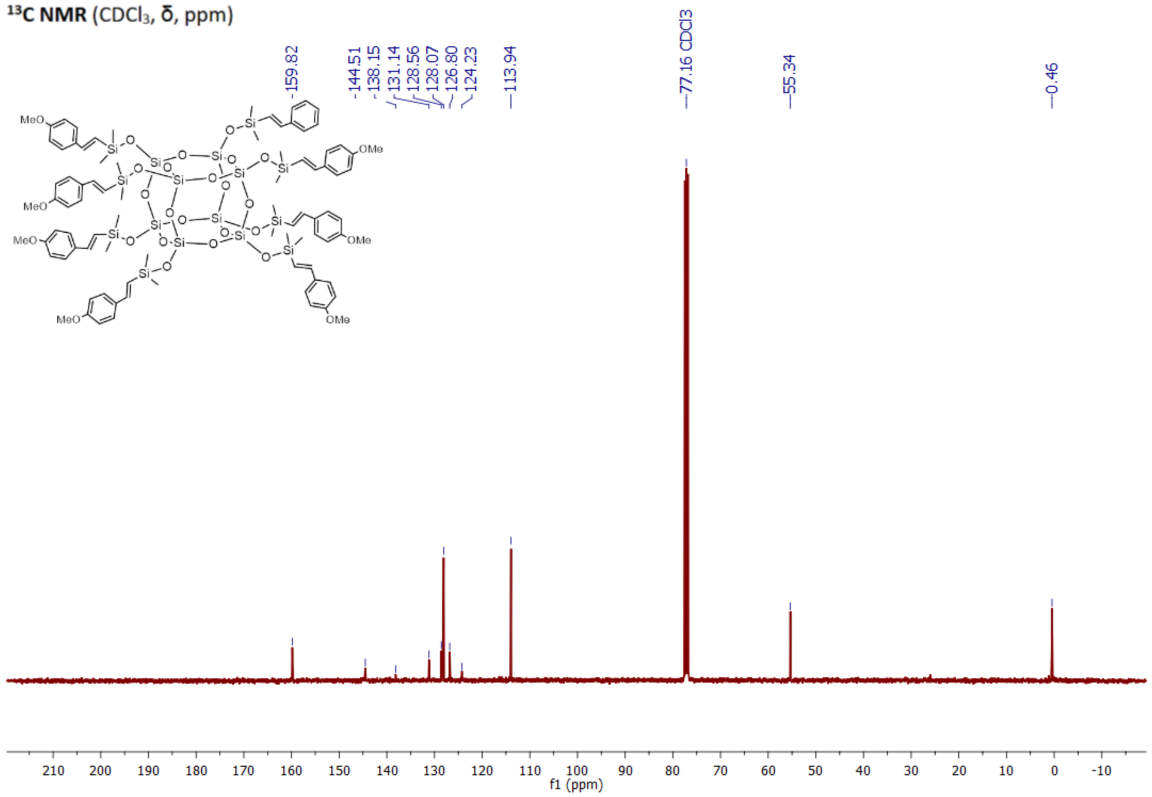

Figure S44. ^{13}C NMR spectrum of 7e$_7$a$_1$.

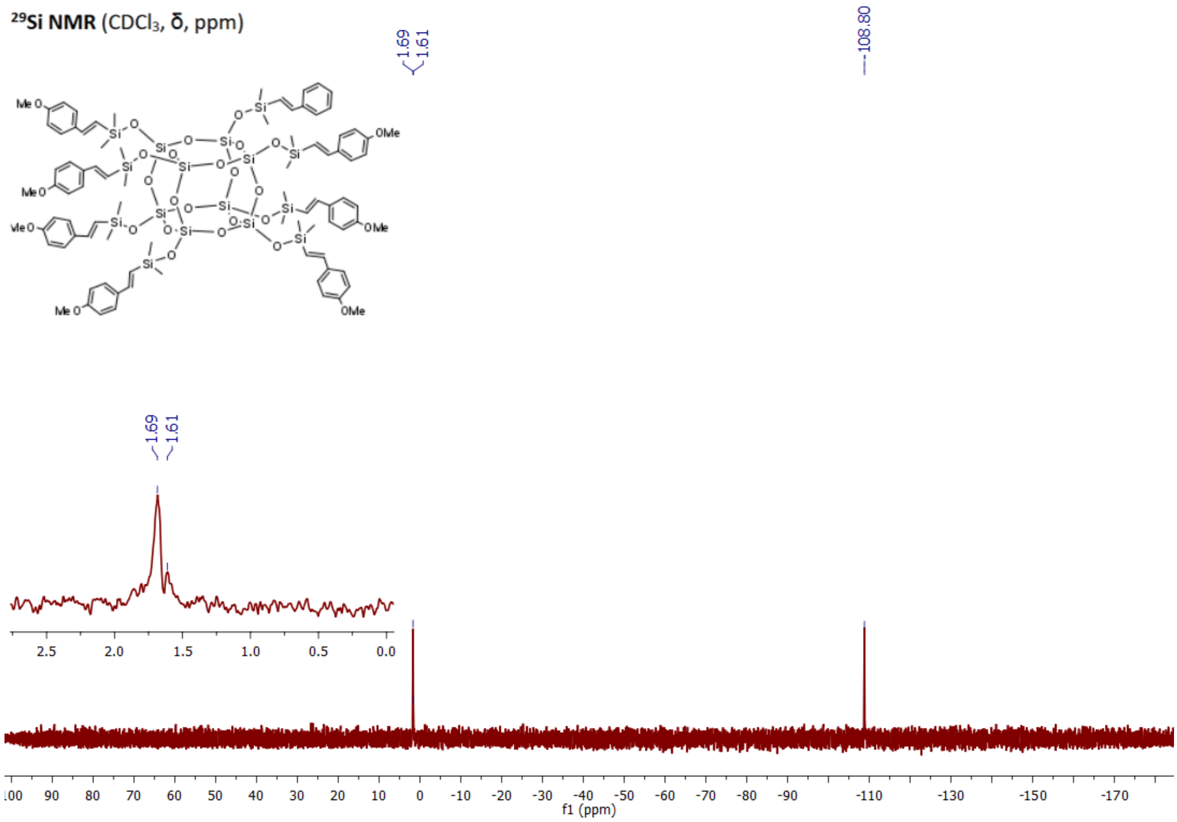

Figure S45. ^{29}Si NMR spectrum of 7e$_7$a$_1$.

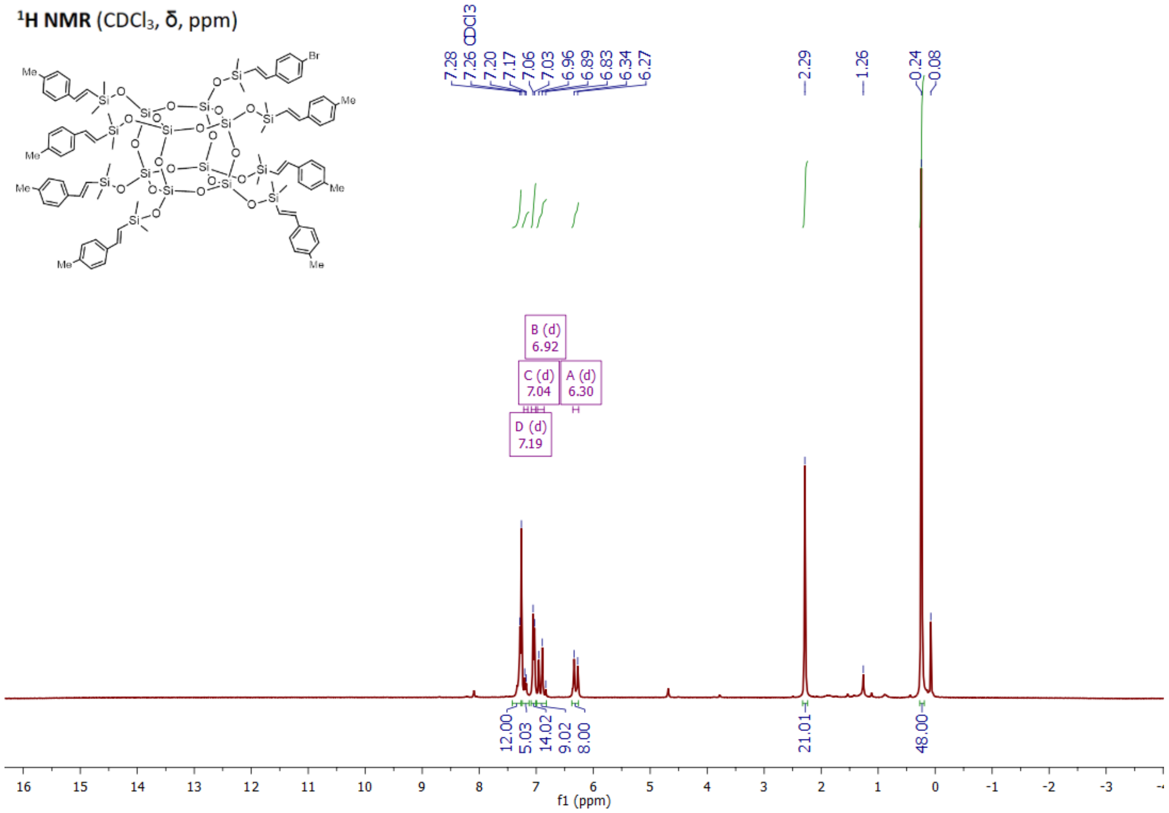

Figure S46. ^{1}H NMR spectrum of $7b_7f_1$.

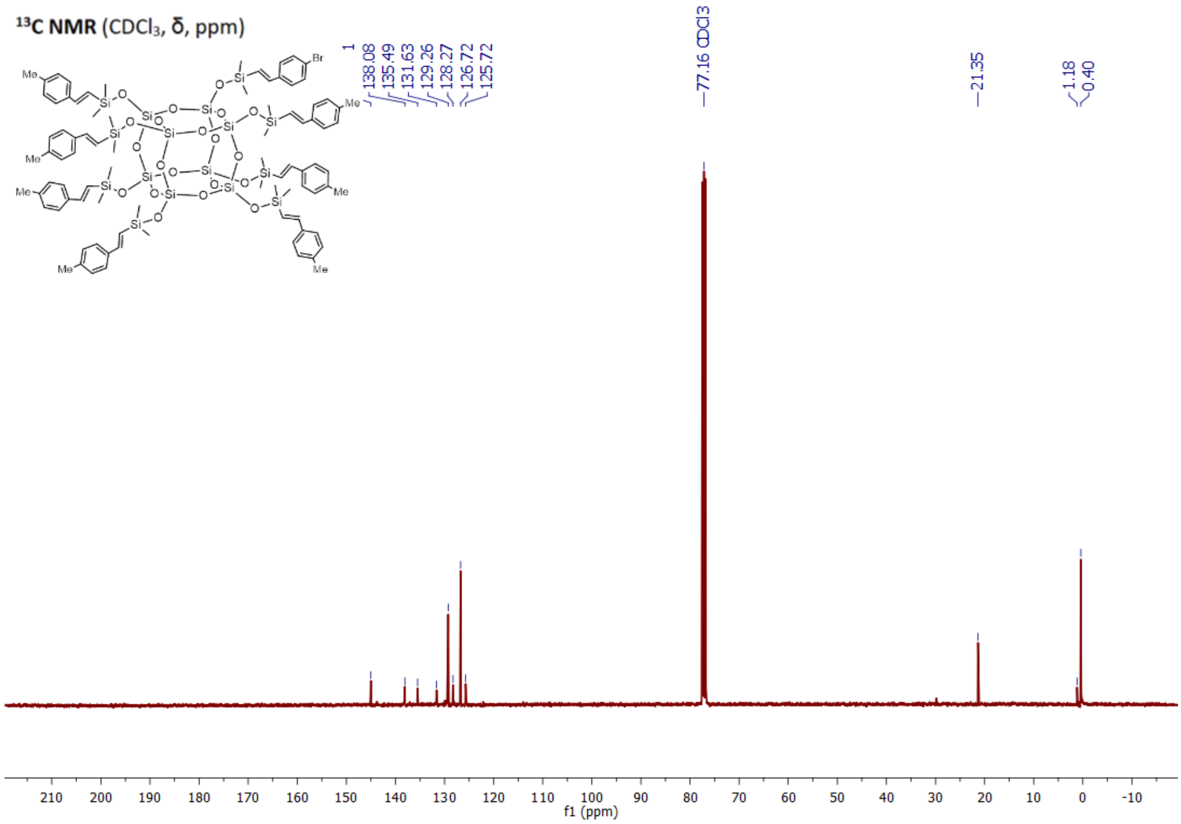

Figure S47. ^{13}C NMR spectrum of $7b_7f_1$.

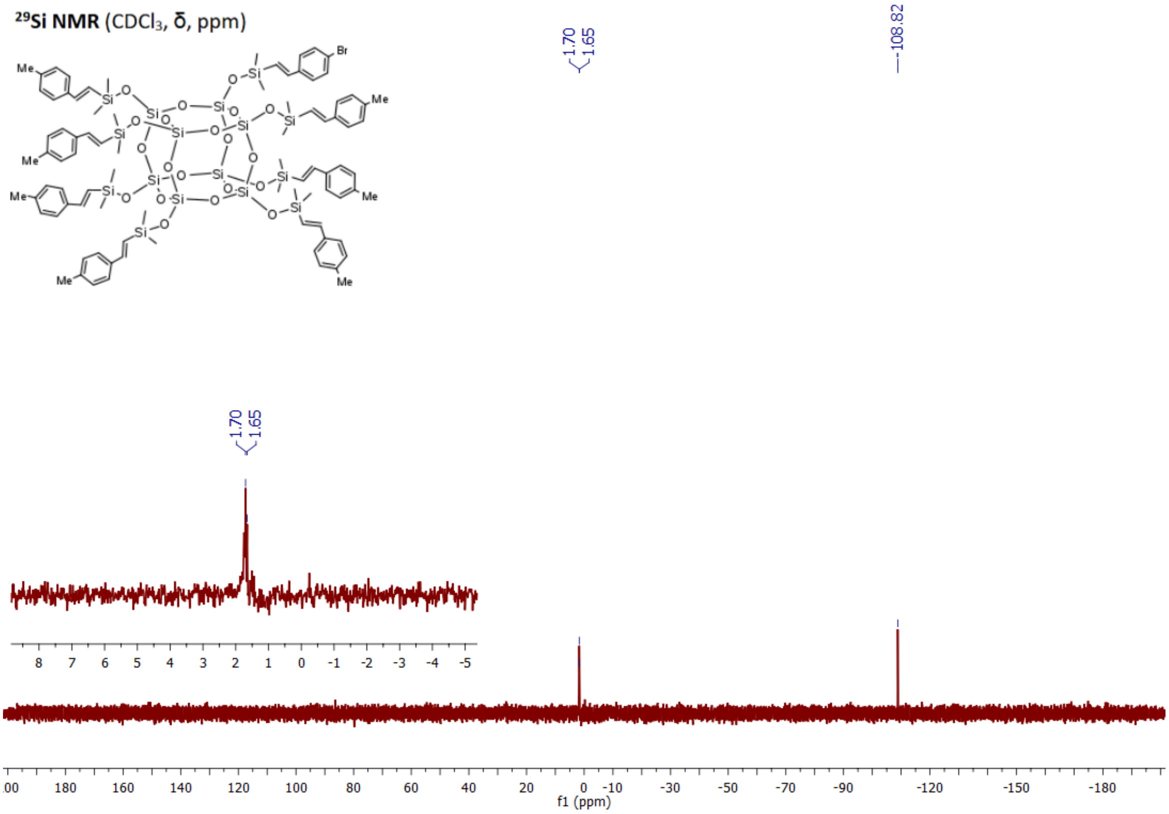

Figure S48. ^{29}Si NMR spectrum of 7b$_7$f$_1$.

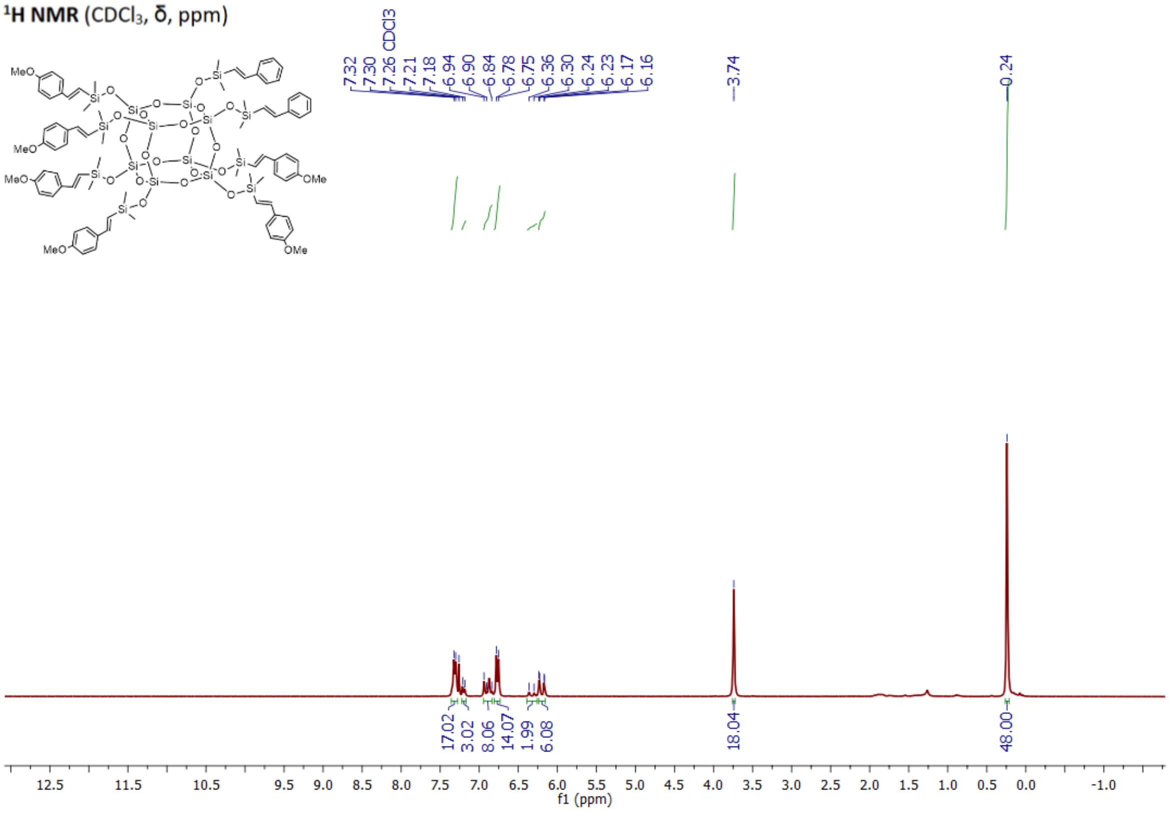

Figure S49. ^{1}H NMR spectrum of 7e$_6$a$_2$.

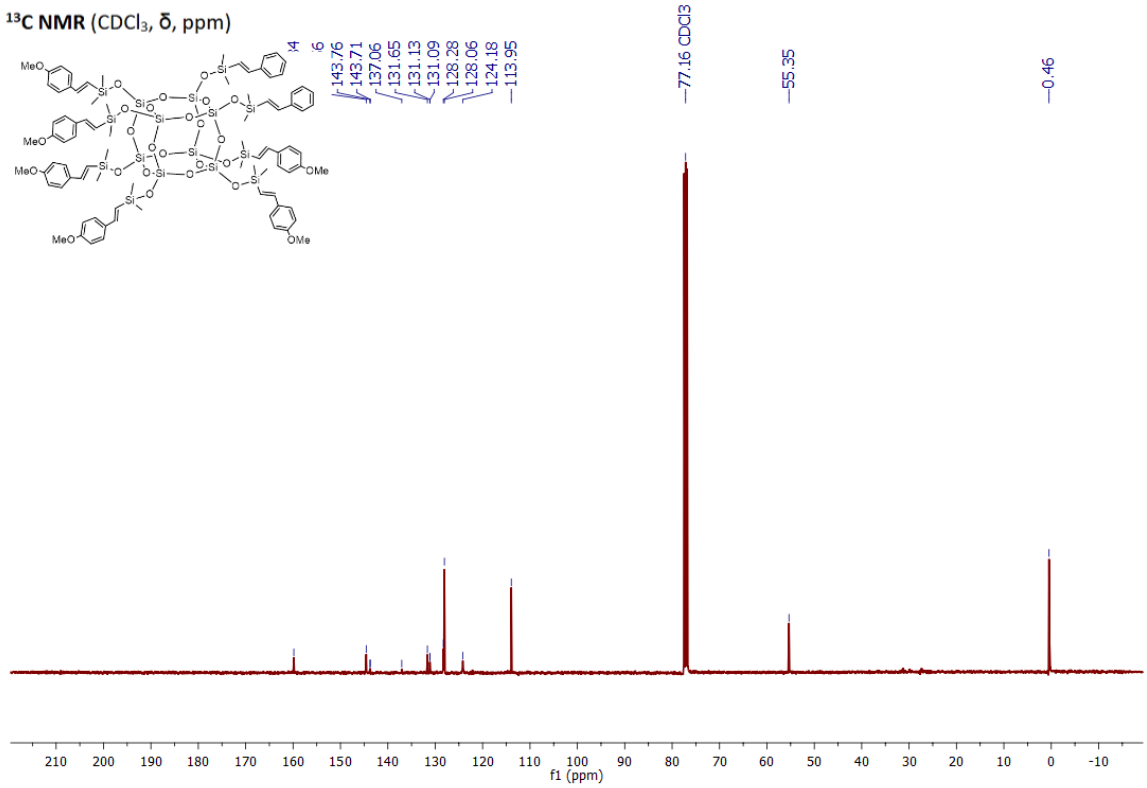

Figure S50. ^{13}C NMR spectrum of 7e$_6$a$_2$.

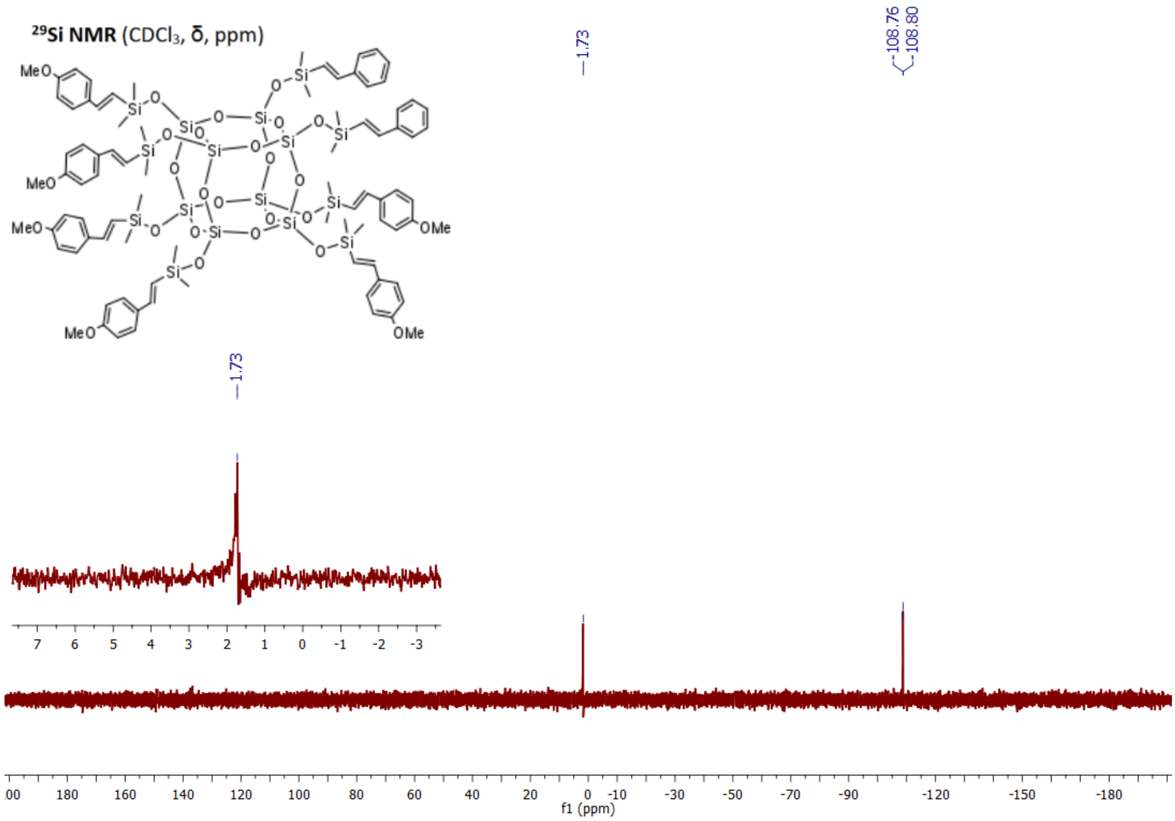

Figure S51. ^{29}Si NMR spectrum of 7e$_6$a$_2$.

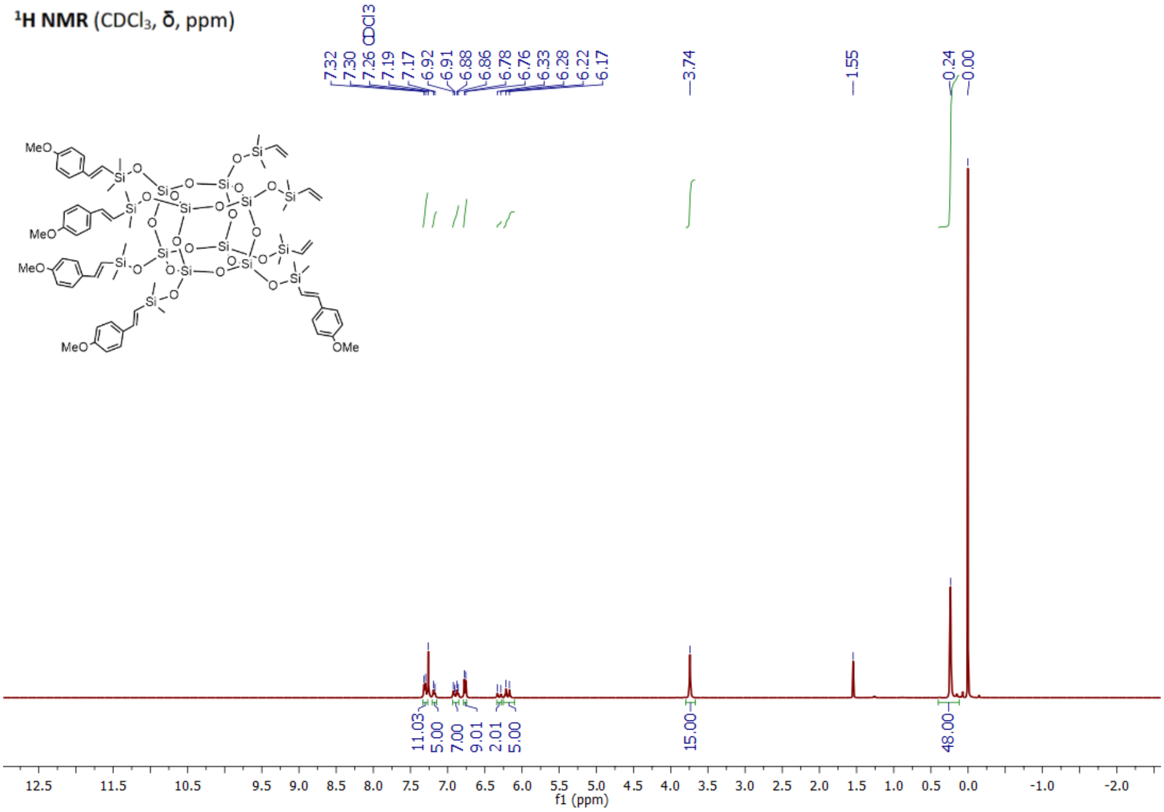

Figure S52. ¹H NMR spectrum of 6e5.

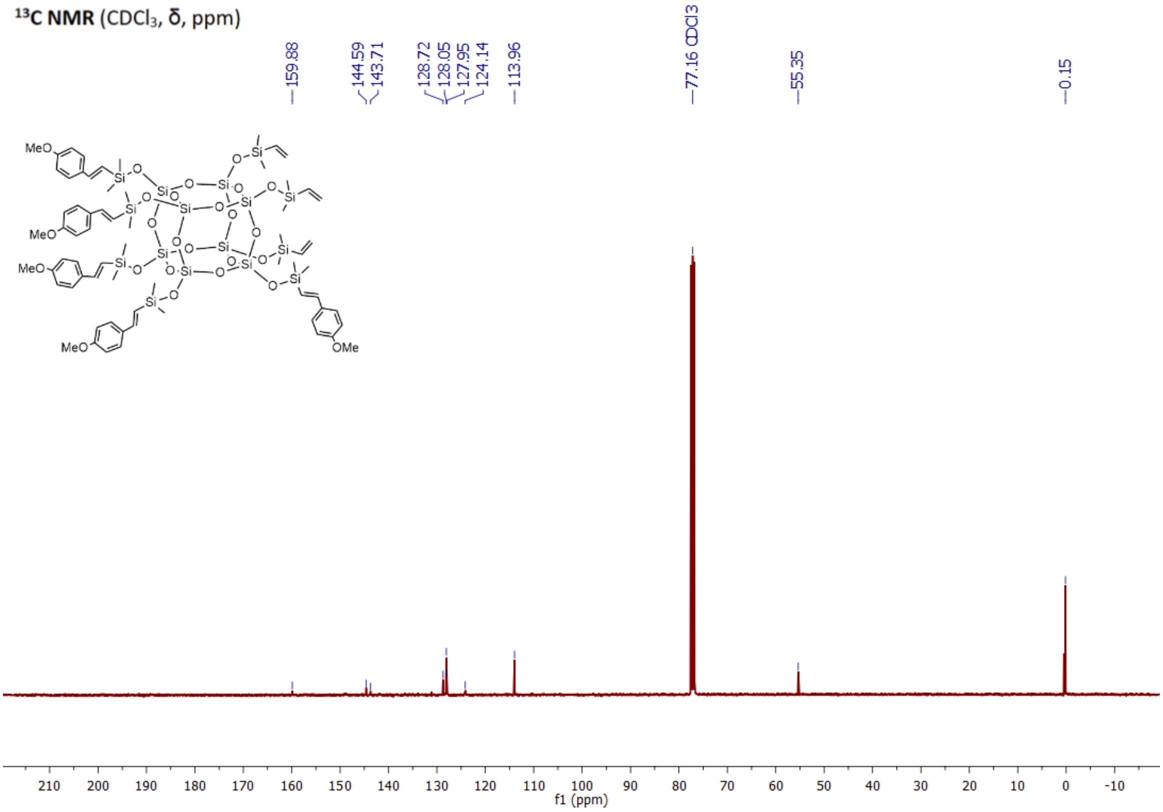

Figure S53. ¹³C NMR spectrum of 6e5.

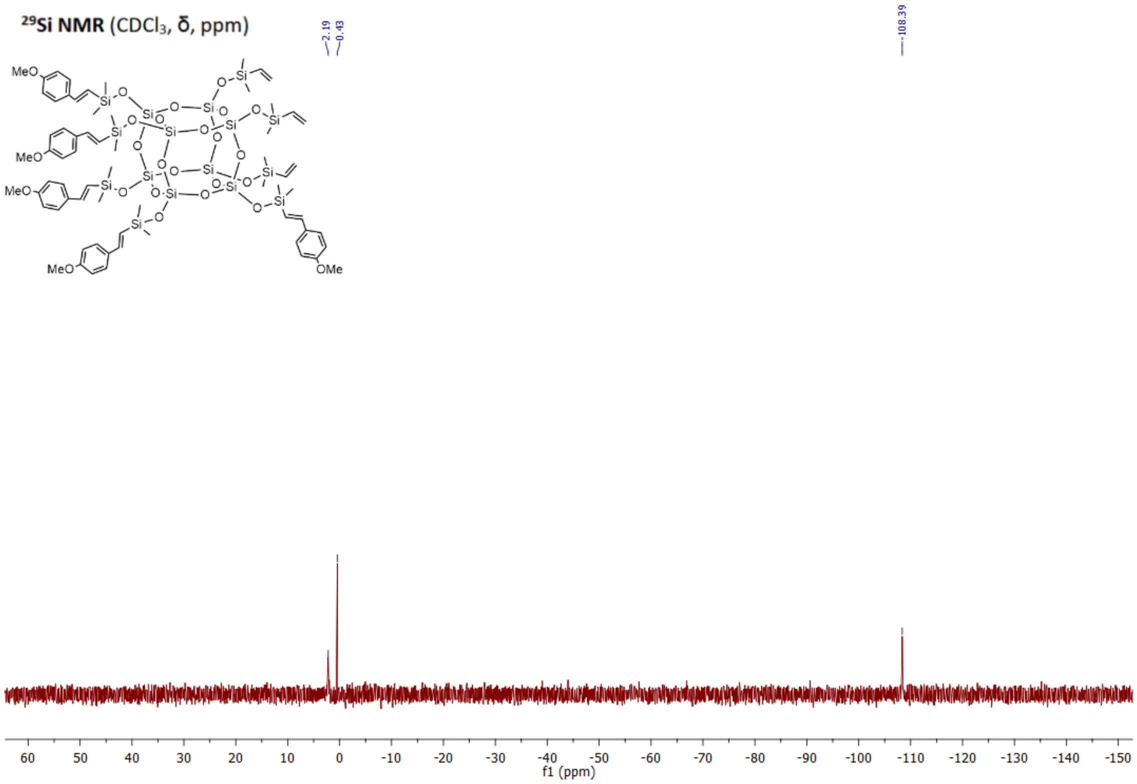

Figure S54. ^{29}Si NMR spectrum of 6e5.

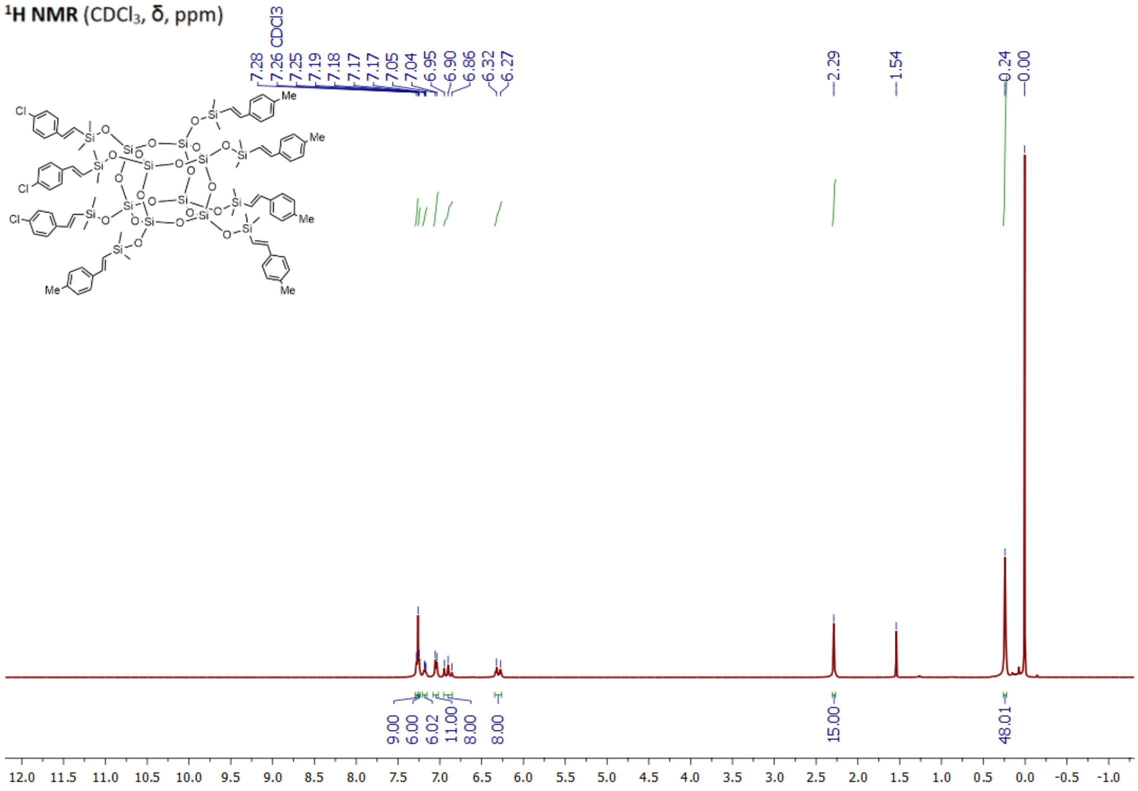

Figure S55. ^{1}H NMR spectrum of 7b5c3.

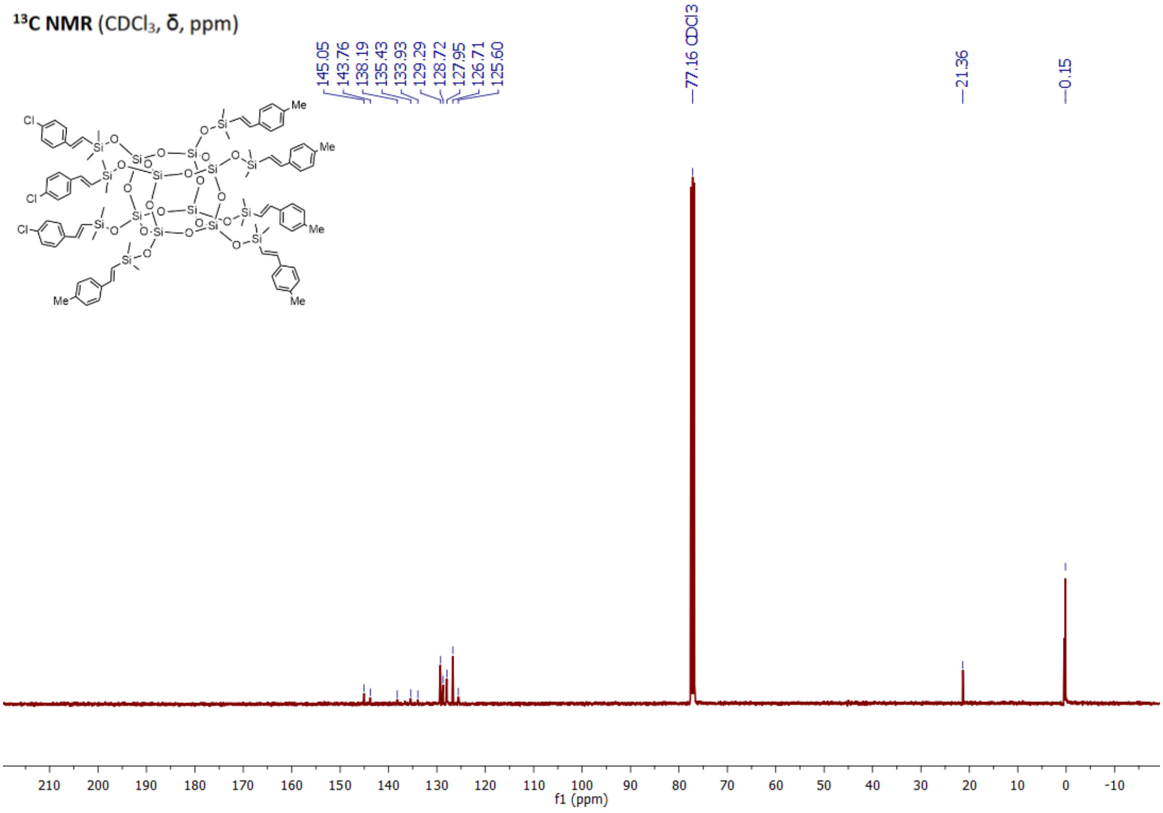

Figure S56. ^{13}C NMR spectrum of 7b5c3.

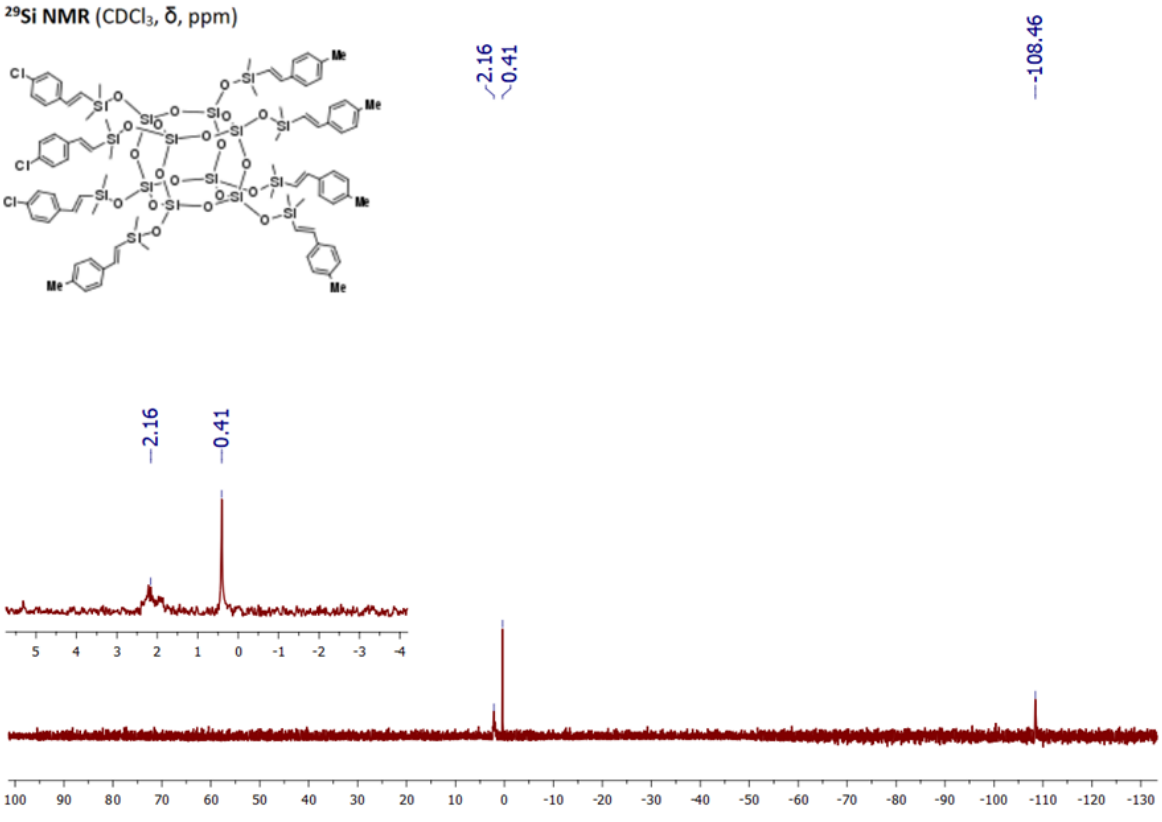

Figure S57. ^{29}Si NMR spectrum of 7b5c3.

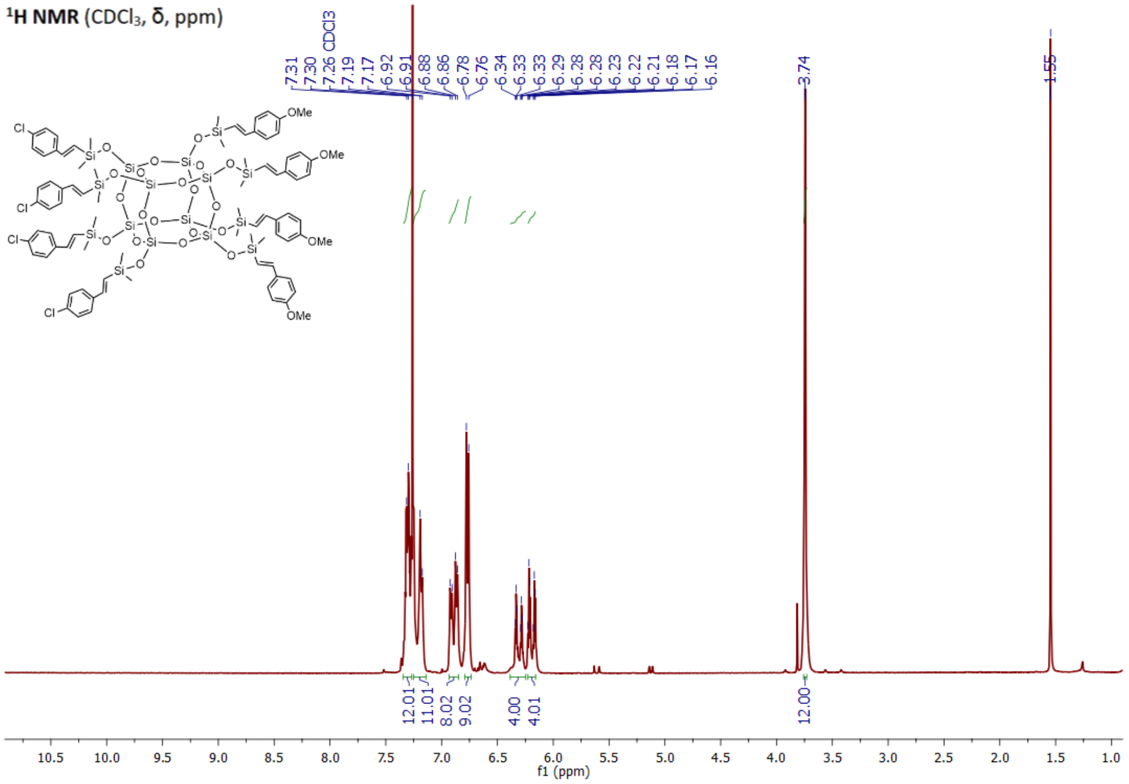

Figure S58. ^{1}H NMR spectrum of 7e4c4.

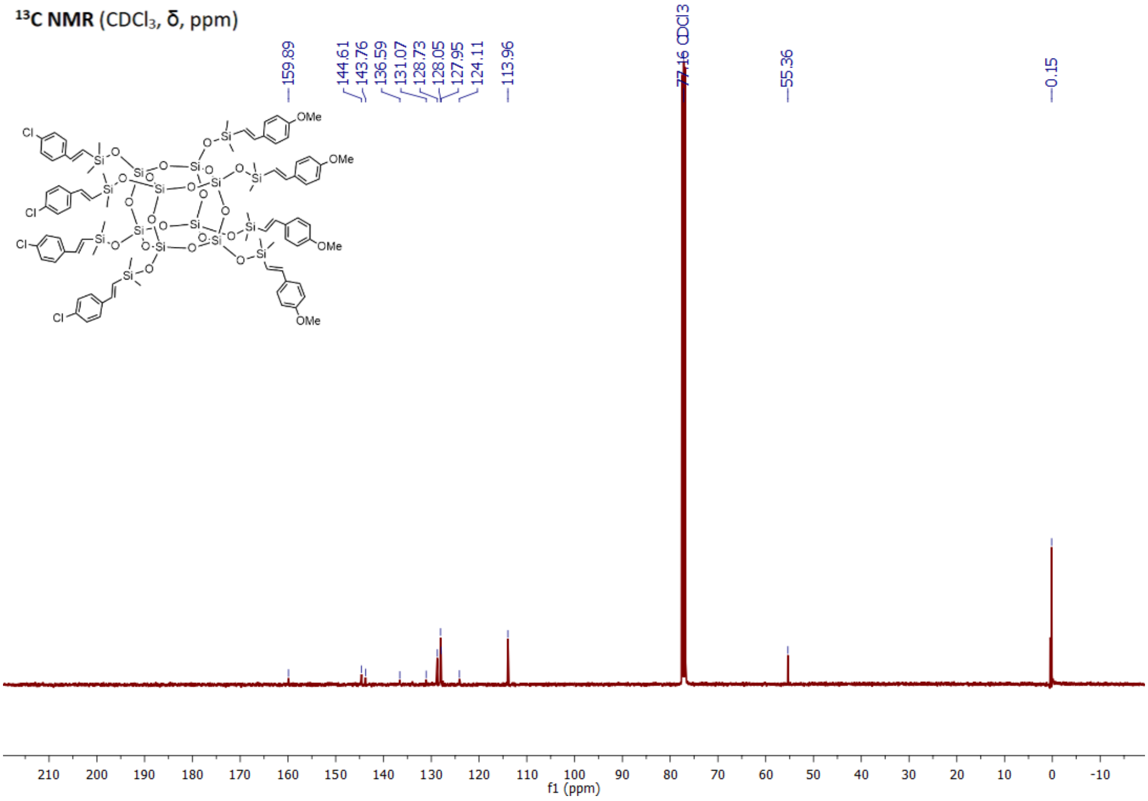

Figure S59. ^{1}H NMR spectrum of 7e4c4.

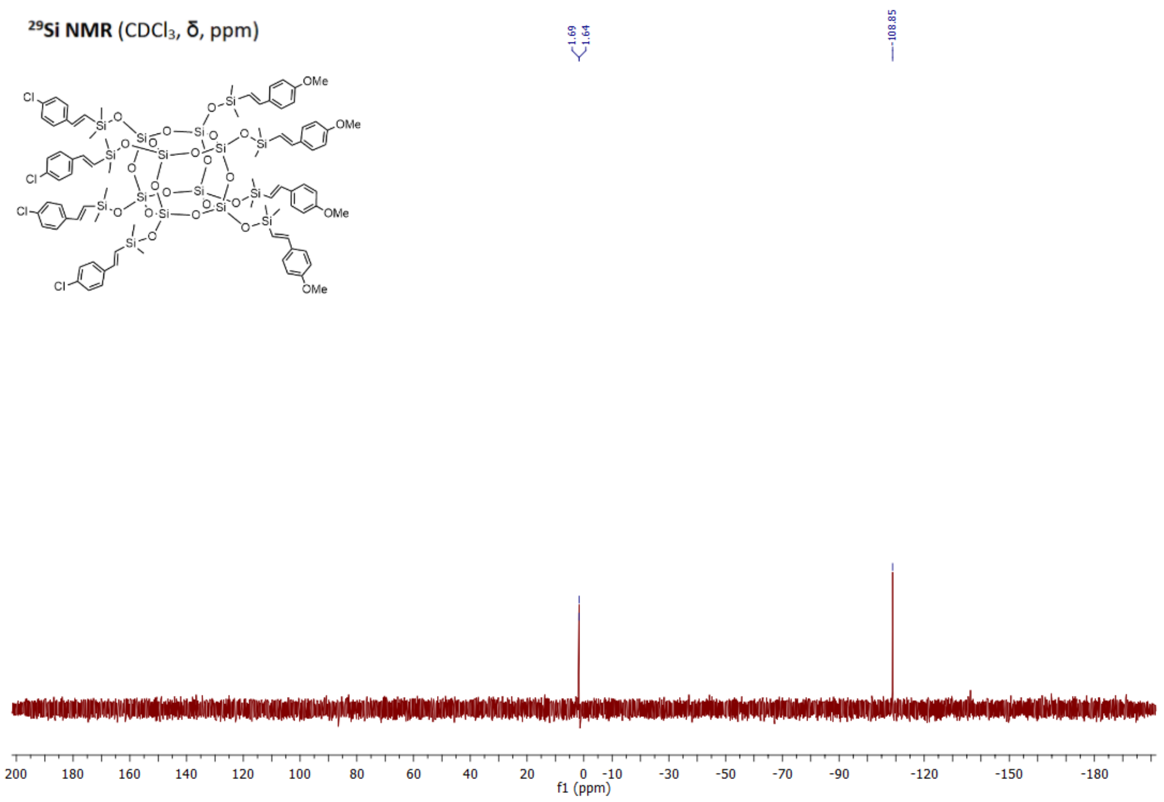

Figure S60. ^{29}Si NMR spectrum of 7e$_4$c$_4$.

4. MALDI-TOF spectra of selected compounds

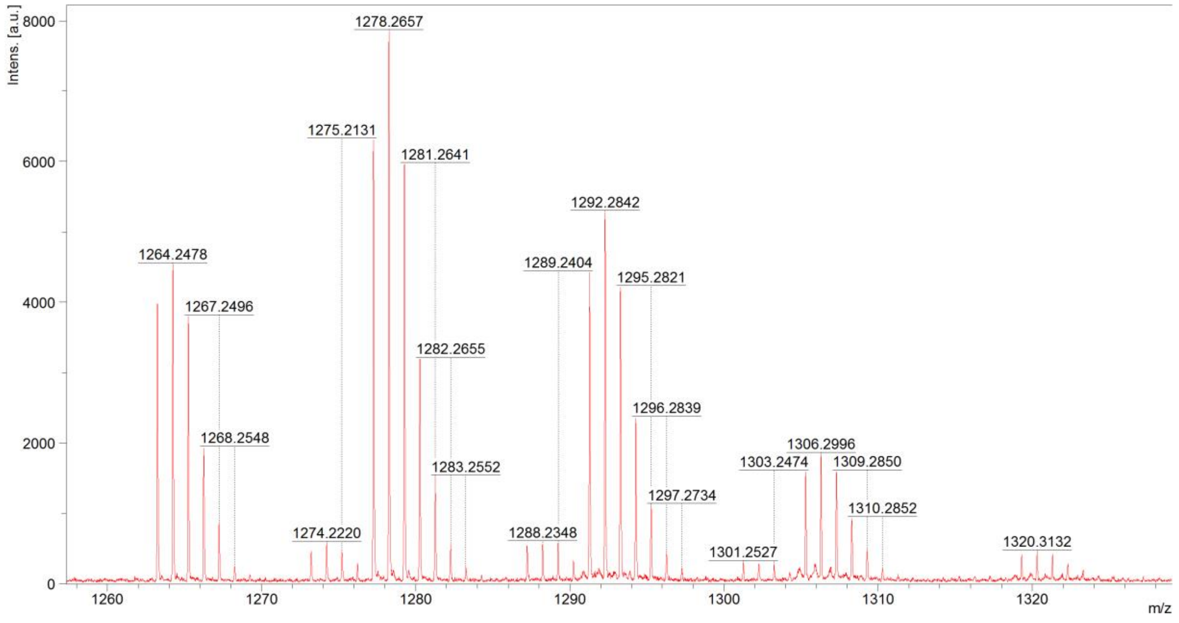

Figure S61. MALDI-TOF spectra of compounds formed in the reaction dedicated to obtain 4b$_7$a$_1$.

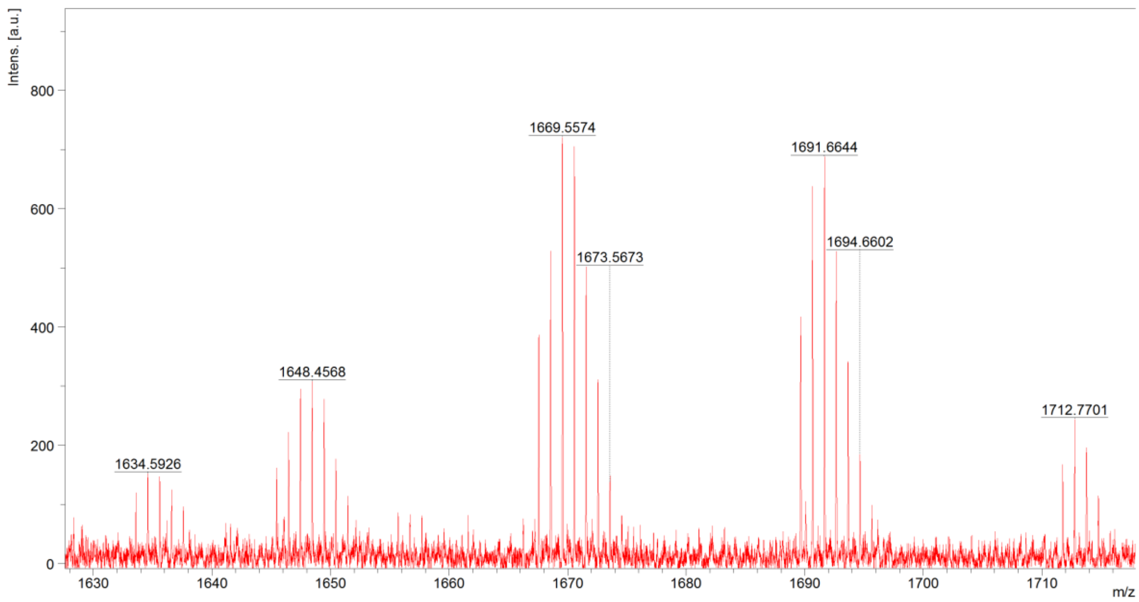

Figure S62. MALDI-TOF spectra of compounds formed in the reaction dedicated to obtain 4d6c2.

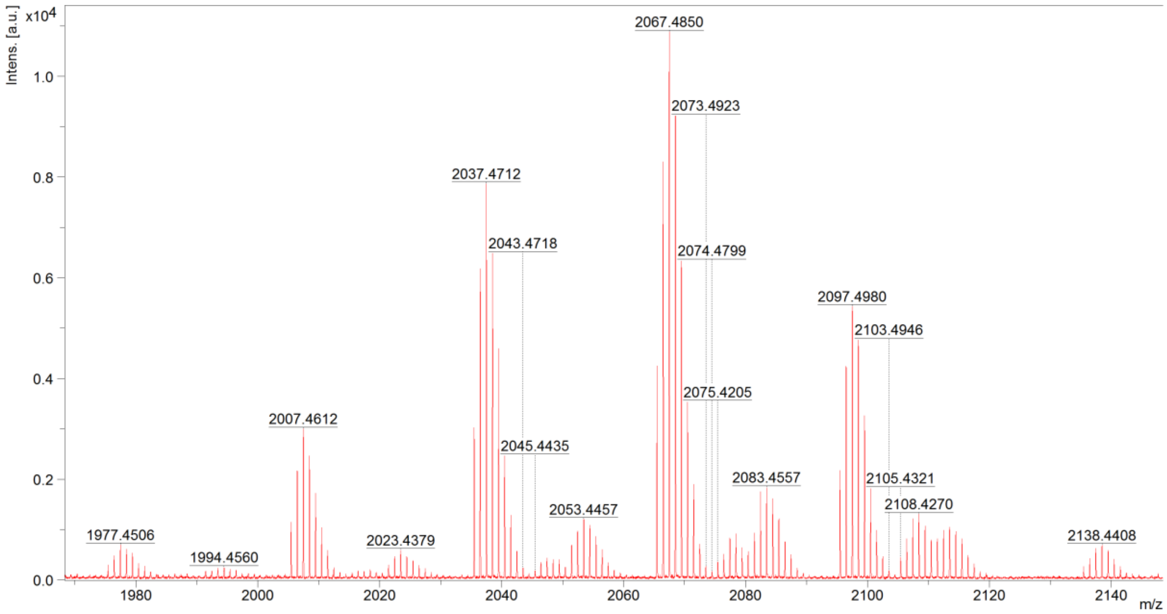

Figure S63. MALDI-TOF spectra of compounds formed in the reaction dedicated to obtain 7e7a1.

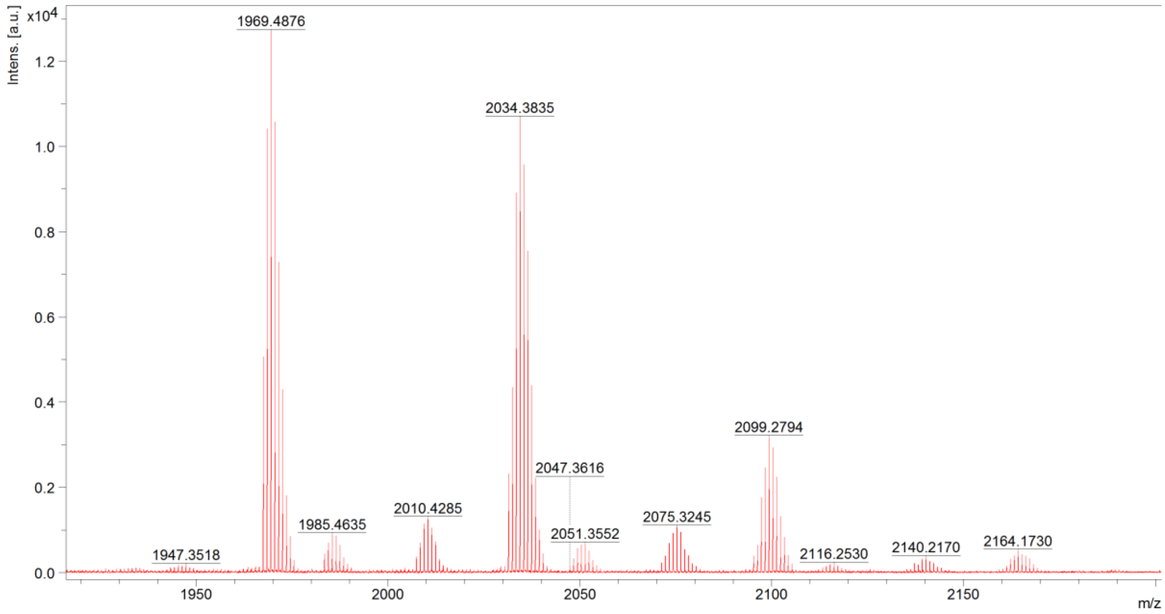

Figure S64. MALDI-TOF spectra of compounds formed in the reaction dedicated to obtain $7b_7f_1$.
